# Ancient intron insertion sites and palindromic genomic duplication evolutionally shapes an elementally functioning membrane protein family

**DOI:** 10.1186/1471-2148-7-143

**Published:** 2007-08-20

**Authors:** Motoko Tanaka-Kunishima, Yoshihiro Ishida, Kunitaro Takahashi, Motoo Honda, Takashi Oonuma

**Affiliations:** 1 Department of Medical Physiology, Meiji Pharmaceutical University, Noshio 2-522-1, Kiyose, Tokyo, MZC204-8588, Japan

## Abstract

**Background:**

In spite of the recent accumulation of genomic data, the evolutionary pathway in the individual genes of present-day living taxa is still elusive for most genes. Among ion channels, inward K^+ ^rectifier (IRK) channels are the fundamental and well-defined protein group. We analyzed the genomic structures of this group and compared them among a phylogenetically wide range with our sequenced *Halocynthia roretzi*, a tunicate, IRK genomic genes.

**Results:**

A total of 131 IRK genomic genes were analyzed. The phylogenic trees of amino acid sequences revealed a clear diversification of deuterostomic IRKs from protostomic IRKs and suggested that the tunicate IRKs are possibly representatives of the descendants of ancestor forms of three major groups of IRKs in the vertebrate. However, the exon-intron structures of the tunicate IRK genomes showed considerable similarities to those of *Caenorhabditis*. In the vertebrate clade, the members in each major group increased at least four times those in the tunicate by various types of global gene duplication. The generation of some major groups was inferred to be due to anti-tandem (palindromic) duplication in early history. The intron insertion points greatly decreased during the evolution of the vertebrates, remaining as a unique conservation of an intron insertion site in the portion of protein-protein interaction within the coding regions of all vertebrate G-protein-activated IRK genes.

**Conclusion:**

From the genomic survey of a family of IRK genes, it was suggested that the ancient intron insertion sites and the unique palindromic genomic duplication evolutionally shaped this membrane protein family.

## Background

Although a vast amount of data on the genomic structures of genes of major phyla in animal, plant, and microbial kingdoms has been accumulated as a result of many recent successful projects of whole genome shotgun DNA sequencing of biologically and medically important taxa [1-7], the evolutional pathway and physiological significance of individual genome gene structures, such as the conservation of intron insertions, duplication of genes, and development of gene regulatory sequences of the present-day living taxa are still elusive and unclear for most genes. Recent large population studies derived from the established genome databases have promoted our understanding of intron significance, favoring the exon theory or the intron early theory, though many points remain to be discussed and clarified [8-12]. Similarly, we know that gene duplication by the tandem repeat or chromosomal duplication must be the major evolutional power to drive simple to complex living systems at least in the vertebrate clade [13-17]. However, for individual genes the exact evidence is still in short availability because of the gene divergence of the whole genome chromosomes in the present-day organisms on the evolutionary pathway from the ancestor organisms, though the recent excellent whole genome studies on the *Saccharomycetes *have greatly advanced our understanding about these matters [18-20]. Further, although the eukaryotic genomic complexity may be initiated by the non-adaptive fixation of genetic drift resulting from a reduction of the effective population size [21], at least in the secondary development, it is plausible to correlate the evolution of the genomic structure to the adaptation of organisms to new environmental niches, such as, the functional evolution of the proteins as the gene products [22]. However, little is known regarding the mechanisms except the proposal that the borders of domains in the protein are represented by the exon boundaries, thus being a proposal of physiological support for the exon theory [23-25].

Ion channels are one of the major and important protein groups functioning for regulating the intra- and extracellular ionic environments and for the signal transduction of intracellular and intercellular systems [26]. Among ion channels, the inward K^+ ^rectifier (IRK) channels comprise the most fundamental and simple protein group, having two transmembrane segments and one pore-forming region [27,28]. The physiological functions are well studied in both protostomia and deuterostomia, and almost all members of genes have been sequenced at least in the mammals, such as human, mouse, and rat, by cDNA cloning or by genome projects [26,28].

Recently, we have sequenced three *Halocynthia roretzi *IRK genomic clones, of which cDNA clones have been known [29,30], being TuIRKA, TuGIRKAa, and TuGIRKB clones. Furthermore, the urochordate, *Ciona intestinalis*, genome projects [4] have allowed a genomic survey of tunicate IRK channel genes for molecular evolutionary analysis in comparison with the genes of both deuterostomal and protostomal clades. This has occurred because the urochordate genes have been known to locate at the branching point for the early vertebrate divergence within the deuterostomal clades, as shown in the recent exhaustive comparative study of ion channel genes viewed from the annotation of the *Ciona *unochordate genes [31].

In the present study, we attempted to analyze the genomic structures of a group of proteins, which are definitely defined functionally, and to compare them among a phylogenetically wide range, including protostomal to deuterostomal clades. We also aimed to elucidate the functional and evolutional significance of intronic insertion, intronic preservation, gene duplication on the chromosomes, and the relationship between the genome structure and the expressed protein structure or physiological function of the organism.

## Results

### Genomic sequences of tunicate inward K^+ ^rectifier genes

**Figure 1:**
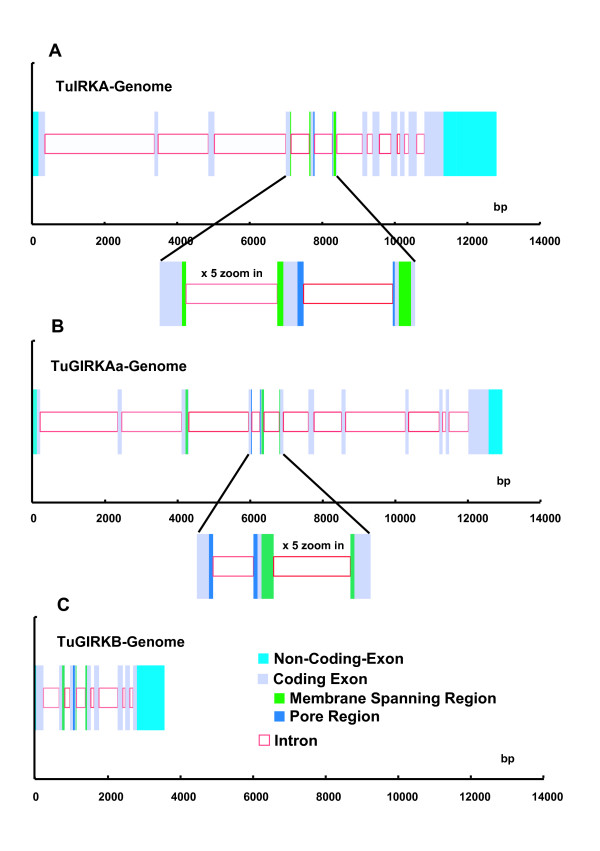
**Illustration of genomic structures of three *Halocynthia *inward K^+ ^rectifier genes**. ***(A) ***Elementary inward K^+ ^rectifier, TuIRKA genome sequence. ***(B) ***G-protein-activated inward K^+ ^rectifier, TuGIRKAa genome sequence. ***(C) ***TuGIRKB genome sequence. In the figures, gray-filled squares and red open squares indicate coding exons and introns, respectively, in the coding regions. **Sky blue **indicates noncoding exons. **Green **and **blue **represent membrane-spanning and pore regions within the coding exons. The inserts in the respective figures represent expanded sequences of the regions containing the membrane-spanning and pore segments. Scales in the graphs are bp.

**Figure 2:**
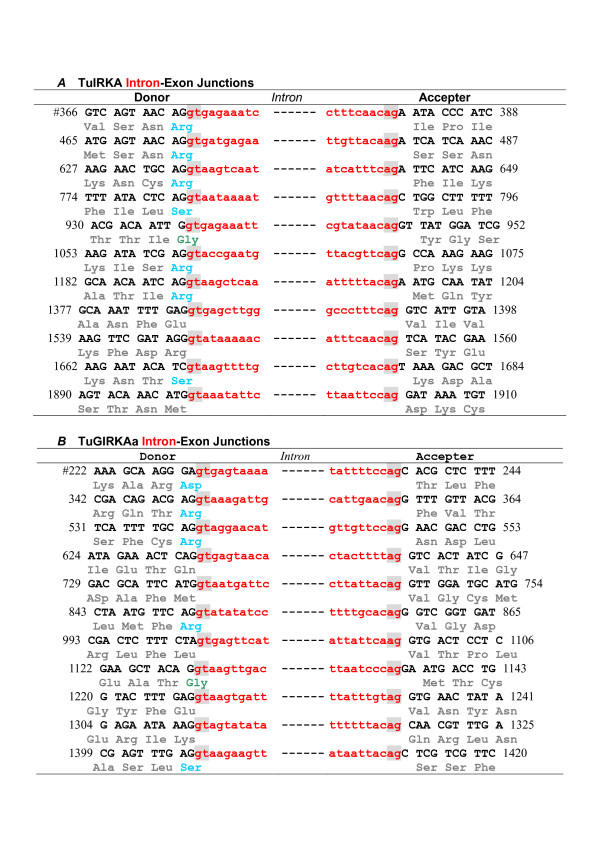
**Intron-Exon junctions in *Halocynthia roretzi *Inward K^+ ^rectifier genomes**. ***(A) ***Elementary IRK, TuIRKA genome. ***(B) ***G-protein activated IRK, TuGIRKAa genome. **Black **and **red **characters indicate the nucleotides in coding regions and the ones in the intronic regions, respectively. **Gray **triplet characters represented amino acid (AA) codes, which were not inserted with introns. The **phase 0 intron**s were found between those AA pairs. **Green **and **sky blue **triplet characters represented AA codes, which were inserted by **phase 1 introns **between the first and second nucleotides in the codons, and by **phase 2 introns **between the second and third nucleotides in the codons, respectively. Numbers at both sides of the nucleotide sequences are those assigned in the reported cDNAs. For another G-protein activated IRK, TuGIRKB genome, the intron-exon junctions are illustrated in an additional file [see Additional file 4].

We determined genomic sequences of three inward K^+ ^rectifier genes, TuIRKA, TuGIRKAa, and TuGIRKB, cloned from a genomic library of a tunicate, *Halocynthia roretzi *[see Additional file 1]. With previously reported respective cDNA sequences [29,30] [see Additional file 2], their exons and introns in their coding regions were precisely determined [see Additional files 1 and 2]. These sequences covered entire coding regions, including all introns, 5' upper stream regions, and most of the 3' lower stream regions (Fig. 1 and [see Additional file 3]). All intron exon junctions, which existed in the coding regions, revealed the mammalian-conserved splicing characteristic sequences, such as the GT of splice donor sites at the initiation of introns and the AG of splice acceptor sites at the 3' terminal of respective introns (Fig. 2 and [see Additional file 4]). The average exon length was about 100 bp and was considerably short in comparison with those found in mammalian Kcnj genes. The average intron length was about 500 bp; thus these genomic sequences revealed considerably large numbers of intron insertions, from 8 to 11, within a rather short whole coding length of about 1000 bp of IRK genes [see Additional file 1]. Although the insertions were apparently evenly distributed among the coding regions, all three IRK genomes revealed characteristic intron insertions in the functionally important and thereby the strongly conserved sequence portions of the membrane-spanning regions and the ion pore regions or limes of those regions (Fig. 1).

### The phylogenetic tree and a comparison of Halocynthia IRK AA sequences with those of other animal IRK genes

**Figure 3:**
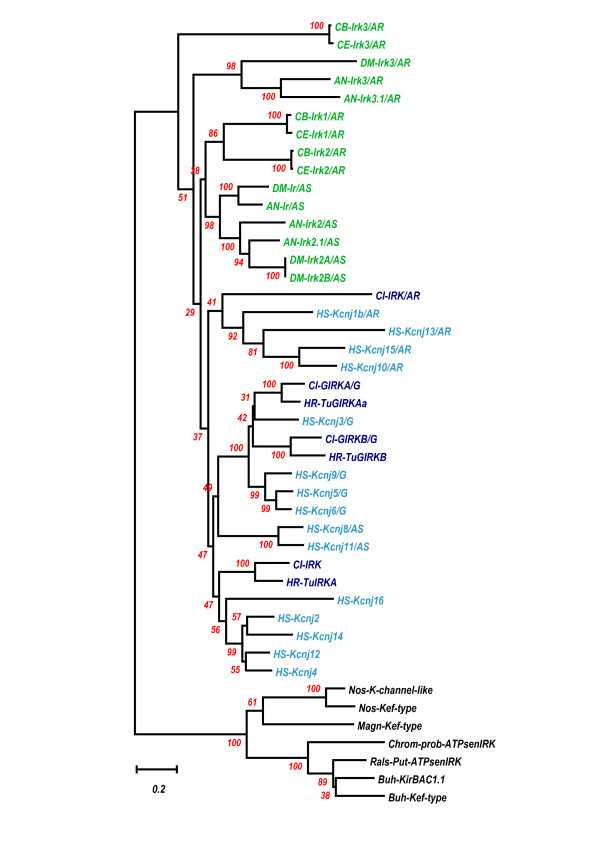
**Phylogenetic tree derived from 44 IRK AA sequences**. Forty-four sequences included 3 *Halocynthia roretzi *IRKs, 4 *Ciona *IRKs, 15 human IRKs, 3 *Caenorhabditis elegans *IRKs, 3 *Caenorhabditis briggsae *IRKs, 4 *Drosophila melanogaster *IRKs, and 5 *Anopheles gambiae *IRKs. These amino acid (AA) sequences were referred from JGI *Ciona *genome database, from NCBI GenBank and Genome database, or from Ensembl database. The obtained AA sequences from the seven taxa were aligned using ClustalX1.83 program. The gaps within nonpreserved regions were carefully deleted manually by the BioEdit program. Then the phylogenetic tree derived from the aligned data was made by the Neighbor Joining Method in the Mega3 v3.1 program, using the Amino Poisson correction model with Gamma-distributed Rates among sites (Gamma parameter 2.0) and Bootstrap as the test of inferred branches (Repetition 500), including 295 sites with a pair-wise deletion of Gap/Missing Data. As an outgroup, the seven bacterial IRK-like proteins were chosen, including KirBAC1.1, of which the molecular structure was recently determined by X-ray [32]. Those bacterial AA sequences were also referred from an NCBI microbial genome database. The scale for tree-branch length is 0.2 mutation per AA site and is illustrated at the lowermost part of the figure. Abbreviations: IRK, elementary IRK; IRK/G, G-protein-activated IRK; IRK/AR, ATP-regulated IRK; IRK/AS, ATP-sensitive IRK like SU-receptor-coupled IRK. Nossp, *Nostoc sp.; *Nospc, *Nostoc punctiforme*; Magn, *Magnetospirillum magnetotacticum*; Chrom, *Chromobacterium violaceum*; Buhpd, *Burkholderia pseudomallei*; Buhfg, *Burkholderia fungorum*; Rals, *Ralstonia solanacearum*. Kef-type K^+ ^transport system. Prob-ATPsenIRK, Probable ATP-sensitive IRK. Put-ATPsenIRK, Putative ATP-sensitive IRK. The scale and abbreviations are applied in this and the following figures.

**Figure 4:**
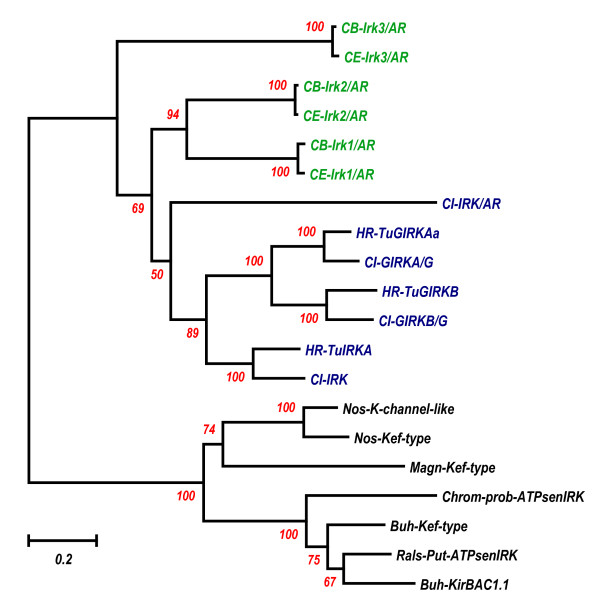
**Phylogenetic tree derived from selected 20 IRK AA sequences**. To show the major phylogenetic evolution of IRK genes more visually, the 20 sequences were selected from the 44 IRK data used for the previous Fig. 3, that is, three *Halocynthia roretzi *IRKs, four *Ciona *IRKs, three *Caenorhabditis elegans *IRKs, and three *Caenorhabditis briggsae *IRKs. The seven bacterial IRK-like proteins were taken as the outgroup, which was the same as in Fig. 3. The position of KirBAC1.1 was underlined. Alignment, phylogenetic tree building, and Bootstrap for the internal branch test were the same as in Fig. 3. The gaps within nonpreserved regions were carefully deleted manually by the BioEdit program. Then the phylogenetic tree derived from the aligned data was made by the Neighbor Joining Method in the Mega3 v3.1 program, including 269 sites with pairwise deletion of Gap/Missing Data. **In **Additional file 5, **Excel data of intron insertion in various IRK genomes, **intron numbers, sizes, and locations for 123 IRK genes used for the analysis of the present study were listed. The identification numbers in GenBank or Ensembl for the genes and their locations in the chromosomes were also included. [see Additional file 5]

Thanks to many recent whole genome projects, there are many numbers of IRK genomic sequences, which are determined over their whole genome regions. Among them, as the first step, the genomic sequences of the same tunicate *Ciona *IRK genes, human IRK genes, *Caenorhabditis elegans *IRK genes, *Drosophila melanogaster*, and *Anopheles gambiae *genes were compared with the sequences of *Halocynthia roretzi *IRK genes. With the amino acid (AA) sequences inferred from the JGI Ciona genome database, from the NCBI GenBank and Genome database, or from the Ensembl database, the AA sequences of all IRK proteins from the seven taxa were aligned by using the ClustalX1.83 program, and their phylogenetic tree was made by the Neighbor Joining Method in the Mega3 v3.1 program (Fig. 3). As the outgroup, the seven bacterial IRK-like proteins were chosen, including KirBAC1.1, of which the molecular structure was recently determined by X-rays [32]. Although some branches were not significant according to Bootstrap values, i.e., less than 50%, the tree revealed the following three points. First, the three IRK proteins in *Halocynthia roretzi *revealed corresponding close homologues in *Ciona intestinalis*, respectively. Moreover, one other IRK gene existed in the tunicates. Second, the three types of tunicate IRK, elementary IRK, GIRK, and possibly ATP-regulated IRK, were representatives of three major groups of the human IRK proteins. All protostomic IRKs were grouped differently from *Halocynthia *IRKs, though they may show some similarity to a putative *Ciona *ATP-regulated type IRK. Prokaryotic IRKs are all evolutionally different from those eukaryotic ones. To illustrate the major phylogenetic evolution of IRK genes more simply and clearly, we aligned the AA sequences of tunicate, *Caenorhabditis*, and bacterial IRK proteins, and the tree was made, as shown in Fig. 4. All internal branches in the tree were verified by a Bootstrap test, suggesting that the elementary IRK group and G-protein-activated group diversified from the putative ATP-regulated type in the tunicate clade later than the deuterostomic and protostomic diversification in the ancestor IRKs, possibly one of the putative ATP-regulated types. The results further confirmed that the IRKs of protostomic clade were different from those of the deuterostomic clade, but they evolved after the eukaryotic diversification from prokaryotic evolution.

### Comparison of intron exon structures of IRK genes between protostomic and deuterostomic clades

**Figure 5:**
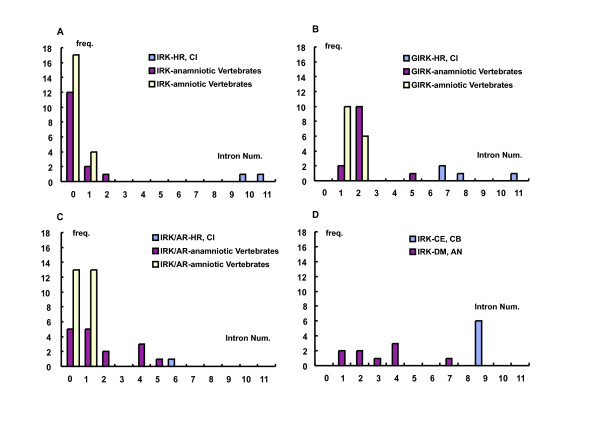
**Frequency histograms of intron numbers in all 108 IRK genes examined**. Figure ***A***, ***B*, **and ***C ***represent the histograms for three major groups of vertebrate IRKs, that is, ATP-regulated, elementary, and G-protein-activated IRK groups, respectively. Anamniotic vertebrates mean fish and amphibians, and amniotic birds and mammals. Abscissa, Intron numbers. Ordinate, Number of genes, which showed numbers of intron insertions in the coding regions as indicated on the abscissa. Figure ***D ***is the similar histogram for IRK genes of protostomic clade, that is, *Caenorhabditis elegans *and *brigssae*, and *Drosophila melanogaster *and *Anofeles gambiae*.

**Figure 6:**
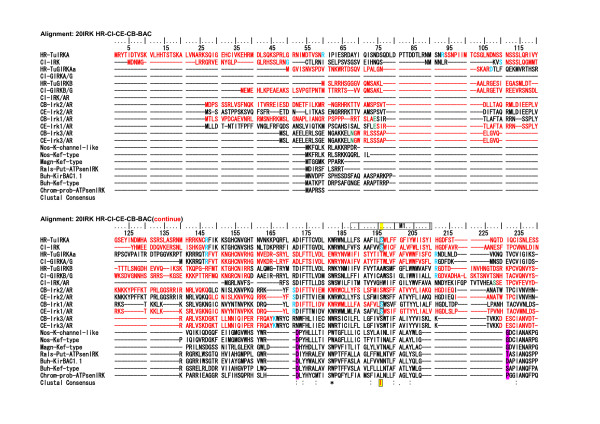
**Alignment of 7 tunicate, 6 *Caenorhabditis*, and 7 bacterial IRK AA sequences**. Alignments were performed by using the Clustalx1.83 program. No gaps were deleted. Intron-intervening sites are illustrated by changes in colors of AA code characters. **Red **to **black **transition or vice versa represents phase 0 intron insertion (the case for the intron intervention between two neighboring AA codes). **Green **and **sky blue **indicate phase 1 intron (intron insertion between first and second nucleotides of an AA code), and phase 2 intron (between second and third nucleotides in an AA code) insertions, respectively. Characters with a **yellow background **and **boxed **indicate the conservation of intron sites between tunicate and *Caenorhabditis*. They were also noted by **red rods **in the lowest Clustal Consensus line. **Purple background **characters indicate either edges of intrinsic gaps in the bacterial AA sequences corresponding to some intron insertion sites in the *Caenorhabditis*. Upper 7 taxis are tunicates, middle six *Caenorhabditis*, and lower 7 prokaryotes. The lowest line indicates Clustal Consensus with stars and dots.

**Figure 7:**
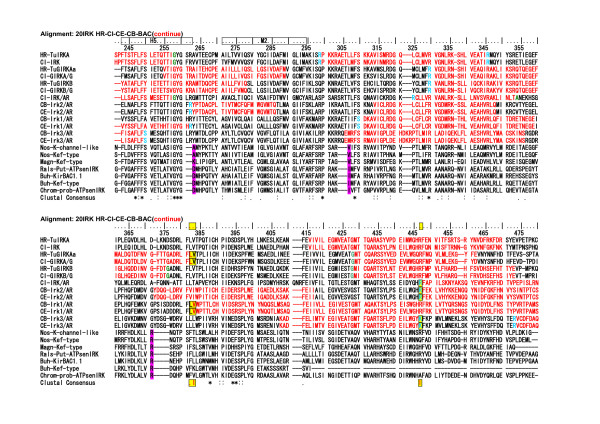
**Alignment of 7 tunicate, 6 *Caenorhabditis*, and 7 bacterial IRK AA sequences (continued from Figure 6.) **The alignments for the rest parts of AA sequences, which are not included in Fig. 6 and 7, are illustrated in an additional file [see Additional file 6].

As described above, 6 to 11 introns were inserted in the coding regions of the *Halocynthia *IRK genes. However, 108 IRK genes of the vertebrate clade, which are extracted from recently established genomic databases listed in Additional file 5 [see Additional file 5], revealed relatively few or no introns in their coding regions. Figure 5 illustrates frequency histograms of intron numbers in all IRK genes examined. Figure 5A, 5B, and 5C represents the histograms for the three major groups of IRKs of the vertebrates, that is, ATP-regulated, elementary, and G-protein-activated IRKs, respectively. The results indicate that the intron numbers were significantly larger in the tunicate IRKs, but they were rather few either in the amniotic or anamniotic vertebrates, though the anamniotic vertebrates might show some larger numbers, especially in the case of ATP-regulated IRKs. For the protostomic clade, the *Caenorhabditis *definitely revealed the large number of 9, but the *Drosophila *and *Anopheles *revealed relatively small numbers of less than 4, as shown in Fig. 5D. In Figure 6 and 7, 7 tunicate, 6 *Caenorhabditis*, and 7 bacterial IRK AA sequences were aligned, and the portions of intron insertion are illustrated [see Additional file 6]. In either tunicates or *Caenorhabditis*, the sites for insertions were strictly conserved between the orthologous protein pairs, at least in the major feature regions of IRK genes. As in the case of tunicates, the *Caenorhabditis *IRK genes revealed insertion sites at the physiologically important membrane regions and the ion pore regions. The further three common insertion sites were found between the tunicates and the *Caenorhabditis *(Fig. 6 and 7, yellow background and boxed characters). Thus in terms of the AA sequences, the *Caenorhabditis *and the tunicate IRKs were separately grouped and evolved differently, but the macroscopic genomic structures seemed to keep commonality between them.

Naturally bacterial IRK protein AA sequences show no intron insertion, but in alignment with eukaryotic IRK AA sequences, they revealed six intrinsic gaps in their conserved and feature regions as the IRK sequences, showing that no eukaryotic equivalent sequences existed in the modern prokaryotes. However, it should be noted that either edge of the respective gap seemed to correspond at least to some of the intron insertion sites in the *Caenorhabditis *genomes in the five cases out of these six gaps (Fig. 6 and 7; purple background characters in the bacterial AA sequences).

### Comparison of AA sequences and genomic structures among 7 tunicate IRK genes and 108 vertebrate IRK genes

**Figure 8:**
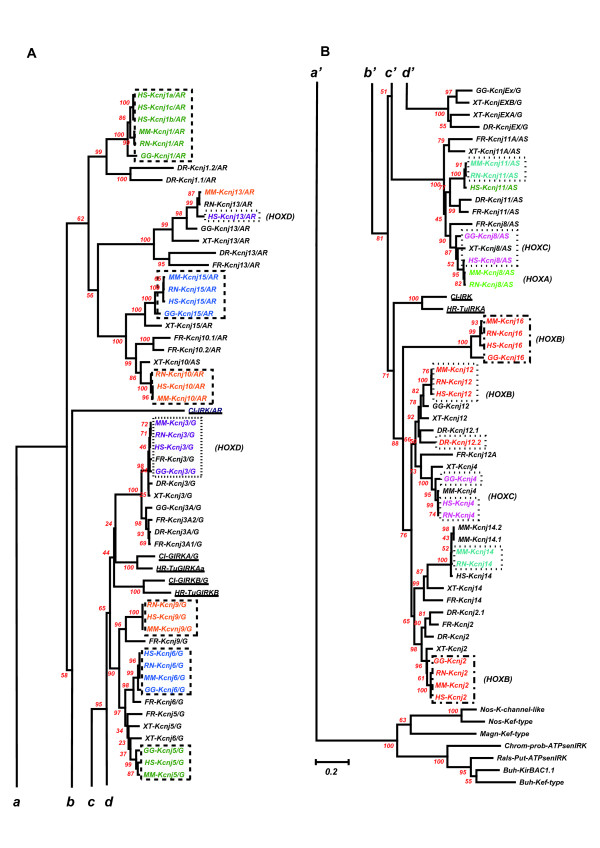
**The phylogenetic tree obtained from the alignment of a total of 7 and 108 IRK AA sequences of tunicates (underlined) and vertebrates, respectively**. (***A***) illustrates the upper part of the tree and (***B***) does the lower part. ***a ***and ***a ***, ***b ***and ***b'***, ***c ***and ***c'***, and ***d ***and ***d' ***indicate the continuation of branches from the upper part to the lower. The outgroup were seven bacterial IRK genes. Except for *Halocynthia *data, AA sequences are inferred from the established genome data bases, JGI, NCBI and GenBank, and Ensembl. Alignment was carried out by the ClustalX1.83 program. The gaps within nonpreserved regions were carefully deleted manually by the BioEdit program. The phylogenetic tree derived from the aligned data was then made by the Neighbor Joining Method in the Mega3 v3.1 program, using the Amino Poisson correction model with Gamma-distributed Rates among sites (Gamma parameter 2.0) and Bootstrap as the test of inferred branches (Repetition 500), including 270 sites with a pairwise deletion of Gaps/Missing Data. The **character **of the **same color **indicates the proximity of the chromosomal location of **gene pairs **between two subbranches. The closure by an interrupted line indicated that the chromosomal location between paired genes was very close in the order of 10 to 200 Kbp. The closure by dotted line indicates that linkage is weak. The Hox name in the parentheses indicates the equivalence of the chromosomal locations among different taxa by the presence of the specific Hox gene group. See also Additional file 5, chromosomal locations of genes. [see Additional file 5]

In Figure 8, the phylogenetic tree obtained from the alignment of a total of 115 IRK AA sequences of tunicates and vertebrates is illustrated. As shown above in the tree composed of human and tunicate sequences, the 3 major functional groups of IRK proteins are exactly represented in the major branches of the tree composed from 115 IRK sequences. And it was concluded that the 2 representative IRKs of tunicate GIRKs, TuGIRKAa and B, and the elementary IRK, TuIRKA, must be descendants of the ancestor forms of vertebrate GIRK and IRK, respectively. Although grouping the putative *Ciona *ATP-regulated IRK as a descendant of the ancestor sequence for ATP-regulated IRKs, including ROMKs, was not supported by a Bootstrap test, there were suggestive data to assign the common ancestor for all vertebrate IRKs to one of the relatives of the ATP-regulated types. From the survey of the genomic databases, a novel evolutional correlation was found between ATP-regulated IRK genes and G-protein-activated IRK genes, revealing the antitandem or palindromic duplication (Table 1).

**Table 1:** The palindromic duplication of the gene pair between ATP-regulated IRK and G-protein-activated IRK genes on a chromosome.

**Properties**	**Pair**	**Homolog**	**Summary of Expression **	**Hox-location**	**Chromosome**	**Chrom-location and direction**	**Distance between the pair in bp**
Genes in branches with green color figures in Fig. 5							
palindromic	GG-Kcnj1/AR	Kir1.1/ROM-K/ATP-regulated			GG-chromosome-24	1.06 Mb-reverse	31795
	GG-Kcnj5/G	GIRK4/Kir3.4/KATP-1		PKNOX2-24-0.14bM	GG-chromosome-24	1.09 Mb-forward	
palindromic	HS-Kcnj1/AR	Kir1.1/ROM-K/ATP-regulated	Kcnj1-Kidney epithelium		HS-chromosome-11	128.2 Mb-reverse	24045
	HS-Kcnj5/G	GIRK4/Kir3.4/KATP-1	Kcnj5-Heart muscle cell		HS-chromosome-11	128.3 Mb-forward	
palindromic	MM-Kcnj1.1/AR	Kir1.1/ROM-K/ATP-regulated			MM-chromosome-9	32.4 Mb-forward	80347
	MM-Kcnj5/G	GIRK4/Kir3.4/KATP-1			MM-chromosome-9	32.3 Mb-reverse	
palindromic	RN-Kcnj1/AR	Kir1.1/ROM-K/ATP-regulated			RN-chromosome-8	32.2 Mb-forward	22000
	RN-Kcnj5/G	GIRK4/Kir3.4/KATP-1	an annotated genomic sequence(NW_047799) using the gene prediction method: GNOMON*1		RN-chromosome-8	32.1Mb-reverse	

Genes in branches with orange color figures in Fig. 5							
palindromic	HS-Kcnj10/AR	Kir4.1/Kir1.2/ATP-regulated	Kcnj10-glia		HS-chromosome-1	157.2 Mb-reverse	11322
	HS-Kcnj9/G	GIRK3/Kir3.3	Kcnj9-neuronal cell		HS-chromosome-1	157.3 Mb-forward	
palindromic	MM-Kcnj10/AR	Kir4.1/ATP-regulated			MM-chromosome-1	172.4 Mb-forward	12138
	MM-Kcnj9/G	GIRK3/Kir3.3			MM-chromosome-1	172.4 Mb-reverse	
palindromic	RN-Kcnj10/AR	KIR4.1/Kir1.2/ATP-regulated			RN-chromosome-13	88.5 Mb-forward	24712
	RN-Kcnj9/G	GIRK3/Kir3.3/Kir3.1			RN-chromosome-13	88.5 Mb-reverse	

Genes in branches with blue color figures in Fig. 5							
palindromic	GG-Kcnj15/AR	Kir4.2/Kir1.3		HOXC8-1-87Mb(stand alone)	GG-chromosome-1	101.25 Mb-forward	275125
	GG-Kcnj6/G	GIRK2/Kir3.2/KATP-2		HOXC8-1-87Mb	GG-chromosome-1	100.9 Mb-reverse	
palindromic	HS-Kcnj15/AR	Kir4.2/Kir1.3	Kcnj15-kidney, epithelium, glia		HS-chromosome-21	38.5 Mb-forward	339968
	HS-Kcnj6/G	GIRK2/Kir3.2/KATP-2	Kcnj6-Neuronal cell		HS-chromosome-21	37.9 Mb-reverse	
palindromic	MM-Kcnj15/AR	Kir4.2/Kir1.3			MM-chromosome-16	95.9803Mb-forward	260226
	MM-Kcnj6/G	GIRK2/Kir3.2/KATP-2			MM-chromosome-16	95.5 Mb-reverse	
palindromic	RN-Kcnj15/AR	Kir4.2/Kir1.3			RN-chromosome-11	35.6 Mb-forward	460583
	RN-Kcnj6/G	GIRK2/Kir3.2/KATP-2			RN-chromosome-11	35.1 Mb-reverse	

Genes in branches with purple color figures in Fig. 5							
disperse	GG-Kcnj3/G	GIRK1/KIR3.1		HOXD(13)-7-17.1Mb-r	GG-chromosome-7	36.4 Mb-forward	
Palindromic?	HS-Kcnj13/AR	Kir7.1/ATP-related	Kcnj13-choroid plexus/thyroid/intestine	HOXD-2q31.1-176.8Mb-f	HS-chromosome-2	233.8 Mb-reverse	78000000
	HS-kcnj3/G	GIRK1/Kir3.1	Kcnj3-neuronal cell (heteromer with Kcnj6, 9)	HOXD-2q31.1-176.8Mb-f	HS-chromosome-2	155.8 Mb-forward	possibly paired with Kcnj13
			Kcnj3-heart muscle cell (heteromer with Kcnj5)				
disperse	MM-Kcnj3/G	GIRK1/Kir3.1		HOXD-2-45.0 cM-74.56Mb-f	MM-chromosome-2	55.4 Mb-forward	
disperse	RN-Kcnj3/G	GIRK1/KIR3.1		HOXD-3-57.3Mb-f	RN-chromosome-3	37.0 Mb-forward	

Genes in branches with red color figures in Fig. 5							
tandem	GG-Kcnj16	Kir5.1			GG-chromosome-18	7.95 Mb-Forward	14689
	GG-Kcnj2	Kir2.1			GG-chromosome-18	7.97 Mb-Forward	
tandem	HS-Kcnj16	Kir5.1	colocalize with Kcnj2 in brain and in kidney	HOXB-17q21-q22-44.06Mb-r	HS-chromosome-17	68.7 Mb-Forward	33932
	HS-Kcnj2	Kir2.1	Heart (Andersen Syndrome), Vas. Smooth muscle	HOXB-17q21-q22-44.06Mb-r	HS-chromosome-17	68.8 Mb-Forward	
tandem	MM-Kcnj16	Kir5.1		HOXB-11-56.08cM-95.87Mb-f	MM-chromosome-11	110.7 Mb-Forward	39324
	MM-Kcnj2	Kir2.1		HOXB-11-56.08cM-95.87Mb-f	MM-chromosome-11	110.7 Mb-Forward	
tandem	RN-Kcnj16	KIR5.1		HOXB-10-84.95Mb-f	RN-chromosome-10	100.5 Mb-Forward	59504
	RN-Kcnj2	Kir2.1/Kir2.2		HOXB-10-84.95Mb-f	RN-chromosome-10	100.6 Mb-Forward	
	DR-Kcnj2.1	Kir1.1/Kir3.1		Zebra-HOXB	DR-chromosome-3	2.0 Mb	

*1 Noted in NCBI genome project Rat. Predicted protein amino acid sequence was devoid of the major part of C-terminal distal to the feature sequence of Kcnjs. Therefore the AA sequence was not included in the construction of the phylogenetic tree in Fig. 5.

From the survey of the established genome databases, all gene pairs within IRK genes as far as thoroughly looked for are illustrated. In addition, the lower four pairs illustrate the tandem duplications of IRK genes in the elementary IRK group. Some of these tandem duplications in the human genome were reported previously [33]. Respective groups of genes which corresponded to the character colors in Fig. 8 are indicated.

On the tree derived from the AA sequences, the GIRK subbranch was distantly located from the subbranch of ATP-regulated IRKs. However, as illustrated in Table 1, the genome structures of respective IRKs in the one subbranch had their partners in the other subbranch, and each pair was located very closely together on the same chromosome in an antitandem or a palindromic way. For example, the 5' terminals of Kcnj 1 or ROMK1 genomes of human, mouse, rat, and chick were only 20 to 80 Kbp apart from the 5' terminals of Kcnj5 or GIRK2 genomes of respective species, and their 5' to 3' directions were just opposite to each other (green characters enclosed by interrupted lines in Fig. 8). There were two other similarly paired subbranches of Kcnj10 and Kcnj9 (orange characters) and of Kcnj15 and Kcnj6 (dark blue characters). The separation distances on the respective chromosomes were 10 to 20 Kbp for the former pairs and 260 to 450 Kbp for the latter. This type of pairing could be expected to apply also to the remaining subbranches of Kcnj 3 and Kcnj13. The genomes of the Kcnj 3 subbranch were all located on the equivalent chromosomes, where HoxDs were located in human, mouse, rat, and chick genomes, but only human Kcnj13 gene was located on the same chromosome as that of HoxD, with a relatively large separation distance from the human Kcnj3 genome.

These results suggested that both ancestors of GIRK and ATP-regulated IRK genes were antitandemly or palindromically aligned duplicates of each other possibly on the chromosome of ancestor chordates. After the event, two round duplications of chromosome numbers in the ancestor vertebrates [16] made approximately 4 times the members of genes in each major branch in the vertebrate clade, as suggested in the case of HOX genes [14]. As also suggested in the case of HOX genes, there may be some extra chromosome duplications in teleost fish or amphibians [17], because we could find some extra members of the IRK genes of puffer fish, zebra fish, and *Xenopus *in the respective subbranches. A recently reported *Ciona *genomic database from Ensembl suggested that the *Ciona *ATP-regulated IRK gene (Protein, ENSCINP00000010507; gene, ENSCING00000005094) is located at Chromosome 4q-373, but there was no information yet on the location of the *Ciona *G-protein-activated IRK gene (JGI; ci0100138554). The functional significance for the preservation of the proximity of paired IRK genomes on the vertebrate chromosomes will be discussed later.

There was also evidence of genuine tandem duplication within the branch of the elementary IRK gene group, as shown in the paired Kcnj2 and Kcnj16 subbranches (Fig. 8; Table 1). In this case, the directions from the 5' terminal to the 3' terminal were the same within respective pairs. This tandem duplication in the mouse IRK genome was noted previously [33], and this pairing was considered to be of an origin different from the above antitandem pairings. No other evidence of pairing was found in the elementary IRK gene group, except that a possible weak linkage between Kcnj12 and Kcnj2 subbranches was found as listed chromosomal location in Additional file 5 [see Additional file 5].

### A uniquely conserved intron insertion site among the vertebrate GIRK gene group and tunicate GIRK genes

**Figure 9:**
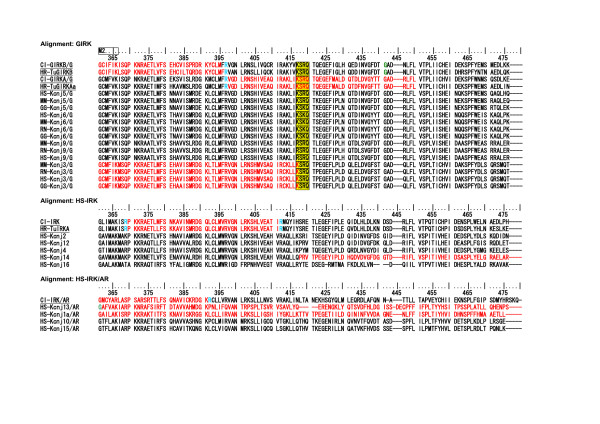
**The selected AA sequences from the alignment of 115 AA sequences to illustrate an exclusively conserved intron intervening site among the tunicate and vertebrate GIRK group**. The **yellow background and boxed **regions illustrate exclusively conserved AA sites among the tunicate and vertebrate GIRK group and the suggested interaction sites with G-protein beta and gamma[35]. Intron-intervening sites are illustrated by changes in the colors of AA code characters. A **red **to **black **transition or vice versa represents phase 0 intron insertion. **Green **and **sky blue **indicate phase 1 intron and phase 2 intron insertions, respectively.

**Figure 10:**
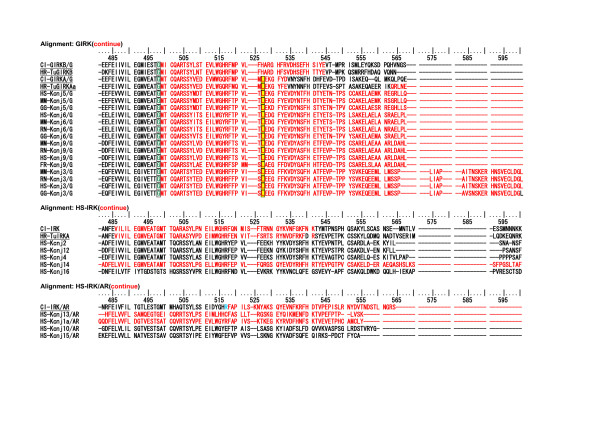
**The selected AA sequences from the alignment of 115 AA sequences to illustrate an exclusively conserved intron intervening site among the tunicate and vertebrate GIRK group (continued from Figure 9)**. The **yellow background and boxed **regions illustrate exclusively conserved AA sites among the tunicate and vertebrate GIRK group. Especially **glycine **colored by **green **indicates the existence of phase 1 intron. In the other group, the intron intervening site was not conserved in spite of the conserved amino acid residue in some genes. The alignments for the rest parts of AA sequences, which are not included in Fig. 9 and 10, are illustrated in an additional file [see Additional file 7].

**Figure 11:**
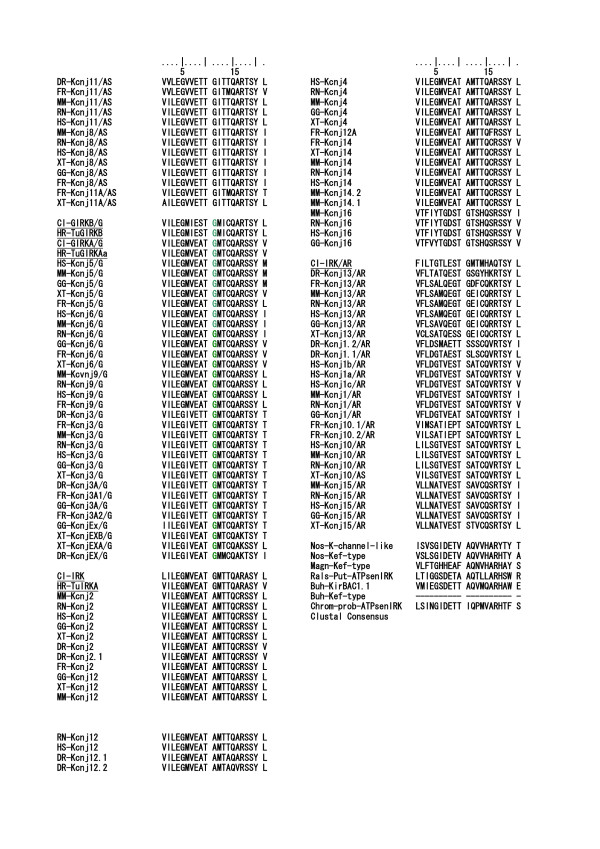
**The short region of the alignment of 115 AA sequences illustrates an exclusively conserved intron insertion site among the tunicate and vertebrate GIRK group**. Alignment: HR-115IRK-GMVEATGM The region corresponded to 21 amino acids from V303 and M323 in the C-terminal domain of human Kcnj5 (GIRK4). **Glycine **colored by **green **indicates the intron insertion site between the first and second nucleotides in the codon (phase 1 intron). In other group, the insertion site was not conserved in spite of the conserved amino acid residue in some genes.

**Figure 12:**
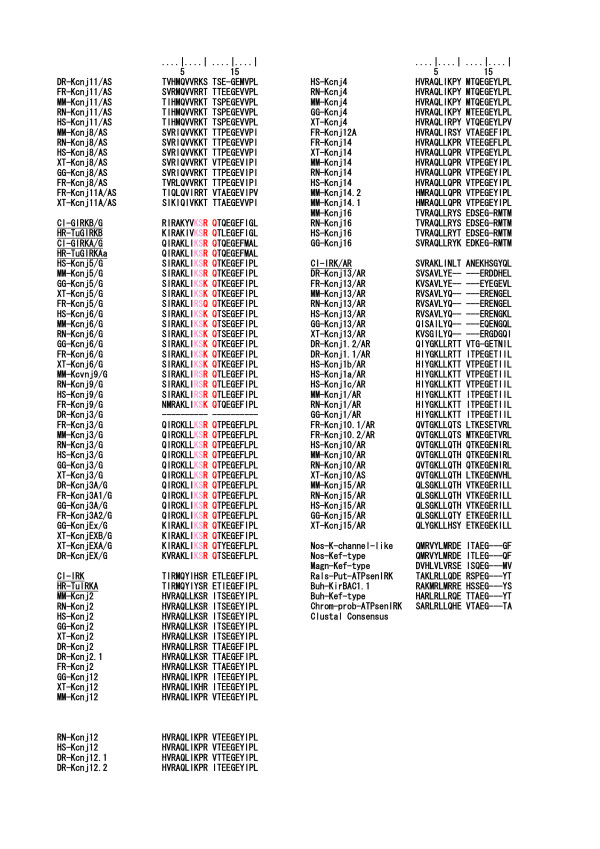
**The short region of the alignment of 115 AA sequences to illustrate exclusively conserved AA sites among the tunicate and vertebrate GIRK group**. Alignment: HR-115IRK-KSRQ. The region corresponded to 20 amino acid residues from S233 and L252 in the C-terminal domain of human Kcnj5 (GIRK4). Red-colored characters indicate strict and exclusive conservation, and the pink ones less strict.

**Figure 13:**
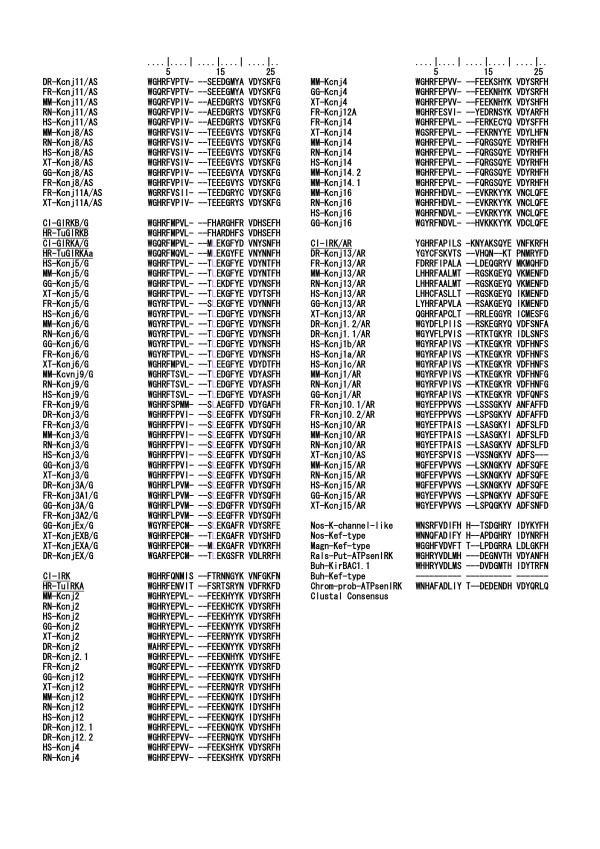
**The short region of the alignment of 115 AA sequences to illustrate exclusively conserved AA sites among the tunicate and vertebrate GIRK group**. Alignment: HR-115IRK-LXXG. The region corresponded to 24 amino acid residues from W329 and H352 in the C-terminal domain of human Kcnj5 (GIRK4). Purple-colored characters, L339 in human Kcnj5, indicate strict and exclusive conservation, except tunicate GIRKBs. The Leucine residue in mouse Kcnj6 (GIRK2) has been suggested to be the most effective previous activation sites by G-protein beta and gamma [35].

As described above, intron insertions in the vertebrate IRK coding regions are very few in comparison with those of the tunicate IRKs. Thus the intron and exon structures of the vertebrate IRK genes were quite different from those of the tunicates, though the AA sequences are phylogenetically closely related to their tunicate orthologs, as shown in the tree in Fig. 8. However, in the group of GIRK genes there is only one uniquely conserved intron insertion site among all vertebrate GIRKs and tunicate GIRKs in Fig. 9 and Fig. 10 [see Additional file 7]. As shown Fig. 11, this site was between the first and second nucleotide in the glycine codon and was preserved exclusively in the group of vertebrate and tunicate GIRKs. The site corresponded to G307 residue in the mouse GIRK1 or Kcnj3 and was located at the loop region near the hydrogen bonded turn a304–306 and between two beta strands a294–303 and a309. The site was suggested to be in the hinge region in the cytoplasmic C-terminal domain, which could be important for open-close transition by G-protein beta and gamma binding, and the residue seemed to be exposed to the cytoplasmic surface, as inferred from the mouse GIRK1 cytoplasmic crystal structure reported by Nishida and MacKinnon[34]. It is also noted that two exclusively conserved AA sequence regions in the C-terminal domain of the GIRK were located in the upper stream and downstream within 75 and 25 AA residue distances, respectively, from this intron insertion site and. The former, a234–237, were included in the beta-strand region a223–237 and seemed to be located on the cytoplasmic surface(Fig. 12). The latter, L333, was previously suggested to be the most effective activation site by G-protein beta and gamma (Fig. 13) [35]. The above-described conserved three regions seemed to be all located on the probable G-protein binding surface, making the solid triangle of molecular flame for the binding surface. The conserved intron insertion site, G307, seemed to be an especially important hinge region by which the working angle could be used to adapt to the global configuration of the binding surface.

### Phylogenic analysis and comparison of genomic structures of G-protein beta(GNB)s

**Figure 14:**
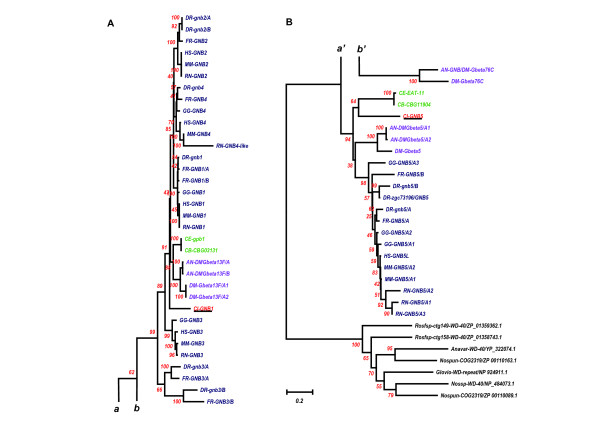
**The phylogenetic tree derived from 54 G-protein beta AA sequences**. (***A***) illustrates the upper part of the tree and (***B***) does the lower part. ***a ***and ***a ***, and ***b ***and ***b' ***indicate the continuation of branches from the upper part to the lower. A total of 54 AA sequences of eukaryotic G-protein beta and two beta-like AA sequences were analyzed [see Additional file 8]. The outgroup was 7 bacterial WD-repeat protein AA sequences. Tunicate GNBs were underlined. AA sequences are inferred from the established genome data bases, JGI, NCBI genomes, and Ensembl. Alignment was carried out by the ClustalX 1.83 program. The gaps within nonpreserved regions were carefully deleted manually by the BioEdit program. The phylogenetic tree derived from the aligned data was then made by the Neighbor Joining Method in the Mega3 v3.1 program, using the Amino Poisson correction model with Gamma-distributed Rates among sites (Gamma parameter 2.0) and Bootstrap as the test of inferred branches (Repetition 500), including 471 sites with a pairwise deletion of Gaps/Missing Data. *Drosophila *and *Anopheles *Gbeta76C genes may be differently grouped from the vertebrate-type GNBs according to this phylogenetic tree. Those bacterial AA sequences of WD-repeat proteins were also referred from the NCBI microbial genome database. Abbreviations: GNB, G-protein beta. Rosfsp, *Roseiflexus sp*.; Anavar, *Anabaena variabilis*; Nossp, *Nostoc sp*.; Nospc, *Nostoc punctiforme*; Glovio, *Gloeobacter violaceus*.

Since the intron insertion site, which was supposed to be in the G-protein beta binding region, was exclusively conserved in the group of GIRK genes, we attempted to analyze the genomic structures of G-protein betas interacting with these GIRKs. The data were all obtained from the established genomic databases [see Additional file 8]. Figure 14 illustrates the phylogenetic tree derived from 54 G-protein beta AA sequences and 7 bacterial WD-repeat protein AA sequences as the outgroup. Because this case was different from the IRK sequences, two major groups of G-protein beta very likely existed within the ancestor sequences of both protostomic and deuterostomic clades, that is, the first group of GNB1 to 4 and the second group of GNB5. In the vertebrate clade after diversification from the ancestor chordate, the first group produced 4 members, and the number seemed to remain throughout the vertebrate evolution, except for some extra production in the teleost fish. Also in fly and mosquito, two major groups remained.

**Figure 15:**
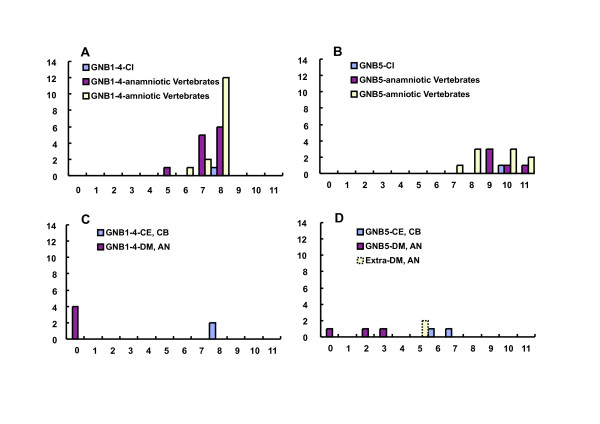
**Frequency histograms of intron numbers in all 52 GNB genes examined**. After we identified the G-protein beta AA sequences and obtaining the trees, we retrieved all respective genomic sequences from the databases. And the exon intron junctions, the length of introns, and the numbers of intron insertions within coding regions were inferred, and listed in Additional file 8 [see Additional file 8]. Figure ***A ***and ***B ***represents the histograms of intron insertion numbers within coding regions for two major groups of vertebrate GNBs, that is, GNB 1 to 4 and GNB 5 groups, respectively. Anamniotic vertebrates mean fish and amphibian, and amniotic birds and mammals. Abscissa, Intron numbers. Ordinate, and Number of genes, which showed the numbers of intron insertions in the coding regions as indicated on the abscissa. Figure C and D is the similar histogram for two major groups of GNBs genes of the protostomic clades, that is, *Caenorhabditis elegans *and *brigssae*, and *Drosophila melanogaster *and *Anopheles gambiae*. Extra means Gbeta76C genes of *Drosophila *and *Anopheles*.

**Figure 16:**
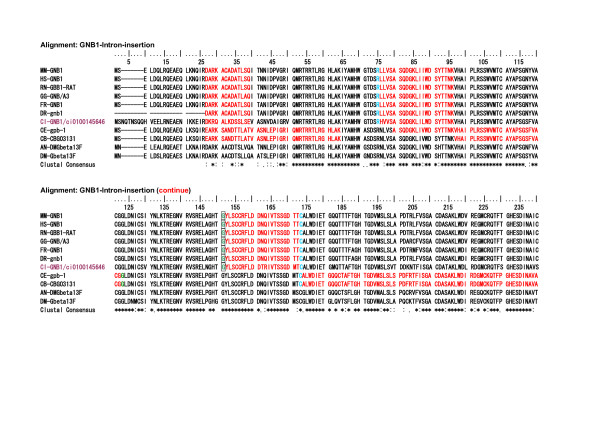
**Alignment of a tunicate, two *Caenorhabditis *and *Anopheles*, a *Drosophila*, and six vertebrate GNB1 AA sequences**. The portions of intron insertion sites are illustrated by changes in colors of AA code characters. Red to black transition or vise versa is the case for the intron insertion that occurred between two neighboring AA codes (phase 0 intron). Green and sky blue indicate phase 1 intron and phase 2 intron, respectively. The upper 7 taxis are deuterostomes, and the lower 4 are protostomes. Alignments were performed by the ClustalX 1.83 program. The lowest line indicates Clustal Consensus with stars and dots.

**Figure 17:**
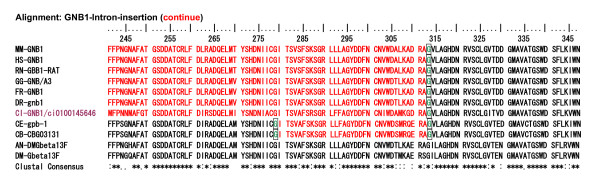
**Alignment of a tunicate, two *Caenorhabditis *and *Anopheles*, a *Drosophila*, and six vertebrate GNB1 AA sequences (continued from Figure 16)**. The alignments for the rest parts of AA sequences, which are not included in Fig. 16 and 17, are illustrated in an additional file [see Additional file 9]. Another alignment of a tunicate, two Caenorhabditis, two Anopheles, a Drosophila, and seven vertebrate GNB5 AA sequences is also included in the additional file [see Additional file 9].

In terms of intron-exon structures, the frequency histograms in Fig. 15 demonstrated that the 7 to 11 intron insertions in the coding region were rather constant among all vertebrate GNBs and *Ciona *GNBs. Two groups of GNBs in *Caenorhabditis *revealed 8 and 7 intron insertions, similar to those in *Ciona*, respectively. However, the GNBs in fly and mosquito revealed a much lower number of intron insertions than *Caenorhabditis *did. An alignment of GNB1 protein sequences illustrated not only strict amino acid residue conservation, but also strict intron insertion site preservation among human, mouse, rat, fish, *Ciona*, and *Caenorhabditis *GNB1 proteins (Fig. 16 and 17). However, fly and mosquito GNBs did not conserve the intron insertion sites. The alignment of GNB5 protein AA sequences illustrated similar results [see Additional file 9]. The physiological significance of intron insertion site conservation will be discussed later.

## Discussion

### Summary of results

We sequenced three *Halocynthia *IRK genomic genes and determined their genomic structures. Furthermore, a total of 131 IRK genomic genes were inferred from the established genomic databases. The phylogenic trees derived from the known or inferred AA sequences revealed the clear diversification of deuterostomic IRK from protostomic IRK, and the tunicate IRKs were possibly representatives of ancestor forms of three major groups of IRKs in the vertebrate. However, the genomic structures, including intron-exon connections of the tunicate IRKs, showed considerable similarities to those of *Caenorhabditis*. Although the major groups of tunicate IRK proteins included only one or two members, in the vertebrate clade the members in one major group increased at least four times. The generation of paralogues can be achieved by various types of gene duplication, such as tandem and/or chromosomal. However, the generation of the major groups in the tunicates and vertebrates may be of more ancient origin, and some of the mechanism may be derived from the unique palindromic duplication. Finally the strong conservation of intron insertion sites in the coding regions of vertebrate GIRK group indicated the close functional correlation between the genome structures and the protein structures.

### Intron early theory

The significance of intron insertion has been considerably notified by the claim of exon theory [8]. The exon theory persisted that the ancestral eukaryotic genes are generated from numerous short exons connected with ancient introns, and exon shuffling introduced new functions into the gene product proteins [23]. Provocative evidence for the theory is provided by the high-frequency insertion of introns between codon sequences, phase 0 intron, and the conservation of intron insertion sites in the coding regions of orthologous proteins from a phylogenetically wide range of taxis [23]. However, because of massive intron loss during the evolutionary process and the significant production of new intron insertion in the highly evolved taxis, such as human and *Drosophila*, the ancient intron insertion sites may be vastly lost in the genomes of present-day organisms [9]. Those situations have facilitated several types of late intron theory or synthetic theory of intron evolution [10-12].

In the present study, we purposefully limited observation and discussion to within the gene group of definitely functionally defined proteins, that is, IRK channels. Results indicated that both the tunicate IRK genes and the *Caenorhabditis *IRK genes conserved large numbers of intron insertion sites in the functionally important transmembrane and pore regions. Further, three insertion sites were commonly conserved in terms of AA sequences between the tunicates and the *Caenorhabditis*, indicating that some of the cornerstones of early evolutional history remained.

Although there is an intriguing hypothesis that the exon corresponds to protein domains [23,36], the conserved insertion sites seemed not always to exist at the borders of domains. Instead, they were at the structurally critical points, such as the membrane spanning region or inside the pore regions in the present study. This point will be further discussed below.

In both chordates and protostomal clades, when the respective members increased within functionally different major groups of IRK genes, the number of intron insertions abruptly decreased. In the tunicates or the *Caenorhabditis*, the members remained small in number possibly because of the size constraint of whole genomes, and the intron insertion sites were conserved. Our observation could suggest no points about intron insertions in bacterial genes; however, we were inclined to consider that the intron appearance is closely related to the early appearance of eukaryotic organisms, because some residual gaps of IRK amino acid sequences were left in the modern bacteria, roughly corresponding to the conserved intron insertion sites of the ancestral eukaryotes reflected in the *Caenorhabditis *or tunicate genomes. This suggested that the intron insertion mechanism newly appeared at least at the time of diversification between the eukaryotic and prokaryotic ancestors, or that the introns in the prokaryotic and eukaryotic common ancestor, the progenote, genomes disappeared in the prokaryotes, as suggested previously [23]. This consideration supported the exon theory in the evolution of the IRK gene family.

There remains the possibility that the common intron insertion between tunicates and Caenorhabditis could be derived from convergent evolution during later history way after eukaryotic diversification from prokaryotes. However, it was totally unclear what kinds of adaptive merits produced by the multiple intron insertion commonly existed in their phylogenetically different ancestors, although convergent evolution of the AA sequences might be derived from the functional similarity of IRK proteins [37].

### Gene duplication

In the vertebrate it has been proposed that gene diversification has been attained by various kinds of gene duplication [15,16,19,38]. There are many examples of tandem duplication, which could produce the paralogues of genes [39]. It could be inferred that one of the duplicated pair became free from the functional constraining and acquired the new function or degenerated as nonfunctional by the accumulation of mutations. It is generally understood that the duplication by random insertion could be derived from the genomic insertion of reverse transcript of the original gene by various transposon-like mechanisms and that the exact duplication of gene regulatory sequence, such as promoters and enhancers, are rather difficult. Thus block duplication or chromosomal duplication of the concerned gene groups should be an easier way to produce new functional paralogues. The famous two-round duplication of the chromosome set has been proposed by Ohno in the ancestor vertebrate [13], and the theory has strong support by the existence of four sets of tandemly aligned Hox gene groups in the amniotic vertebrate genomes and the recent analysis on the whole genome sequences derived from a wide-range of vertebrate taxa including both anamniotes and amniotes [14-16]. The presence of extra sets of Hox gene groups in fish has been explained by the extra duplication of chromosomal sets in the teleosts[17].

In the present study, two major gene groups, ATP-regulated IRK and G-protein-activated IRK, of which gene branches were placed quite distantly on the phylogenetic tree derived from the IRK AA sequences, were found to be physically correlated closely and franked anti-tandemly as pairs on the individual chromosomes. This is so-called palindromic duplication, which has recently attracted much attention in the adaptive gene expansion of carcinoma stem cell or fungi, Saccharomyces, genomes [18-20,38]. The unique palindromic duplication during evolutional time found in the present study would forcibly make the gene pair share the gene regulatory regions. Although the promoters may be separately placed, the coincidental transcriptional activity should be difficult between the gene pair. The expression must be mutually exclusive. Actually, G-protein-activated IRKs are exclusively expressed in the neuronal cells of the nervous tissues and heart cells [40], and the paired ATP-regulated IRKs are expressed in the epithelial cells and the glia of the nervous tissue [41-45].

This rather unique type of palindromic duplication must have been originated before the subbranching in respective major groups of genes, that is, at the time of the ancestors common to the tunicate and the vertebrates. Then the parallel but independent functional evolution during long archeological history could occur in the gene pairs deriving, respectively, two major distinct gene groups as a result of mutual and physical constraining in the gene regulatory regions. Therefore the palindromic duplication was highly expected to exist also in the modern tunicate genome. However, no evidence of this type duplication of IRK genes has remained in the genome though the physical maps of the tunicate chromosomes have not yet been completed as described in the Results section. In the Ascidia, the tandem arrangement of Hox genes was dispersed, as previously reported [46,47]. Similarly, it was possible that the evolutional evidence of the antitandem or palindromic duplication of the IRK genes may disappear because of the gene dispersion in the Ascidia during the evolutionary time. As Ferrier and Holland [47] have suggested, the dispersion of the Hox gene groups may be deduced from the inference that the temporal colinearity as well as constraint on the cluster organization might have been removed from animals that undergo rapid embryogenesis with a low cell number and predominantly mosaic development. However, it may also be plausible that the dispersion in the genome itself could lead the rapid embryogenesis with a low cell number and predominantly mosaic development, resulting from the removal of the spatial and temporal colinearity in the genomic structure and regulation, without which the animals had to become adaptable.

Two rather marginal but important points could be discussed in relation to the palindromic duplication. First, according to the constructed phylogenetic tree the G-protein-activated IRK gene may be diverged from the ATP-regulated IRK gene. After the palindromic duplication of ancestoral ATP-regulated IRK genes, the G-protein binding segment on the duplicated genes could be forced to evolve in parallel with G-protein beta and gamma genes. On the other hand, the coupled evolution between G-protein-activated and ATP-regulated IRKs as a result of the possible joint ownership of the gene regulatory regions must be related to apparently independent but actually interdependent functions of the epithelial system derived from mesenchymal or endodermal sheet and the tubular nervous system from ectodermal sheet in the common ancestor of the tunicates and vertebrates. Ionic balance, especially potassium, resulting from the IRK function in the epithelial transport systems and neural excitable epithelium must have been important adaptive factors to coordinate the regulations of K ion transport activities of kidney and neurons in the freshwater or landed vertebrate evolution to balance their effects on the internal milieu and membrane excitability. For example, the high activity of ROMK in the kidney, one of the members of ATP-regulated IRK, will produce low K^+ ^concentration in the internal milieu, and, if occurring simultaneously, the high activity of GIRKs in neurons or heart cells will be extremely inhibitory for membrane excitability. In the above respect, it was interesting that the seawater living tunicates have a dispersed location between the ATP-regulated and G-protein activated IRK genes, where the ionic internal milieu is balanced mainly by the seawater composition. In this discussion, it was important to notice that the ATP-sensitive or SUR-coupled IRKs, such as Kcnj8 and Kcnj11, were evolutionally different from ATP-regulated IRKs, such as ROMK (see Fig. 5), and that ATP-sensitive IRK genes were not paired with G-protein-activated IRK genes. Actually ATP-sensitive IRK did coexist with GIRKs in the heart cells and neurons.

Second, by closely looking at the phylogenetic tree, we see that the branching in the respective subbranches was not exactly parallel between the two paired major IRK gene branches, as shown in Fig. 8. The exchange of partners resulting from one round of homologous recombination between the two members of gene pairs within four members during the two-round chromosomal duplications nicely explained the slight difference of the subbranching pattern between two major branches of ATP-regulated and G-protein-activated IRK groups.

### Functional significance of intron insertion

Most introns in the IRK genomes disappeared in the vertebrate genomes after gene diversification. However, unique intron insertion sites remained precisely conserved in respect to both AA code and intracodon insertion sites exclusively in the G-protein-activated IRK gene group, including the tunicate ones. As AA sequences, the conservation of the site was partially conceived in the genes of other major groups. The site is the hinge region that is important for the opening-closing of the IRK channel correlated with G-protein beta and gamma binding, as described in the Results. The exclusively conserved Glycine, which was also the conserved intron insertion site, composed the hinge region that must bend or rotate as the pivotal role in adjusting against the G-protein beta and gamma binding and accordingly to change the open or closed state of channels. Thus the strongest evolutionary conservative pressure must be applied to the hinge site. Here it could be inferred that the conservation of intron insertion with phase 1 but not phase 0 or 2, which has a redundancy at the 5' or 3' side of introns, was probably the most plausible mechanism to result in an all-or-none or alternative mutation, that is, conservation or fetal. This is because any addition, deletion, or point mutation in the neighborhood of the insertion site would produce mostly a disruption of intron insertions, or even in rare cases a dysfunctional change in an amino acid residue, even if the intron insertions were kept. Thus it could be easily imagined that both sides of the conserved phase 1 intron insertion site could be confronted by the high evolutional conservative pressure or strict parallel evolution to be adaptable for binding to the partner proteins.

In the above, it was interesting that our survey on the genome structures of G-protein betas (GNBs), of which GNB1 is especially the well-known partner of GIRKs and also other many G-protein activated proteins [48-50], revealed the extremely conserved intron insertion sites from the tunicate genes to the human genes in the vertebrate clade. Among the GNB genes, the vertebrate evolution seemed to introduce the functional diversification of only marginal activity by the member increase because of whole genome duplication in the specialized groups on the phylogenic tree. It was suggested instead that because of the multi-interactive nature of the protein, the high conservative pressure on the pivotal sites in their molecular structure made their intron insertion sites, including AA codes, constant during the evolutional time. Actually, the conserved sites of phase 1 intron insertion are placed on the hinge regions between two beta sheets or helix structures, as inferred from rat GNB1 crystal structures reported [51,52]. Thus it could be inferred that the site conservation of intron insertion, especially phase 1 intron, is one of the representatives of the genome structures constrained by the protein structures for the adaptation to the protein-protein interaction. On the other hand, it has been well discussed that phase 0 or phase 2 introns have a significant role in exon shuffling or domain exchange for protein structural evolution [23].

### Structure and evolutionary rate

**Figure 18:**
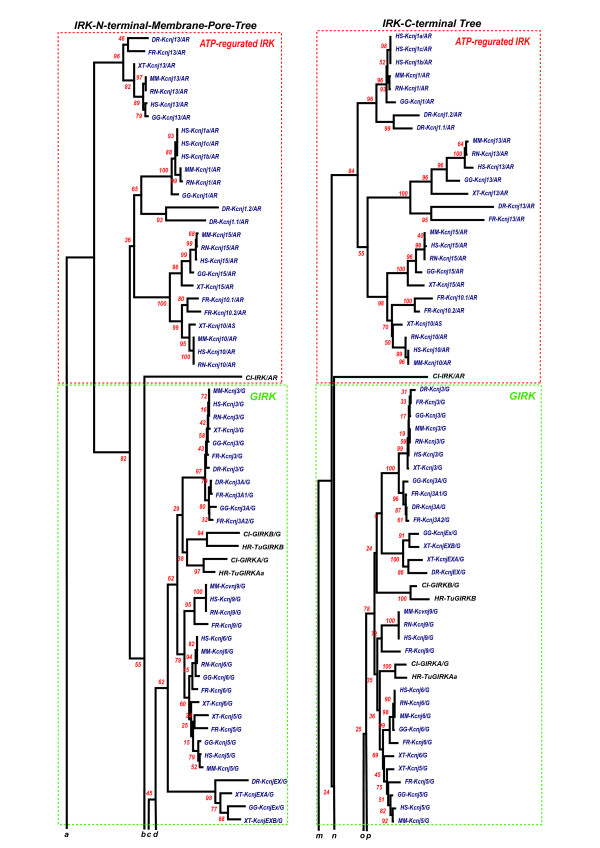
**Two separate phylogenetic trees for the IRK N terminal plus membrane-spanning region and for the IRK C terminal region**. This figure illustrates only the upper halves of the trees and the following figure 19 does the lower halves. ***a, b, c ***and ***d ***, and ***m, n, o ***and ***p ***indicate the continuation of branches from the upper halves in this figure to the lower halves in the following figure. Two sets of AA sequence alignments are obtained from two sets of AA sequences divided at the 3' terminal of respective M2 regions in a total of 7 (underlined) and 108 IRK AA sequences of tunicates and vertebrates, inferred from the established genome databases, JGI, GenBank, and Ensembl, except *Halocynthia *data, the same data as in Fig. 5. In this figure the 270 sites used in the tree of Fig. 5 were divided into 123 sites from the membrane and pore regions and into 147 sites from the C-terminal region. This region was defined as from the 3' end of the M2 region to the end. Both trees for these two regions were made by the Mega3 v3.1 program, as described in the legend of Fig. 5, that is, phylogenetic trees being constructed by the Neighbor-joining methods of Mega 3 v3.1 with the Bootstrap test (500 repetition) and with the Gamma distance model of alpha parameter 2.0. The outgroups were two sets of seven bacterial IRK genes similarly divided at the 3' terminal of respective M2 regions. It was concluded that the C-terminal region of the G-protein-activated IRK group (a total 147 AA sites) revealed a rather slower evolutionary rate or lower mutational rate than that of the ATP-regulated IRK group, except for the Kcnj1 subgroup, and the N-terminal and membrane-spanning region (123 AA sites) of the ATP-regulated IRKs revealed slower evolutionary rates than the same region of the G-protein-activated IRK group.

**Figure 19:**
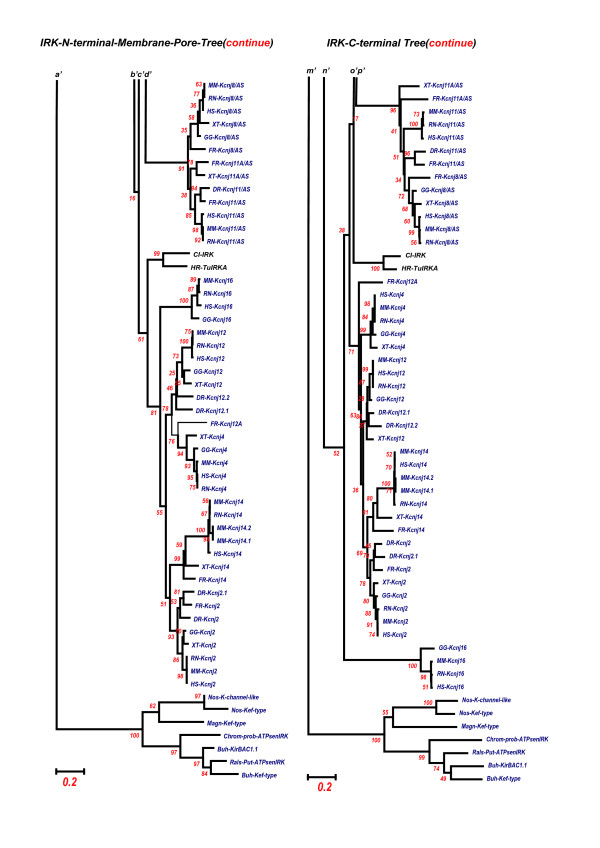
**Two separate phylogenetic trees for the IRK N terminal plus membrane-spanning region and for the IRK C terminal region**. This figure illustrates only the lower halves of the trees and the preceding figure 18 does the upper halves. ***a', b', c' ***and ***d' ***, and ***m', n', o' ***and ***p' ***indicate the continuation of branches from the upper halves in the preceding figure 18 to the lower halves in this figure.

It has been hotly debated that protein structural constraints because of protein-protein interaction could introduce a slow evolutionary rate in AA sequences of the contacting regions of both proteins, because of the necessity of the preservation of functionally significant structures and of the parallel evolution between the paired interacting regions [53-55]. This inference was also reasonably conceived in the C-terminal regions of the IRKs in comparison with the N-terminal and membrane region of the IRKs. As shown in Fig. 18 and 19, two separate phylogenetic trees for the IRK N terminal and membrane-spanning region and for the IRK C terminal region illustrate that the C-terminal region of G-protein activated IRK group revealed rather slower evolutionary rates or lower mutational rates than those of ATP-regulated IRK group except the Kcnj1 subgroup. The N-terminal and membrane-spanning regions of the ATP-regulated IRKs revealed slower evolutionary rates than those of the GIRK. This may be derived from the protein-protein or protein-ligand interactions occurring in the intramolecularly different regions between the GIRKs and the ATP-regulated IRKs. The exceptional Kcnj1 subgroup, which revealed a slower rate in the C-terminal region, has been known to have the ATP/PIP2-binding site in a 39-amino-acid region of the C-terminal [56]. Thus it was suggested that the structural constraints of the proteins limit the evolutionary rates of the particular protein regions. The conservation of intron-insertion sites at the functionally significant regions described above could be one of the important mechanisms to facilitate this general tendency.

## Conclusion

In the present study, we attempted to analyze the genomic structures of a group of proteins, which are functionally well defined, and to compare them among a phylogenetically wide range from protostomal to deuterostomal clades. We aimed to elucidate the functional significance in evolutional changes in the genome structure, such as intron insertion and gene duplication, and the interactive relation between the expressed protein structure and the genomic structural changes. The results follow. First, the general inron-exon structures may be derived from the early time of eukaryotic evolution, when the protein family revealed the first and elemental function with their unique molecular structures. Second, the various types of gene duplication could contribute to the gene diversification resulting in the functional evolution, but some constraints in the genomic structures because the unique molecular mechanisms of the duplication, such as palindromic duplication, may produce the adaptive parallel or coordinate evolution among the protein family members between organs or tissues that were functioning differently. Third, the conserved intron insertion site, especially in the case of phase 1 intron, could be correlated with amino acid residues that were structurally pivotal roles in the protein-protein interaction. Thus it was suggested that the ancient intron insertion sites, which are intrinsically related to the functionally key region of the proteins, in the eukaryotic genome, or possibly in the prokaryotic and eukaryotic common ancestor, and the unique palindromic genomic duplication in the genomes of vertebrate clade shaped an elementally functioning membrane protein family during evolution.

## Methods

### Halocynthia roretzi (HR) IRK cDNA

The cDNAs, TuIRKA, TuGIRKAa, and TuGIRKB were gifts from Dr. Y. Kubo in the Division of Biophysics and Neurobiology, Department of Molecular Physiology, National Institute for Physiological Sciences, and were subcloned into pBluescript II SK2 (Stratagene, San Diego, CA) in accordance with the manufacturer's protocol and sequenced [see Additional file 2].

### Genomic library screening

A *Halocynthia roretzi *genomic library constructed in λEMBL3/BamH I Vector (Stratagene) was a gift from Dr. H. Okado of the Department of Molecular Physiology, Function Research Division, Tokyo Metropolitan Institute for Neurosciences. This library was screened using specific EcoRI fragments of respective HR IRK cDNAs as probes. Filters were prehybridized in a solution containing 50% formamide, 53 SSC, 53 Denhardt's solution, and 0.1% SDS for 1 h at 42°C, then hybridized overnight at 42°C in the same solution containing Digoxigenin-labeled probes. The DNA probes were random primed, labeled with Digoxigenin-11-dUTP using DIG-High Prime, a 5 × concentrated labeling mixture of random hexamers, dNTP mix containing alkali-labile Digoxigenin-11-dUTP, and labeling grade Klenow enzyme, and optimized according to the manufacturer's manual (Roche; DIG-High Prime DNA Labeling and Detection Starter Kit II). The filters were washed twice in 23 SSC and 0.05% SDS at room temperature for 20 min and once in 13 SSC and 0.1% SDS at 55°C for 2 h. They were then treated with chemiluminescence reaction buffer (Roche; DIG-High Prime DNA Labeling and Detection Starter Kit II). Positive clones were detected by exposing the luminescence to X-ray films contacted with the filters. The λEmble3 clones containing these positive inserts were amplified and used for the genomic sequencing. The obtained clones were 2 for the TuIRKA gene, the TuIRKA11 clone, and the TuIRKA14 clone, 2 for the TuGIRKAa gene, the TuGIRKAa3 clone, and the TuGIRKAa9 clone, and 1 for the TuGIRKB gene and the TuGIRKB2 clone, showing the possible coverage of all ORFs, 5' UTRs, and 3' UTRs with restriction maps [see Additional file 3]. And later the coverage was all confirmed with the sequencing. These 5 clones corresponding to respective cDNAs were digested with Xho I and Sal I, and the digests were subcloned into pBluescript II SK2 using an Xho I or Sal I restriction site, respectively. For the TuIRKA gene, the obtained subclones were 17 from the first λEMBLE3 clone and the TuIRKA11 clone and 3 from the second λEMBLE3 clone and the TuIRKA14 clone. For the TuGIRKAa gene, 7 subclones from the TuGIRKAa3 clone and 3 from the TuGIRKAa9 clone were obtained. For TuGIRKB, the subclone was recloned into pBluescript II SK2 from the TuGIRKB2 clone itself. All subclones were sequenced with appropriate primer combinations at least in both directions, and in some cases a third sequencing was carried out. In the case of the TuGIRKA gene, PCR clonings of the two gap regions between subclones were performed on the original λEMBLE3 clones, using part of the decided flanking sequences as primers, and also subcloned into pBluescript II SK2 and sequenced. All suspected boundaries between the neighboring subclones were subjected to perform PCR on the original λEMBLE3 clones, using appropriate primers for the tagged sequence derived from the sequenced regions placed at both ends of the boundaries. And the continuation beyond the boundaries of subcloned fragments were all confirmed.

### DNA sequencing

DNA was sequenced by the Dye Terminator method, using an automated laser fluorescent DNA sequencer (ABI PRISM model 3100, Applied Biosystems, Foster city CA, USA) and a BigDye Terminator Cycle Sequencing Kit (Applied Biosystems, Foster city CA, USA). DNA sequences were analyzed by using the computer program DNASIS3.2-MAC (Hitachi Software, Tokyo, Japan). Exon-intron boundaries were identified by comparing the genomic and cDNA sequences. All obtained genomic and cDNA sequences necessary for the present experimental conclusions are listed in Additional files 1, 2, 3 and 4 [see Additional files 1, 2, 3 and 4].

### Retrieving sequences from the C. intestinalis genome and a cDNA/EST database

The *C. intestinalis *IRK channel protein sequences were tBLASTn searched against the draft or completed genome sequence (Ref.: [4], and Ensembl and DOE Joint Genome Institute [JGI] *Ciona intestinalis *v2.0 [JGI site for the complete *C. intestinalis *genome sequence and gene annotations]: [57]) and a cDNA/EST database (Ref.: [7], and Ghost Database: [58]) using human and HR IRK channel protein AA sequences [29,30]. IRK channel proteins were identified by using the following basic method. Briefly, when the corresponding cDNA sequence covering the diagnostic sequences for an IRK channel molecule, such as a channel pore region and two franking transmembrane regions, and available by InterPro search [59], the deduced protein sequence was used for the analyses. When the cDNA sequence was not available and grail EXP or genewise confidently predicted the gene encompassing the entire channel region, the peptide sequence deduced from the gene model was used. When the predicted gene model was not perfect, but the ESTs covered either the entire region or the region lacking the gene model, the peptide sequence was deduced from the assembled sequence obtained by using either a set of ESTs (5' and 3' EST pair), multiple sets of ESTs, or both an EST and the gene model. After the protein AA sequence was identified, the respective genomic sequences were retrieved from databases. All analyzed Ciona IRK genomic genes and other genes are listed in Additional file 5 [see Additional file 5].

### Molecular phylogenetic analysis

The identified or predicted IRK channel protein sequences from human (database version: NCBI Build 36.2), mouse (database version: NCBI Build 36.1), *Takifugu rubripes *(database version: IMCB/JGI FUGU 4.0), zebra fish (*Danio rerio*, database version: Ensembl Zv 6), rat (*Rattus norvegicus*, database version: RGSC v3.4), chicken (*Gallus gallus*, database version: WASHUC 1), and frog (*Xenopus tropicalis*, database version: JGI 4.1) were retrieved from NCBI Genomes [60] and/or Ensembl [61]. Diagnostic sequences for each retrieved IRK channel molecule, such as a channel pore region and two franking transmembrane regions, were identified by InterPro search [59]. To delineate vertebrate gene families, a similarity search was performed (tBLASTn, [62]; E-value cutoff E-10) with all IRK channel proteins from the organisms listed in Additional file 5 [see Additional file 5], that is, the proteins of HR and *Ciona intestinalis *(database version JGI2.0), *Drosophila melanogaster *(database version BDGP 4), *Caenorhabditis elegans *(database version WS 150), *Anopheles gambiae *(database version AgamP3), and seven prokaryotic IRK channel proteins that were added as the outgroup species (NCBI Genomes Prokaryotic Projects). The prokaryotic sequence was used to root the phylogenetic tree. Redundancy between the families was removed. The sequences were aligned by using the ClustalX 1.83 program [63,64]. Alignments in nonconserved regions of channel proteins were carefully checked by eye, and regions with ambiguous alignments, in particular at the less-conserved cytoplasmic region and extracellular region, were eliminated. Thus verified alignments were used to construct phylogenetic trees. Neighboring joining trees (with 500 bootstrap replicates) were constructed using MEGA3 [65,66] by using a substitution model of Amino: Poisson correction with Gamma Distributed Rates among sites (Gamma Parameter 2.0) and including sites with a pairwise deletion of Gaps/Missing data. For the analyses shown in Figs. 3, 4, 8, 18 and 19 when the NJ trees were constructed, branch points were considered significant only when the bootstrap tests gave a significant value >50%. The sequences used are designated in succession by the abbreviation of the species and the gene name. Abbreviations of the species are as follows: HR for *Halocynthia roretzi*, CI for *Ciona intestinalis*, HS for human, MM for *M. musculus*, RN for *Rattus norvegicus*, GG for *Gallus gallus*, DR for *Danio rerio*, FR for *Takifugu rubripes*, XT for *Xenopus tropicalis*, DM for *D. melanogaster*, AG for *Anopheles gambiae*, CE for *C. elegans*, and CB for *C. briggsae*. After the protein AA sequences were identified and the trees obtained, all respective genomic sequences were retrieved from the databases. And the inferred sites of exon intron junctions and the length of respective introns were used for a comparison of genomic structures among the organisms examined.

### Relative dating of duplication events

The presence of various palindromic gene duplication events was systematically analyzed on the Phylogenetic trees with standard distance metrics obtained above. Duplication events were evaluated by relative dating and thus were based on the relative position of the duplicated genes compared to speciation events in the phylogenetic tree.

### Retrieving G-protein beta (GNB) AA sequences and their genome structures from the genome databases

A total of 54 AA sequences of eukaryotic G-protein beta and two beta-like AA sequences were inferred from genome databases, NCBI genome projects, and Ensembl. To delineate GNB gene families, a similarity search was performed by using human G-protein beta1 and beta5 AA sequences (tBLASTn, [62]; E-value cutoff E-10), and all GNB AA sequences retrieved from both protostomal and deuterostomal clades were listed in Additional file 8 [see Additional file 8]. Moreover, seven bacterial WD-repeat protein AA sequences were retrieved from the NCBI prokaryotic genome data projects and were used as the outgroup for the construction of GNB trees. After the G-protein beta AA sequences were identified and the trees obtained, all respective genomic sequences were also retrieved from the databases. And the exon intron junctions, length of introns, and numbers of intron insertions within coding regions were inferred, as listed in Additional file 8 [see Additional file 8]. Tunicate GNB data were obtained from the JGI database, *Ciona intestinalis *v2.0. Alignment was carried out by the ClustalX 1.83 program [see Additional file 9]. The phylogenetic tree derived from the aligned data was made by the Neighbor Joining Method in the Mega3 v3.1 program, using the Amino Poisson correction model with Gamma-distributed rates among sites.

## Supplementary Material

Additional file 1 **TuIRKA, TuGIRKAa and TuGIRKB genomic sequences**. The data provided represent the determined IRK gene genomic sequences. Click here for file

Additional file 2 **TuIRKA, TuGIRKAa and TuGIRKB cDNA sequences**. The data provided represent the IRK cDNA sequences inserted in the vectors used in the present experiment. Click here for file

Additional file 3 **Scematic illustration of TuIRKA, TuGIRKAa and TuGIRKB genomic sequences**. The data provided the schematic illustration of the intron-exon structures of *Halocynthia *IRK gene genomic sequences. And the obtained genomic clones used for sequencing. Click here for file

Additional file 4 ***Halocynthia *IRK genome Intron-Exon Junctions**. *(A) *Elementary IRK, TuIRKA genome. *(B) *G-protein activated IRK, TuGIRKAa genome. *(C) *G-protein activated IRK, TuGIRKB genome. For the explanation of colored characters, see the legend of Fig. 2 in the original paper. Click here for file

Additional file 5 **Comprehensive listing of IRK genomic genes**. Comprehensive listing of IRK genomic genes retrieved from established genomic data bases. Gene names, intron-exon junctions and chromosomal locations are all listed. Click here for file

Additional file 6 **Alignment of IRK AA sequences**. Alignment of 7 tunicate, 6 *Caenorhabditis*, and 7 bacterial IRK AA sequences. For the explanation of colored characters, see the legend of Fig. 6 in the original paper. Click here for file

Additional file 7 **Consrved sequences in alignment of IRK AA sequences**. The selected AA sequences from the alignment of 115 AA sequences to illustrate an exclusively conserved intron intervening site among the tunicate and vertebrate GIRK group. For the explanation of colored characters, see the legend of Fig. 9 in the original paper. Click here for file

Additional file 8 **Comprehensive listing of G-proten beta genomic genes**. Comprehensive listing of G-protein beta genomic genes retrieved from established genomic data bases. Gene names, intron-exon junctions and chromosomal locations are all listed. Click here for file

Additional file 9 **Alignment of G-protein beta AA sequences**. Alignment of a tunicate, two *Caenorhabditis *and *Anopheles*, a *Drosophila*, and six vertebrate GNB1 AA sequences. Another alignment of a tunicate, two Caenorhabditis, two Anopheles, a Drosophila, and seven vertebrate GNB5 AA sequences is also included. For the explanation of colored characters, see the legend of Fig. 16 in the original paper. Click here for file
