# Supplementary material for: Ancient intron insertion sites and palindromic genomic duplication evolutionally shapes an elementally functioning membrane protein family
Source: BMC Evol Biol. 2007 Aug 20;7:143. doi: 10.1186/1471-2148-7-143 (PMC1999503; doi:10.1186/1471-2148-7-143)
Supplement: Additional file 1 — TuIRKA, TuGIRKAa and TuGIRKB genomic sequences. The data provided represent the determined IRK gene genomic sequences. [file 1471-2148-7-143-S1.doc]

Red characters: intron sequences.

Blue characters: non-coding exon sequences.

Purple characters: no corresponding sequences in their cDNAs but not identified as introns.

LOCUS TuIRKA genomic sequence 18529 bp DNA 25-NOV-2003

BASE COUNT 5997 a 3315 c 3396 g 5820 t

ORIGIN

1

4801 TGTTGAGTCA GCGTGCTGCC GTACAACGCG T**ATG**CGGTAC ACCATTGTTA CAGTTAGTAA

4861 AGTACTGCAT CACACTAGCA CCAGCAAGGC ATTAGTTAAC GCTCGAAAAA GCCAGATAGG

4921 TGAACATTGC ATCGTGAAAG AACACAGGAT GGATTTATCG CAGAAATCTC CAAGATTGGG

4981 AAGAAATATA ATGGATACAG TCAGTAACAG GTGAGAAATC TTTACTTATT CAGTCTATAT

5041 ATATGACAGC TTCTTCGAAT CGGTAACTCG TGATAAGGTA TATACCATAC AGTAGCTACG

5101 CGTATTATAA AAACAAAGAT TTGTGTTGGG TTTTCTAACT AGAGTGTAGT ATGCTTCGTC

5161 ACTGCAGTGG TTGGCTAAAT GTCATTTATC TGAACTCATA ACAGTTTTCA AATGCCAACA

5221 CATATACAGA TATGTTGTTG TTTGCTGGTG CTTATCGTTT CGTTTAACCA GGGATTTATG

5281 CTTGTGTATC GCGTGAAATA CTGTTGTACA CAATATTTGG GATTAACATC GCTGTATTGT

5341 TCACTCCTAG ACTGGGAAAT CAGATACTTA AACTTAATTA TAGTGGCTAA GAATAGCTAT

5401 GTACTTCTGT TGTTACAATA GAATCGAGTT GCAGTGAGGA AATAAAGGAT GTAATCACCG

5461 TAAAACTGGT ATTCTATTCT GAGTTGGTTT CTAAGCATAT TGCGATCTCT ATCTCTATCT

5521 ATCTCTATAG CTCTATAGGA TTTGAGTTTA GAGACAGAAT ATACACTACT GCATTTAACC

5581 ATATATTTTA TTACTGGTCT ATATATATAT TATGATCACG GTAGTTAAAG TACCCCTATG

5641 TTTTCTTTTC TTTATTTGCG AAACGGAATG ACAAGGAACT GCAAATATTC CAGGTATTTC

5701 AAGTCATACT CCATTGGCAT CTGTGATTGA GTTGCAGTGT TATGTACAGT TTTTTTCATC

5761 ATCATAAGTC ATCCCAAAAA CCTACCTCCG TCTGTAAAAA GCATGTAGTT GTGTCTAGTA

5821 GCAATTCAAT TCATTAGATG AATGTAATAG TAGCTGAACA ACGGCCTCCG TGTGGGCTGG

5881 ATGTTCATCA GTGTAGTGCC CATTAGTGTG GGACGTAAGA GTGTGTGTGT GTGGGGGGGA

5941 TTCAGGATGC ACCGACAAAA ATCTTTGGTA AATGGTTGGT TGGATCTTAT TCATTGATGT

6001 ATGAATTTGA ATATATGATT TATTCAGGTA CATGCGTATC ATATGTACCC CTGGATGATT

6061 TATATAATGT GTAATGTCAA TAATCAATTT ATTTCTCCAT TAGTTAGTTA GCTTTCTGCT

6121 CGTTTTTTAG TATAGGTGCA GAATGCTAGG TAGACATTCT CGCACCCCGA TCGCGTTTAT

6181 GTGGTTTCTC AGTCGAGTAT TGATATTAGG ATTCTTATTG TAATAAATCC ACCACTGTTA

6241 CTTATGGTTT TGCGGTACCT GAGGTGGCTT ATTGTTAAAT CTTCTCACGC AATAGACATT

6301 GTAACGAAAA TGCATGACTA TATCGCGTAT TTCAGCATTT CATTTGGCCT TGCGCGGTTA

6361 GATTTTCGCC GTATGCGGTA AATTTTTCGT GAGAAGGATT TACCGATAGA TTAACGGTAT

6421 TGATATAGGC TTTGAACTGT CCGAACAAAA ACCACCATAA TCCCGCCTTC ACAATTCCTA

6481 GGATGTCATT AAAATCATGG AAAACACAAA CACATTTATT ACTGTGAAGC CAGGACCATG

6541 CCATTCTTAT TTTTTTAATT CTCCTTAGGT GAATCTCGTA GAAGCAAATT CCCCGGAGTT

6601 AATAAAATTA TAGAGATCAA ACCATCCCAA TCCGCATCAA TAGAGAAATC CTATTTTCTA

6661 TTATGGCCAG TATACAATAA AGCGACGTAC TGCCCAATAA GAAGAGTAAT TCCATACACG

6721 CCCCTAGCGA CCGATGGGAG TTTCGGCTGA CCGGAAAAGA CACTTTGACG TGCTCGTAAT

6781 GAACATGCGT TTATTAATTA TTACTTAAAG ATAAAGATTG TTCGTTAAAT TCGATCGAGA

6841 AGGAAGGTTC AACTAAGTGC AATACACATT AAATCACTAC AATAGTCATT ATTAAATTTC

6901 TTTCTGTTGC GTTGGATTTC CCAGCTGAAT AGAAATCGCA AATTACTACA TAGAGCGACA

6961 AGAGGAAATT CAATTTAAGA GCGTCCCAAG TTAATGTTAC GTTCCTATAA ATAGACTGTT

7021 TGAAATAATC ACCATTACAT CGCAACGCAT ATAAGAAACA GACTGTTCCC ATGGGGTGCG

7081 TTCTCTAAAA CCGTTACTGA TCAATGAAAT GAATGCTGCG ACACTTACAA AAAAAAGCAT

7141 TTCCTAGTTT CCCTATTTGT CGATGGTAAA GACTTATTAA AAACAAAATC TGCGACCTCG

7201 AGATATAAAT AGGTTCCAGT ACTCTGCTGT TTACTTCGAG TGACGCATAG CAACCATGAT

7261 ATTCTGCAGC ACAAAAACAA AGCATGTACT TTAATACGAC TCACGTTGTA CAGATGTACT

7321 GTCAATAGCA CAGGTTTTAG ATATCGGCAA CGGGACTACT ATAAAGTGAA CGTATCAAAA

7381 CATCTTGGTA AAATAGGAAG CCCCCTCCAC AAACTGTCCT GCGAACCAGA CGTTCACTTA

7441 ACGACGCCTT GTATTGACAT ATTTATCGTT TCAAACTGCG AGTAAATATA GTTTTGATCT

7501 TCCATGCGTG ATTGGTGTTG TCACTTGGCT ATATTGGATA ATCATTTCTT TATTGTCTAT

7561 AATTTAATTT AGATTTATGG AGATATTCAA ATACAGCTGT CCGTGGCATG ATTTATACTT

7621 ATGTGTCGTT CTGCATGTCA TCATTGTGGA AGCTATGGAT TCTTGGTTAT CGGGTATAAA

7681 TTTTCGAATC ATGCGAGACA GTAGAGTTTA AAAGAACGTT TGCGTGTAAT TTGTGTAATT

7741 GATTTTCGAT GATGGTGCTT TTTTTTATTT TCAGGTTTTA TGTAGAAGAT ATAGATGAAT

7801 AAAATTTTAA TGGGTAGGGT CCTATTTTAA ATACATTTGC CATTCTGTCG GTTTGCGCAT

7861 GAAGACATGA ACATATACCA TAATGTGCAT AGTGCCATAA CATACATTAG ATCTCATGTG

7921 AATATATTCC TAGTATTCAT TTCATGTACG TTTCGTGAAT AGACATATGT CGTCTAATAC

7981 TCATTCAGTA TTCAGTATAT TAACTAGCAT TGTATCTTTC AACAGAATAC CCATCGAATC

8041 AAGAGATGCA TACATTCAAA TATCAAATGA CAGCCAAGAT CTAGATCCAA CAACAGATGA

8101 CACAATGAGA AATATGAGTA ACAGGTGATG AGAAATTAAT TTAGAAGTCA GTATTAATGG

8161 TGAAAGCTAT AACGCAAGCT GATAGTTCAT TGGTACTGGT TTGCTATTGG TAGATCAATA

8221 CCAGCAGCGG TGGAAATGGG TTTTAGGGAT ATATTTTTAT TGGACATAAA CTAGACACTG

8281 AATACCATAT AGCTAAGAAG ACTTGCTTTA TTTAATCAAC TTTCACGTTG CAGTAAAACG

8341 AATATGCCAC AACCAGGTGT CAAAATTAGC ATTTTCGTAT GAAACAGTCT ATGATTATAA

8401 CAAATGGGAG AGTACAGCAC CGCACGTTCT ATATTCTGTT TTTTTGAACG GTTCTGTTGA

8461 ATACAGGAAG TAGGTCATTA ATTGATATTT CCTGGGCATA AATCTGACAC TGTTCTACGC

8521 AGCACATGTT TGCCATGAAC TGCTTCAACT GAATTATATG AAATATGAGT AATTTTCTGT

8581 ACTAACCAAT TTCTGTCTCA CGATGCACAT CATGAACTAC GAGTTAAAAA TATGACGCCA

8641 AAATGAGAAG GCAATAACGA GCAACATAGA ATTCTAGCTG TAGCGGCACC TACTGTATTT

8701 CATAAGCATT CTTCAAAGCT TTGAATTGAT AATTTAATGA AGGTTTTAGA CTACCCTCTA

8761 TTCAATATTC TTATTTAATC TACGATGTGA ATGTGCCTTC GCGAAAGTGT AGACACGTGG

8821 ACCACCATAA TCGTGTATAT CATTAAGGCA ACAAGACTAG CATCCCCGAA TATTTCACGA

8881 ACAAAGATAT TTGCTACATT ATTGTATTAT AAGGCTAAGG AATTCTTAGT TACCCGCACC

8941 CTTCGCATGG ATTTGTTTTC GATTTCGTTG TAGAACTAAT AAAAATTGAT TTTTACACCC

9001 ATGTACTTTT GATGGAATCG ATTTCTACTT TTCACAAGTT GTTGGTAGCA AGCGTTTCAT

9061 AAAATTTCCC AAGCGAACTA TTTATAGCGA GGCAATGTAG TCACTATTGA CAATTAATGG

9121 TTTACGACTG TGGTGTATAT TGGTTTATAG ACGAGTAACA ACAATCAAAT CATGTTTCCG

9181 TAGTTCCGTC CTTTATCTTT TATTTTAATT AACTCAGTGA CGGACAGGTA ATATTTTTTA

9241 TTGCCCGTTG TTCCACCTGT GTTGTGTAGA TACCCAGTTA CAGAACAACT GATGTTGTAA

9301 CGACATTTAA CTGTGACCCT TGAGTCATTA CTCTGGTCAT AAACCACGAT AACAAGTTTG

9361 ACTGATGGTT GTTGTTATGA CATCATCATC AAAGATTTGT GATTTCCGTG GTAAAGATGT

9421 TTTGTTGTCT ATAAAGATGA ATAATTATAA CTGAGTAACG GAAGTATGAA TAACCACGAA

9481 GTAAACTGAA TCTAACAATG TTGTTACAAG ATCATCAAAC CCAATTATCA TGACATGCTC

9541 GGGCCTCAAT GACAACAGCA GTAACAGCAG TAGCCTACAA AGAATTGTCT ACGGCAGCGA

9601 GTATATAAAC GATATGCATG CATCCAGCCG ATCTCTAGCA AGTCGGAATA TGCACAGGAG

9661 GAAGAACTGC AGGTAAGTCA ATTATTATTT CACTGATTGT ACTTTAGCTA GACGTTAAGG

9721 TGAGAAAAAT ACAGAATTGA AAAATCCGTA CTGCAGTAGC GTCATAAGAG GAATTAAAAT

9781 TCAAAGAAAA TTTATTGAAT TTATTCAAAA AAAAAATCAT CGTAACCATC CTCTCCCCAA

9841 AAAAGTTCTT CAAATTTTCT CTCCAACCCT AACTCGAACC TGCAAGAATG GGATAAACTG

9901 CATTCTGGTT TACGGTAAAT TCTACGAATA ATCCTCTAAT AACTTTGGTT CTTTCATCAA

9961 CCTTGTCTAA TCCCAAAGCT TGCTAATAAA GAGATTCCGT ATTGAAATTA CAGTATTTGT

10021 ATGGTTGTCT ATCCTACTAC CACAAATTAT ATATCGACCC ACTCAAACTT TTAATGAAAA

10081 GTAGCGGTAT GCGTGGTGTG GTGTGGTGTG GTGTTGAACA GAGTCAACAA AGTTCCGACT

10141 AAGAATCTTC ATTTCTCTTT GCGGTTTCTT TGTTAACGGT TCACTTTACG AAGAATCGCA

10201 ATGGAAAATT ACTCACTGGA TTTGCATCCA CCTGTAATTT TCCTCCGCCT ATGTTGCGGC

10261 AGATAAAACC GTGAGTGCGC AGGAGTAGGA AATAATTTCC TGGGAAATCA GTCAGAATTG

10321 AAGGCAAATG AGTTCCCTCA AAGAAAAAAA CCTTGAACGA GCTTTATCAG TTAAATAGAA

10381 ATAGAGATAT TATAAGTGTG CGTAATACTC CTTCATTCCA CTCGCAATGA ACATACATAG

10441 CAAATATATT TAGATACAAT TTATTAGGTG TTACATCCGA ATTATAACGG AATTACATTG

10501 TAAAGGTATA ATACCATCGA TATTCGCTAT GTTCCATATG TTCGATTACT AGGTTTTCTG

10561 AAGAAACTGA AAGTAATAAT TTCGGAAATA TATGCGCCTA AATACTTTCA CCTCCATGTC

10621 GTCATTATAG CAACATGGAC TTTGGTTAAC TTCAGTTTAC CAAATATAAT TTTCATATCA

10681 ACCGATTTGA TGGGAAGCCA GTGATGACGA GGAGGTTATT TCCTTGTACC GATATAAAGT

10741 GTCAGAGTTG TGCTAAGATC CGTTAAGCAA AATCATGGTT TAGGTACTGC AGTAGGTTGT

10801 GCAGAGCATT CGATCACGCA TATAGCACTG TGTATATAAT GTTTTGTATA CACCGCCCAT

10861 ATACGAGTGA TTTGTCACAT ATATTATGGC GTAACCATCT CCATACATGA GATGCAAGGG

10921 TCACACAGGT CAAACAACAT AGGATTTGTA TACAGTGTAG ATTCCAAAGC ATGTTACCAA

10981 AATCAACAAA GCGACACAGT ATGTTACTGG GAATCATATT TATGAGATGA ATCGAATGTT

11041 TACAGCCATG TAATAAAGTG TAAATGTGAT ACGAGATACA TGTGTCGTTT GTTGTTTTGA

11101 AGAAGTCAGT AGCATTGTTT ATAGATATTG ACAACACGAT GATGTATGAT ATCAGTTTAC

11161 ATGCTTCCTT GCTTGCTTGT TTGCTTGTTT ACTGTATTTC GTCACCGACT GAGAAAACTT

11221 TCGATTACAG TTATCCAATA ACACACTATC CCTCCTGTCT CTTAAATATA ATAGCAAAAC

11281 TTTCGATAAC ACTTGTGAAT AACAACAACT GAACAAAAGA TTAGACGCAC GATAATACGT

11341 TGAAAATTCA TAACATCCGT ATGAGCGAGT GAACTGTGTG TTTATCGTGC GATTAGACCT

11401 ATGAGTATTT TACATACGGC ATCACAAAAA GATACAGCAC ATTGTAATGA ACACGGAAGT

11461 CAGAAGTCAT AATTCCACGT CACTTAATAT ATTTAACTGA TTGGAATGGT AGCTCGGCGG

11521 AAGTCCCTAG CTGTATGCCA TTACAATACA AACCACGTGA CTGTTGTTGG AAGATGTCAT

11581 GTAACCTATT CAATTTAGCT CTGGAAACGA AAATGGCGTC AAAGTATATA TTTTATCATT

11641 TCAGATTCAT CAAGAAATCT GGACATTGCA ATGTCGGCCA CACGAACGTA AACAAGAAAC

11701 CGCAAAGATT CTTGGCAGAT ATTTTCACCA CATGCGTCGA TTTGAAATGG AGATGGAATC

11761 TTTTGTTATT CAGTGCTGCT TTTATACTCA GGTAATAAAA TTTGCACCTT TGAAATAATA

11821 TTTACTTTAC TGGCAATAGC TGTACTAAGC AACGGCAATT TGCAGAATTA TGCAGATCAG

11881 TAGTTCTGAC TAGTATTTTT CTATTGTTCA ATAACCTTCC ATTACGATCG TTCTAAAAAG

11941 CAGGTGTAAT ATAACCAAAA TTGCTCAGAG TTGCAAAAAT ATGAGTCAAA CAAATGTTCC

12001 CATACTCACT GTTTATAATA ATAATATGAG TCCTCGTGTA GATATTTCAC ACAAATTACT

12061 AATTGCTAAT TGCTAATTAC TAAGAAGTAT CCAGATTGGA ATCAAAATAA TATTCATGAG

12121 GCAATTTATT CGTCTTCATG TACAGTTTTT GATTTTTACT TATAATTTGC AGCCTAAATA

12181 CAAAAGTAAA TGGGATTCAT ATTATTATAA GGTGTCCTTC ATTGAGCGAC CACTTACAAT

12241 CACAAAATAG TGTTTCAGTG AGTAATCCTA TATCTGTTTT AACAGCTGGC TTTTTTTCGG

12301 ATTTATTTAT TGGATTATAT CCTACATCCA CGGCGATTTC TCGACAAACC AAACAGATAT

12361 TCAATGTATC AGTAATCTTG AGTCATCTTC CCCATTCACA TCAACATTCC TATTTTCACT

12421 GGAGACTCAG ACGACAATTG GTGAGAAATT CTTGCAATCC GACTTATTGA AAGTGTATCA

12481 CAAAGAACAA CAAATTCTAA TTGAATATAG TTTTTTGATG TAACAATATC CAATTGGAAA

12541 CTATTTAATT CAACACCGTA TTATGTTCTG TAACGGACCA TTCTACAGGA ACTATTGCCA

12601 ATCGGTCAGC GGACCGACGA CCGGCTAATA AATTATATCC AATCGGTAAT ACTCGGCATA

12661 TCATTGTCAT TCACCCGAAA GCTAATCGAA AATACATTGA GTTGATATTA TGATATGATG

12721 ATTTCACAGT GGATAGTCTG GTCCATTAAT CATGCTTCCA TGGTATATAT AGCATACAGT

12781 TGTTAATCAA TCTGGGCAAA GAACATTACT TACGCATTCT TACATTACTT ATGTATTAAA

12841 TGCATTGCAT TTCAAAAATC TACCCAGTAG AGTTAATTTA GCACTCAGTT ATTAATGGGA

12901 ATAAATTTAT ATTCGTATAA CAGGTTATGG ATCGCGAGCT GTTACAGAAG AATGTCCTAT

12961 GGCTATTCTC ACAGTAGTTA TCCAATCAGT ATACGGCTGC ATTATCGATG CATTTATGAT

13021 TGGTCTGATA ATGGCCAAGA TATCGAGGTA CCGAATGATT TATTAATTAC TTTTGGATGT

13081 CAGTGCCCGG AATTGGAAAG CCGAAACTCA ACATATGTTA ACTGTATTCT ATATCAAAAG

13141 TAATTGACAT TTATCCGACA TTGATAAATT GACGTCAATC AAATGAATCA TACAAGGAAG

13201 TGTTTCACGC TGCGGAAGGT TATATGAATC GACAGTACTC AAAACCAGCT AATTCTGACA

13261 GAGATAAAAG AAAATAAGAT AAAAATGACT CATGAGAACA AAACTTTTTA TTTATCTTAC

13321 TTTATTATCC TAATGAGTCA ACGTAAAACG GGAGTGGTGT CTCTGTCATG CAAAAAATAT

13381 TCCACTAATT TCTAATGCAA ATTTCATCTT CCCCCCTATA AAACCAATGC GCCTCCCAAG

13441 GTGATAATTT ATTTACCAAT CCGTTAATAA TCAACAACAA TCTTGCCTTG AACAATGCGC

13501 AAACTGAAAT GAATAACTTT CACAATATAA TATTACACCT GAAGCATTGT GTTCTTTCAC

13561 ATAATATACT TTAATTTGTG AATACAGTAC ATACCATCTT ATATACCGCA TATTTGTTAG

13621 GTAATTCAAG TCGCGACATA ATGGCGGATT CATATGTTCG TGGGTCTGTG TTGACATTTG

13681 AAACATTGAA GTAGTGCTAT ATTATTACGA CGACAAATCA TTTTATCATT GTACATTATA

13741 TTTCTTTACG TTCAGGCCAA AGAAGAGGGC TGAAACACTG CTGTTCAGTA AGAAGGCCGT

13801 CATCGGTATG AGAGATGGTC AATTGTGCTT AATGGTGAGA GTGGGCAACT TGAGGAAAAG

13861 TCACTTGGTG GAAGCAACAA TCAGGTAAGC TCAAAGTAAG ATATTCAGCA GATTGCAAAA

13921 TTTTCAATCA TTCAACTGTA TTTTGTAGCA ACACTCGACC GTTGTTCCTT CATTTTATTC

13981 GTATAAATGA TATCGCGCCC TATGGTAATG GTAATGTTAT TTTTACAGAA TGCAATATAT

14041 TTATTCGCGG GAAACGATCG AAGGAGAGTT TATACCCCTT GAACAAGTCG ATCTTCATCT

14101 GGATTTGAAG AATGATTCTG ACAGATTATT CCTAGTGACA CCACAAACCA TTTGTCATCC

14161 AATCGACTCC GATAGTCCAC TCTACCATTT AAATAAAGAA TCGTTAAAAG AAGCAAATTT

14221 TGAGGTGAGC TTGGTACATA TTGTTTATAC TGTTTTCGTT CACTCCCAAG ATCAATGAAA

14281 TTTCAACCCG CCCGCTAGGG AACGTTAGAG CAGACTCGTG GCGTGCCTTG GTTCCGGTGC

14341 GTCATTTTTT CAAAAACAAC GCCGTCACGA CCTGCACCGG ATCCCGTTGG TCACCAAACC

14401 AACTTTGGTT CAATTCGGTG ACACCGTCTA GTTTTGCATA GAGTAGTATA GTCGGTGAAC

14461 ACACAAACAC ACAGTTCCTA TTTTTTTATA TTGATAGAAC ACAAGAATTA GATTTGCTGA

14521 AACAGGTTTG AACTTTCTGC TGCCCTTTCA GGTCATTGTA ATATTGGAGG GAATGGTTGA

14581 AGCAACAGGC ATGACTACTC AAGCACGCGC GTCGTATGTT CCAGATGAGA TAATGTGGGG

14641 ACATCGATTT GAAAACGTAA TTACGTTTTC AAGAACGAGC AGATATAACG TCGATTTTCG

14701 AAAGTTCGAT AGGGTATAAA AACTAAGCCT TTTTCTTAGC TTTCACGTAT TATGGAACAT

14761 CAGATAAAAT TCCAATCGTT AATTTCATTT CAACAGTCAT ACGAAGTACC GGAAACACCT

14821 AAATGCAGCT CTAAATATCT GCAAGATATG CAAAATGCAG ACACCGTATC AGAGAGAATA

14881 ATGTTGCAAG ATAAGGAACA AAATCGGAAG AATACATCGT AAGTTTTGAA CCTGCAATGG

14941 ATATAATCTA ATAAATCTGA AGTACTAGTT ATATGCTTGC AACATTGACA TGGTACTAAA

15001 CTCCTAAATG TTATTACTTG TCACAGTAAA GACGCTACTA GAGAAGACTT CAGTGTATCA

15061 GATGGACGAG ACTCAGGATA CACAGCACCA GAAGATTATC AAGCGACAGA CAGCAACAAA

15121 ACACTAAATA GTACATCGAC CTGCGGAAGC TCTTACATCA ATCATGATAC CGAAAACCGA

15181 AGGCGAAGTT GGACAAAGAA GGCGATCGAG GACACAATGA ACAAAAATTC GAATCAATAC

15241 GAAAGTACAA ACATGGTAAA TATTCTTTTT TCACCGTTGT CTGTGTGCTT ATACGGAGAC

15301 GTATCTTTGA TCACTCCAGA TTATCTTTTT TTTATTTCCA AGTTTTCAAA TCGTAAAACC

15361 TTAATGAACT TACAAAATGG AGGTTACTGA TTTTAAGAAA ATGAGTAACA AATTTTGGAG

15421 CATTACTGTA GCCTATAACA ATTATTATTA TTAATTCCAG GATAAATGTC AGACATCTTC

15481 AGATATCTTG CCAAACATGA AACAAGATAT GCAGATAAAG ACCAATCGTA ACAGCGCTGA

15541 CATGACAGCC CAAATGACCC TCACTCATAA CGAGAAAAAC GAAGCTTTGA ATAGTACAGC

15601 TCGACCTCGA AGTGGTAATG TACGCATTTC AACAGCTATC CCGCTCGATA TATTAAAAAT

15661 ATCAACAGTC ACAGTCCCTG AGGAGGTCGC CAAGAACAAA AATGGAACGA GTTTCATAGG

15721 CGATCAAAAT CCTAAATCGA ATCTCAAAGA ACTGCAGAAC AATAACGAGT CAAAAAAATC

15781 AACTGAAATT CTTAGCGGAG GAAGAAGTTC ATTGCTTGTT CCAAGACAAA AGAAGACGCA

15841 GACCGAGCAC GTGAATATCT TAGCGACGAC TAGACAAGAC GATATTGTTC AGCAAATAAA

15901 AGAAGACCCC TTAGAAAATA AATCTTTACT AGACAATACT TCGCCAGTAA AAAAAATTTG

15961 TGAGGTCGAA ATGCCTGAAA TCAAAATCGA G**TGA**ACACAG AACGCACTGT ACATGAGCAA

16021 TAAGAGTCTA TACCTACCTA CATAACAAGA CATTACGTTT CTACGTGGTG TTAACTGAGA

16081 ACTGCAGTGC TACCTGAGTT TGCTTAGTGC TAACCACGAA ACCTGCCATG CAGATCCTTT

16141 ATCTCAGTGA TTGGAGAATT CTCGTCTTTG AAAACTTTAA AACCTACTGT ATTGTATATA

16201 GTATATAGAC TTTATTAATT AATTACTGAA TTTATTCGAA ATTACTTCTT GACTACCCAA

16261 AGTCAATTGT TTTTACTTCT TCCAATCTAA ATAAAATATT TATTGAAATG GTAATTAGGA

16321 AATTGATCTT TTGCCCCCCG AATAACAGTA TGTATCTCTG TCGGACTAAG AAAATTACTT

16381 ACGGGCTCAC TTAACAAGTC ATGTAAACAT AATTAATCCA CTTCCAGTCT GAAACGCAAA

16441 TGAACTAAAT TTTTAATTTT GTTATTGAAT TGTCCATTAA AACTATATAT TTATTATTAC

16501 AATCGAAATA TTAGGTGCTT CAAAATTAAT ATATAATTAT GAAGTATCAT ACTGTTATTT

16561 GTAATAATTG TTTTTAGTGA ATTTTTCTCG CTTTTCAATG ACGCTTTTGC TTTTTTCCGT

16621 TGTTTCATTT TATTTCATTA CAGGGGGTAA GTAATAATCA TACATATTTC ATTGTAGAAC

16681 TTATTGTAAA CATACTAAGT CATCGCTGTC CCAATGAATA TTTAAGTAAG GGTCACACCA

16741 TAGATCAGAC GATGCAATTA CGACGAGCAA TGTAAACAAC AGAAATTCCC GTTTTCTTAC

16801 TCCGAAATTT TCGTCGGTAC TGTGGACGTA GCCCCGCTGT TACACTTAAG TAATTTCTAG

16861 TGTAAATAAA TATATGAGTA GGAATATATT TAATCAAAAA CTTGCATCGT CATCTAATAC

16921 AAAAGGATTT AATGACAGCG CTCCCTACGC AAGCAGAGCC AACCGTAGAT AGTTTGGCTC

16981 CTCTGGAATA ACTACTTTAG GCATTATTCC TCGTAGCCAA ATAGTGGAAT AAATTTATGG

17041 ACAAAATATC TTGATCCTTC TTGTTTATCA TACGATTTAT CAAGCATTAT GCAATTTGTG

17101 AATTAGCATA GTGAAATTCG AATAACGCGT ATGCTTTTAC CAGTATTTGT CGTTTTAGTT

17161 CCATTGCTTA AGCACGTTTC AATCAAATAT ATCCCATGTA GTATGTCTAT TCATTACATT

17221 TGCAAATTAT TTTTCTTCCT TTGCCGTCAT TCATGCATAT GTGTGAATCA TTGTTGTAAA

17281 TATATTAATT GCTTTTATTT TATTACCATG CGTTGTATGA CGTTATTTTA CACGAAATGA

17341 ATTTTGGCAA TAGCCAATCG CCAATGAAAT ACCATGTCGA CTAATCCTTT TACATTTACT

17401 CGCTGTTTCG TCATTGTGGT GGCGATAGTC ACAAATTTGA ACAAAATAGA AATCTACAGC

17461 CTATTCAAAT GTGTCATTTT TACTAAAACC TATGACAGAG AATTCACGAC TAAAAATAAA

18481 GAATTTTGCT TGTTCCATTC TCCATTGATC CGTTGACCTG CAGGTCGAC

//

LOCUS TuGIRKAa genome sequence 23800 bp DNA 18-DEC-2002

BASE COUNT 7950 a 4188 c 4276 g 7386 t

ORIGIN

1

8101 TTTATCTTTA GAAGTGAAAG AACATGGAAA TGAAAATAAT AGTTGTTTAA ATAGTAACGT

8161 AGTGGAAGAT AAACGTTTGA AACGCACAAT AAACAATAAC CAACGAATCG CTTGGAATAT

8221 ATTCATTGGT TATCGAAGGA **ATG**GGTGTAA TCAGCAACGT AAGTCCGGAT GTGACCAATA

8281 AATGGAGAAC AGATTCACAA GTTTTGCCGG CTTTAGGAAA CAGCAAAGCA AGGGAGTGAG

8341 TAAAACTAGT AAATAAAACT AAACTTCATG TTTAAACTTT GCGGTGCTTC CAGATATCAG

8401 GGCGATAAGG ATATATTACC TTGCGTAGAA TACCCAATAC TTCTTGAGAA ATATCAGATT

8461 ACATATTATC ACTGTTTTTT TCTCGTGGAA ATCGGGAATG TACTGATTCG TTATCGCTCG

8521 ATCAACGAAA AAATATATAA TAAAACATAA GCTAATAAGG TGGCAAACAC ATAGCATTTT

8581 TTATCAGACT TTGATGCCTC TACTAGTGTA TTAATAATTC AGTGTGTTGG TTAATTACCA

8641 AACGCCACAA AGATCGCGGA AGATATTGCG AGAAGGTGCG CAGGGGGTGA AATATTCAAT

8701 TGTAAATATT TCAAACAAAT TCTTGATAAA TGGACGATTG TAGTAAATAT TTAAAAAGTC

8761 TCCCAAGAGA ATAGAATGGT CGCTGTGAAT TGACAATATA CAGCTGACGC TAATGAGTTC

8821 ATTAGTGTCT ACATGTTATT ACACCACACA CCCATTAGCG AGTATAGCGA CTAACAGTCC

8881 AAACTGATAT TTTCTGTAAT ATCATTTCTT CCTCTGTATA AAAATACCCT TCATATTATT

8941 CACTGATTCG TGGTTCCATG TTATTTAGTT CGATATACGT AACATGGATA GTATTACAAT

9001 ACATATGACC ACGCTCAAAG CGATTATTAT AAAATAACTT GATACACAAA TCAAAATCGA

9061 ATCATGGCCA TAACCAGATA TATCGTAAGT GTTACGAGGA CTTAGTAAAT ATACCTGTTT

9121 TTCTCCCGCT AACTAGCCAA CTCAACAGTA GATTCCAATT CAACTTAAAT CCACTCAGAT

9181 ACAGTAGTGA CACTCCCTAA CCCCTAACTT TAGAACTTAG TCATTCGAAT TTGGTTTCAC

9241 TTATTTCCCT AATCAAAATC TGTCATAAAA GCGTTAGATT TTATCAGGAG TTTTTTGACA

9301 ACTAAAAAAG TTTCTGGATA ACTAGACGGT GAATTTATTT CGTGAAGTTT CTTATGTATA

9361 GGTCTATCGT TTCCATATGG GTCCATATGG GAAATTTCAT ACATCATCGT TCGGCCACTA

9421 GATAGTATAT TTCAAAAAAC TGAAAAGGAA ACGGAGGGAT ATTCGAGATA GATAATTTAC

9481 ATACGGGACA CTATTGATTC CACTGGTTTT TATTACAATT CTAATCCTGT GGGGGAATTA

9541 CAGTGTCCCT ACTTACACAG AAGAATTGTT GTCTGCTGCG AAAACTCAAT TACGTTCGTA

9601 ATCAAAATCC CCTATGCGCG TGCGTACTAG CAGAGTAACG AGCGACTTAC GTCAAATATT

9661 ATGTAGAATA ACGCAAAAAA CACAGTCAAT AAAAATTTCA ATGTGATAAG ATCATATGAT

9721 CCTGAGTACG GATTATTTTA CTCGTGATCC TTTCGTGGTT TTCCGTGAAA ACTAGAAATT

9781 ATAGATAAAT ATAAACCTTT CACTTAGCAT CAGATGACTT TCCTAACCCT TTGACAAATT

9841 AAAATACCTT CTGAAAGTGC CACATTTTAA ATTAAAGATA TTTCCAGGCT ACAAGGCTAA

9901 CAATGCAATA GATATTAAAC AAGGGGTGGT CTTTATGGTC TTTACAGAAA CTTATTATTT

9961 AGGGTAACTT TCAAGGTAAT TTTAGTAACA CACTTCAGTA TTTTCCCCTC ATACGATAGG

10021 GCAATGTCAA TCATCATTTA ATGCAGCATA GTTTATCTCA GAGAAAAGTG AAAGGCCTTT

10081 GAAATGAAAT ATGAGTCGGG ACTGTTAGTC ACTGTACAAA ACTTTGTAGT TTTCATTTTC

10141 ACAGAAGCTA CGTAACGCCT TCACAGAATT TCAGCTGGTA TCGTCTGGAA TAAAGTTCCA

10201 GATGTTCCAA AGTATAAAAG AGTATTTTGT GGGAGGTAAA ATCCCTAGAA ATCTACATAT

10261 TAGGTTACAA CAAACTCCGA AGAAAAAATT ATCTAAATGT CCAATCACAG TAACCAAATC

10321 ATAGGCGGAT AGCCAGATGG AAGGTGTGGG TTCAAATAAC TGGAATCCGA TATCTCAATA

10381 ACTTTGAAAA AAGAACATGT CCGAGAAAAT GAGGACGAAT GGCATCCCTA GTCGGAACCG

10441 TCCTAACGAA TATTCTATAT ATTTTCCAGC ACGCTCTTTC AAGAGAAGAT GGTTCGCACA

10501 CATTCAAGAA GACCATCCTG CGTTCCAGCG ATCACACGGG ATACCCCAGG TGGCGTGAAA

10561 CGACCGACCA AACGAAGACG ACAGACGAGG TAAAGATTGA TTTTCAAGGA AAAAAAAAGA

10621 GGAGGAGAAT GATTGGTGTC AAATTTGGAA ACGTTGCGAC TCTAATCCAA GTTTTTTTTG

10681 ACGGCTGGGT ATATAATTGC GGTATAAGAT TTTACTAGGA ACAATTCCCT ATTGTATTCT

10741 ATTCCCGACG GTATTCTTTC GACATGGCTC GGTAAATATT TATACGGAAT GTTTCCTTCC

10801 TAGTTAATGA AAAACTATTT CCCGCTATAC CAAGGGAACG ACGAGAAAAT TATTCAATGA

10861 ATAAAATGAC AGGATTTGTC CAAGTGCTAA TTAGCTCATG CTGTGAATCG ATAAGCGACA

10921 ACTACAGTAC GGAATAATGA AGAGAAGCGA TCGAAAATAA TGTGATGCCA TTTCCGTCAA

10981 ATCAATTTTG TATCGTTGCA ACAATTCTTG AAAACATACT GTACTGATTC CGTGTGCTGT

11041 TATTCATTTC TACACTCTTT TCCATTGATT TACCAAGTCG ATAATTTTAA ATACTCCAAA

11101 TATATTGTAA AGTACACTCA GTGACCTTCC TACGGACGCC AATAATGCCT ATGGCGAGCA

11161 TAGGAATTGC TCCCCTATCC AAACATTTAA GGTACATTTT TCTCTTTTAA TTACCAATCA

11221 CGAAACGCCA TGAAAATTAC AATATATTGT TTCATTCCTT TACTTATATA GTTTTATTTC

11281 ACGGTATGAA GGCTGTGAAT GGTTCTGAAT CCAAAACTAG GACCAAGATC TCGTCAATAT

11341 GGACTAGAAA TAATAGATGA TTTCCGACCG CATATATCTA AATCTATATA TCACATCACC

11401 AGCTGATAAG ATAAAAGATG TTCCTAATAG AACTCACGTC CTTCAACAGA ACAGTAGCGT

11461 ACGATGAAAT GAGTTATAAT CAAAATTTTT ATAAAAATGA AGCCATCAAA AGCCACCACC

11521 ACTTTTCAAC TCCATTCAAA ATTCATCTGA AACAAAACTA TAATCATTCC CAATAGGTGA

11581 TGAGAAAATT TAATTGGAAG GCAACAACAG TGATCCTTTG ACAGTTTATA CTATGCCCGT

11641 GGACGTCAGA AAAATCTGGT ATTTTCCTAA GACGTACAAT AATTTGTAAA TATATCGGTA

11701 TTACTTACTG ACTACTTACA GGTCTTAGAA TGGAAGCAAC AATATTTTTA TAAGTAAACC

11761 AACAATGAAA CAATATAGAT TGAAGAAATG CCACACCTAT GTATCTTATG TACAAGTACT

11821 CGACAAATTA TGAAGGACAA ACGGGACAAA TCATTCAGCC GTATCATAAA ATATATCATT

11881 CAACTGTAAC CGAAATAAAC TACTAGAATT ACTTAGCTAT TTCACTCCCT ATAGATCAGA

11941 ATCGCTGATC GCTGGTAGTA GTAGTTGCCG TGTGTGTTCA ATTGAGTTCT GAAAGGGGTG

12001 GACTGGGCTT GCGGTTTTTT TAGCAATGCC TGCTGGGTCG TTACCACTAG GTAAATGAAT

12061 GGACCGTTGG GCTGGAGAAA AAATGCTGAA AATATCACAA AAAACCCCTA CTGAACTCTA

12121 GGCTACTGAA TTCTGATATT AGGCGATAGG TACAGTATAA AGTAGAGTAT AAATGCAATA

12181 CAGTATTAGT CTCGCGTACT TGTTTAAAAA GTCTTTCTAT TTACTTCATT GAACAGGTTT

12241 GTTACGAAAA ATGGTCACTG TAACGTCAGA CACGGCAATG TTGAAGATCG GTCAAGGTAT

12301 TTATCCGATC TCTTCACAAC CCTGGTCGAC CTAGAGTGGA GGTACAACGT CATGATTTTT

12361 ATATCGACCT ACACAATAAC GTGGCTGGTG TTTGCCTTCG TTTGGTGGTT CATATCATTT

12421 TGCAGGTAGG AACATAGTCT ATTATTATCA CTGTATTATT CGGCAAGATA TATCGTGCTT

12481 GACAAATCTA TAGTATATAT ATAAATTTTT CAGAAACAAC GTGTACAGTC ACTCCCTACA

12541 TTCAGATTTT ATCCGGTTAA AAAAAATGAT ATCGGCTCAA TGAGCCTACT GTATCATATT

12601 TACATTTAGG CTTAACAACC TATGTATATT ATCATGGATG AATGGTACAG GCACGGTATA

12661 TCCTGTATAT AAATGCAGTT CAATTGCAGG TCCGCAATAT GATTTTTCCA CCCGCAGTTG

12721 TTTTTTATAA GAATATATTT AGAAGGGAGC GAGAAATGTT TGTTTTGAAG AAGGAAAAAT

12781 TAAGATCAAT TAGGCGATTC GGCTGCAGAT ATTATGCATG CAATCTATTT AAAGATTCTT

12841 TAAGTGGCTT TTGTTTGCAT TTAAATATCC TGAAAATTAC TCTGATGTTC AGTCTAAATA

12901 TTATGAAGTC TCAAGATATT CGACCTGCGA ATTTCGTTAA AATGTTTTTC CGGCCCCGAT

12961 GATGATAAAT CTAAACTACC CTGCTACCTA ATATATAATG GAGCTCGATA GTAGTTTATT

13021 TATATATGTA ATATGTAAGT TATTCGCAGG GTCCGTATTT CATACTTTGA ACATATTATT

13081 TTAACATCCC TTGTCAAATG ATATTATTAA AATCATGTGC GCATCAAAAT ACTTATTTAA

13141 AGCCCCTTTT TTATTTTACA ATTTTTTGAA GAACAGTGGA TTCATTAGGG CATGAATTAT

13201 GGTAGGTATC CATAAAATTA TTTCTCGACA AGGGTGCTAA TTTTTACGTA CGCCCTTCTC

13261 AATGAACGTT GTTACATCTT GGTAGGCGAA GTCACAAATC AGAAACGTTT TACATAATGA

13321 CCAGTATTTC ATAAAATATT CATCAGCCCT TTAGCTCCCA ATAAACGTGA GTGCGTGAAA

13381 CAGAATTACT TTGTAAATGT ATTAGTTCTG AGAGTTTTAG ATTAAAAATT CATGAAAAGC

13441 TCCTGCATAG CTCTTTAACC CGACAGACAA AAGAGCAGCG TTTAGGAGTA CCAGTCGATC

13501 ATAAGGTCAC ACGAAAAGCG AGGGGAACAA ATACAGGTTA AGGATTGCTC TGCAGGTGAA

13561 GCAAGGTTGG CAATCAATTG CGTGAAATCC CATAAGGTCA AATAAAAATT CTATGATGGG

13621 GAAATAGGTA GGAAGCGATT TAATCGCCTA TTTCATTTCG TTCCATTGTG AATCGCGAAT

13681 CGAAAATTAA TTACTGTACA GTATTCGAAA AACTCGCTAT GTGATTAAAC CTACAGGAAA

13741 AATGAATTAT TCGAAAATTG TCTTATTAAA AAAAAGGAAC TCTCTTAGTA GTAAGGGAAA

13801 AATGGGGCGG TGTGTTGGGT GTTTAGAAGC AAGTTAAATA CACAACTGAG TGTGCGGATT

13861 GAAAATGATG AAATGAAAAT CATTATTTTC TCGGATCAAA ATATAGCTCT TGAAAAACGA

13921 TACTTTTGAA AACGTTTCGA ACTGTTTTAT TTTATCATGT TTTACGCCAT TGGGGAATGT

13981 CACCGCATAA TGTTCCTTGA AACGGAAAAT TATTATCAGA TTTCCGGCTT ATTTACATTC

14041 TACAAGGTGT TACAATATGT TAAGTATGTT TGTTGTTCCA GGAACGACCT GAATCTCGAT

14101 GTCAAAAACC AAACGGTTTG TGTGATCGGT ATCAAATCAT TTACATCGGC TTTTTTATTC

14161 TCAATAGAAA CTCAGGTGAG TAACATTACA TTACATATAG TGACGTTGTT TTAAGACAGA

14221 CAAACGAACT CGAGTTTAAT AATTTAGAAA GTTTTCCTTA TTTTTTGAAT TCACTGATTA

14281 GATTAGTTCG AATTCTATTC TACTCAATAC CACTACTACT TTAGCTTCTG TCTTGATTAA

14341 CAAAAATCCT GACCAGCACA CATTCACCGA TTCCATCCTC TACTTTTAGG TCACTATCGG

14401 CTATGGGACA AGAGCAATAA CGGAACATTG TCCAGAGGCT ATCATATTAC TTCTGATTCA

14461 AAGTCTGCTG GGATCTATTG TCGACGCATT CATGGTAATG ATTCATACAT GGTGCCTCCT

14521 ATCTTGTACT TAATCTGTCC CACAAGCAAT AAAATTTCTC TCAAATTTAA AGCCTGGAGC

14581 GTTCGACTGA CTTAATACAT TGTATTCAAT CGTCTTTCTC ATGATATTTT ATTTCATAAC

14641 CATCTTTGGT ATGTGGTATT TAAATTTGAT TGCAGATAGA AACATAGGTT GCCTTCATGA

14701 GAGAGTATTA CGTTGGTATA AAGTACATTA AACGTAAAAA TAAAAATTCT GTTCGGCGTT

14761 TTGCAAGTTT TGTTTCGGTT CTTTTCAGGC ACCATATAGA CCCTGTAGCC TGAGCAAATT

14821 GAAGTTGATT GTGAAATTGT TCATAATTTT CAATACCTCC ACAACGCTTT ACGAAATCTT

14881 GGATTTATGC CTTGGAAAAC TTTCACAATT TTTTTATTAC GCTTATTACA GGTTGGATGC

14941 ATGTTTGTCA AAATTTCACA GCCAAAGAAG CGAGCTGAGA CGATAATGTT TAGCCATAAA

15001 GCCGTTATGT CTCTAAGAGA CGGTCAGATG TGCCTAATGT TCAGGTATAT ATCCTTGGAT

15061 GAAAATAAAA GTCGTGAATA AGTTCTTGGA AAGTGCGATG ATAAAAAATT TACCATGGGC

15121 CCATTAAGGG TGCAATGTGC ACATTGTATA CATGTATTTT AAATAGGGAT TTTGCCAGGA

15181 CCATCAAATC AGTTCAAGCT AACCGCAATT GTAAGCATAT CGACGTCAAA GTAACAAGTT

15241 TCGACTGTAT TTATGGCATT TTTGTGTTCT TTTTACCTAT TTACCTATCA TGTGTATTTA

15301 CTGGTTAAGT CCATCGAATT GGGTTCAGAT TAGGATCTGA AAACGAATTA ATTCACTTTC

15361 CATTATTTAC AATGGATATA TTTGATTCGG ACCTTGAACA ATTCAGATTT CGAACGGCCT

15421 CCTGTAACGG ATTAAGTACC ACTGTACTTG CATATTTCAC ATAGCCGTCA TAAAAAACAC

15481 ATATATTAAG TTTACTGAGT AATATTCGAC ATAGTTACTG ATACAATGTG TGTGCAGCTA

15541 TCATACCCAG TGACCATCGC CTAGTAATTT ACTAGAAGCA GCTTTAACTC ACAATAGCTC

15601 AATGGGAGAA TTCCAATATA ACAATACTAC ACTCCGCTTC TCAAGCACCG ATCACACCCT

15661 ACTGCTGTTT TACTAACTTT AATTTCAGGT TTATTACAAG TCAAATGCCT CTATTCCACT

15721 CACATATTGC TTTTGCACAG GGTCGGTGAT TTAAGAAATT CGCACATAGT CGAAGCCCAG

15781 ATTCGGGCAA AATTAATAAA GTCGAGACAG ACGCAAGAAG GTGAATTCAT GGCACTCGAT

15841 CAAACCGATT TTAACGTTGG ATTCACCACA GGAGCGGATC GACTCTTTCT AGTGAGTTCA

15901 TATAAAACAA ATAGCTCCAT ATAAAGCGCT ACTAGCTAAA TACAAATATC ATTGAAGCTA

15961 ATTTACGCCA CTGACTACTT CACCACAAGT GGAGTTGTTA ATTTTGAAAT ACTTTTGGAG

16021 TAAAAAACTT AAATTGTATA TCTCGTATGT ATCTTGTATA TCTATATCTT TTATCTGACA

16081 ATTTAATTCA GCGACTTGAG CGATAATAAA ACAATATTCC GAAATATGCA AAGATGTCAC

16141 AGTTTTAAAA TTATTCGGTA CACATATTAC GTTGAACTCA AGTCAATATT ATCGACAAAG

16201 TGCCTGGAAC TCATCCAGAT AACTCGAAAT GCTGATACGG TGCAGCGTAT ACTAAAAGCG

16261 ATAAACGTAA CCTCTTACTG TTCATACCGA GAGCGATACG AACTGTCAAT ATAATATATT

16321 CTTATTCATC ACTTTAAATC CTTTCAAAAT TTGGTTGAGT AATTGAATGA CCAACAATGG

16381 ACTGCACTTA CATTGAGTTT AAAACTATTT CGATTTTCAA ACTACAGTTA CTAAGTCCTC

16441 GGTCAAATTT TGTTCGATTC AAAATTTAAC CTGATAATTT TATGTTTACT TTAAACCTTT

16501 TAAAGCTGAA TTAAATCTTT TTTATCGAAA AGCCTAAAAT TGCATGGCAG TATTATATAT

16561 GATTTAAAAT AATAACTTGC CGGTGCCTCG AGATCTTAGT TGCCGTCGGG AAGTAATATT

16621 TTCTTTATTA TTCAAGGTGA CTCCTCTTAT AATTTGTCAC ATTATCGACG AAAAATCACC

16681 GTTTTGGGAG ATGTCTGCAG AAGACCTGAT AAACGAAGAG TTTGAAATAG TTGTTATTCT

16741 CGAAGGAATG GTGGAAGCTA CAGGTAAGTT GACAAGTTAT GGTAGTTTAT ATAGTTCCAT

16801 CCATATTTAA TTTCTAAAAA CTTCACAATC AATGGATAAT CAACAATCTG GAAATTTGTG

16861 AAAATAATAA TTTTCAATGT AGATATGCCC AAACCTCCCT GCCTGCCTGT TTGTAATATA

16921 GTACACTGGC GAAGAGAGTG AAAGTGTCAA TTTCGACTAA CTTTGAAATA ATTAGAAATC

16981 TATATCTGTA TAGCATCATT TCAAGTTCAC AAGAGGGAAT TAAATTGCTG AATTACGACT

17041 AACCATCAAC TTCTTCGTAA GATTATTTTT TCTTGCAAAG TTAGAACTTA AGTTACCAAA

17101 CTTATCATCT AGCAGTGATC CATTTTCTCT AGTTTTTCGT CACTTAATAT CGACCGGTTA

17161 AGTTAGTTAC CGTCTAACTT TTTATCGCAA GTCAGCTTTC TCGAATGCAA CCCCCCATGA

17221 ACCAGTTTCA CTAAGTACAG CAACTCGATA TCTTGGAAGA TTTTTCCCCA ATTCTTCCAC

17281 CGATAACGCT ATCAATTAGG TAAGCGCTAA CACCAGAAAT ACATGGCTAG AAATAGAAGT

17341 GGGAGGAAGG AACTTTATTC CTGTACCTGA GTCTCAGATT TCGATGGTTA GACATGGATT

17401 GTTTGGGGAT GGACAATGCC ATTAGGTACC GTAGGCATGG CAAGTATGAC ACGTGCCATG

17461 GGCTCGACTT GTATGTTTGA GTTGATAGTA TGGTGAAGTT ATCTTAAGGA TGAGCGTTAC

17521 GTTTAGATGG GAACGCAATT TTTGTGCTCT CCATATGTGC TATTTATTGT AGGTACGCCG

17581 CTTGGTACCA TATCGATGCT CGCGTGACCC TAACGAACGA TGGATATATT AATGATATCA

17641 GTGATGTATA TATTAACATG AATCTCTAAA ATTTTGGCTA ACACTACAAT CCACCCCGCT

17701 TCATTCTGCT TTGACCTCAC ATTCAGTTGT GGTCGACTAC ATTTTTATCA GTGTATCATC

17761 ACGAAAATCA AATGTCTTCA GCGATGTCAC GTGAATGTGG TCAGCCAATC AGAAATCAAT

17821 ACATATTTTG TTTGAAATCC AACATAAACT ACCTTCTCCA GTCTTAGAAA TCCTGGAATC

17881 TTTAGGTTAA TTTCGCAAAC AAGTTCTTTG GAATGCAAAA GTTCCGCAAA TCAATAACGC

17941 ACGTGAAATT ACGGGCCGAT CAGAAGAATA AATTCGTCGA TGTCATTCTT AATTAGATCA

18001 AACTTTGCTA CGGAGAAGAT TATTGTGCCT AATGCGTTGC TGTTCTAATG CGCTTGTATA

18061 CGCTTCACTT CTGCAACAGA AGATGGAAAT CAGATGATTT AGAGTGACTC ATACCTACAA

18121 GGACGAGAGA TAAGATTCTT AAATGACATT AATCTTTTGG CTCTTGGCAA ATTTCCGATG

18181 AGTACACAGC TTCCGACGTC CGGGTCGATA ATCACCGCGT TTCTGCTTTA TTCAAAACAA

18241 AGCTAATATT TCGCTCACGG GAATCCTTGA AAATTATTAC GAAAGCGTAT TGACGAGGAG

18301 AAAATAAAAC ACGTTCACCA ATCACTGGAA AATTTAACAA CATATTTATT TTCGACGCCA

18361 CTTAGCTGGC GGTTTAATCC TATTACTTTG TTTAATCCCA GGAATGACCT GTCAAGCTAG

18421 AAGTTCATAC GTTGAAGATG AAGTATTATG GGGTCAAAGG TTCATGCAAG TGCTCATGTT

18481 GGAGAAAGGG TACTTTGAGG TAAGTGATTT CCCTAACACA CATTCAAAGG GATTATAGTA

18541 ACATGCAGGA AAGTTGGAAG AATGTGAGTG ACTACTTTTG GGAACTAATC TACACGGGGA

18601 TGAATAAGAC CATGGCTGAA TTAAGCTATT GAGAGACCTG TATATCTTAT CGTGTTGTTA

18661 TCCATCTCCC AGTTTTAAAA TCTGGATATA TGAAAAATCG ACAGGAAAGC ATGGGTAGCC

18721 AGCAGATCCT CGCGATTTAA AACTAATAAT AATATTATTA TTATTAACTC GCTGTATTAT

18781 AACGACGCCC TCAGTGATGC CCTGTGATTA TAAGTTTACA GTGGAAGCCA ATAGCCTATA

18841 TAGCAAGTCT CATAGAATGG TGAGACGTGA AAATAAGGAA AATAATGTGG ATTGATTATG

18901 AAAACTGAAT AGTGATTAAC TGTCCTGGAC CGTGGAGAAA ATGATACTTG TTGAAGGAAG

18961 TTCATCTCGA TGTTAAATGA AACATTGGAT TTCAAATATA ATCAAAAAGA TTGCTAATAA

19021 TCAAATTGCT ACTATTGTAG TATGTCAAAC CGACATTGCA TGACGTCAAA TTGAAGAGAG

19081 ACTCGATTTT ACGCATTTCG ATGATGACCC TGTTAACGGC ATTACACCAA TCCTAGCTGC

19141 AGTAAACGCC GGTTTTTAAG TGCTTCCTAT ATATGGAAGC TATACTACTG CTGTCGCAAC

19201 TTTAGCACTA TCTGCGTGCT TTGTTGTACG TAATGGCGTA CCTCTTGTTT CAGAATTATA

19261 ATAAAAGAAT CTCACACATG TCAAAATTCT AACTTATCGT AACCTATATT ATATAGGATT

19321 CATATGAGCA CGATTTATTT GTAGGTGAAC TATAACAATT TTCACGATAC CTTCGAAGTA

19381 TCCTCGCCGA CTGCTAGTGC AAAAGAACAA GCAGAAGAGA GAATAAAGGT AGTATATATG

19441 AATGCAACCA TATAATAATG GGCTAGAGAT AGGTTATTTG ACTGATATTG ACATTGAAGA

19501 TATTTTTTTA CAGCAACGTT TGAATGAAGC TAACAATTCC CCCGGTGCCT TAAGTATGCA

19561 TAGAACACTT CCACGATCGC CCAGAGTACA GGAAAGCGCG AGTTTGAGGT AAGAAGTTTA

19621 GTTTTTATTA ATCAACCAGC ATAGATACGT GGCCTTGCTC TTGTAAGCCT TTTAGTTCAT

19681 ATGTATGTCT GTCGGTCCTC AATGCACGTC CACAGTGCAA GCATCTATAG CGGTGACAAG

19741 TCACCATGAT GTGTTATTCT GTTCGATAGG GGGCGTTAGA GCGTCCCCGA AGCGTGATCG

19801 GACCAGAAGA AACTTTTTGG GGATATGAAC ACCGTCACGA CTCGTGTAGA GTGCCCTTAG

19861 TAACCATACT AAATTTGGTT CAAATCGGAC CTCGGAGGAG AAGACGAATA GACACACACA

19921 CACACACACA CACACATATA CACAAATTTT CAAATATGTA AGATTATAGG ATATATATAA

19981 GATATCGGGT CAAGTGAGTA AACTGTCCAG AAATGCCCTG AGCGCTCTAG TCTTATATCT

20041 AGCCCCACAC AAGTCGTCAA GTCTCTAGAA CAGTAAACAG ATGGACAACG TGAACAAAAT

20101 CTAACTTGCG ATTGCAATGA TAATAATTAC AGCTCGTCGT TCCCAACCCC GACGGTGCGA

20161 AGGAAAAAAA CCTCAATTGC AAACAACTGT TACCCGGACG AAGTGGAAGA AAACAATAAA

20221 AGCGATTCCC CTGGTATCGA AAAAACCAAC GTCCAATCGA ACGAAAATAA TAACAATAGT

20281 TTGCAGCCAG TACTTGCGAG TTATCCCGTG CACAGCACGC TCGGTCAGCA ACAGACATCG

20341 AGCCAGAGCG AGGCCGACTT GCTGCGATTG AACAAACGCT TGGCAAGCGT AAACGAAACC

20401 GCAGAAATCG AAAACGTGAA CGAAGCAGAC GACAGAATAT ATTTTCGAAA ATCGATTGCT

20461 TCGCTGCTGG GAGGAGGATA TCAGGCCATG AATCGACGTA GTGATTCTTA TTTTGCGGAT

20521 GATTCTTACG ATTATCATCA CAGACGTAAT CCCTTGGGCC GTCCCAAGTC TGCTTCCGTT

20581 TCGGTGCCGT CGCTTTGGCG TTCCTCCGAG ATCAGCGATC ATCCGTTCGC AGATTACCTA

20641 AAGTCTGCAA CGCCTCGTGG CGACGGGAAA AATTTCTACG GAACGCAAAA CGCAGTT**TAA**

20701 CGGCGACCAG CAAAGGAATG AAAATCAAAC AGCAGTGTAA ATCAAGAGTG GAGAATGTAT

20761 TGGCGGTGCA TGGCAGTGAG GAAATAGACT TATGTACACT GGAAACAAAC GTGGGAAGAG

20821 AATGGAATAG TATGTTGCAT TGCGTGTGAT TCCCATCATA CCAAATGTCA GTACCAAAGT

20881 CAATTGAATT AAATTGATAA CGTGTTACAC GTAAATGTAT TCCTTTCTGT GGCTCCCAAC

20941 CTTTACTTGC TAATTGCCCC CATTTTGACC CATTTCCAGT TGTGATTTAT TAAACGGGAA

21001 TATTTGTTTC GGCTTAGCTA GTCCTACGCA ATTATATGGA AATTAAATTG AATTATAATA

21061 TAGTCAAAAT TCTATGAAAC ATGATTGTGT ACAAGTAGTA CAACTAGGGT TATGACGCAG

23761 ACTACCAACA CCAATACTGT ATACATATAT CAACAAGCTT

//

LOCUS TuGIRKB genome sequence 5950 bp DNA 25-NOV-2003

BASE COUNT 1754 a 1049 c 1155 g 1992 t

ORIGIN

1

1381 AATTTTCAAC AGTGACTTTG GAGCTGGTAG TTAAGTTAGT CTGTAATATT AATGTGTTTT

1441 TGTAACAGCG TTATTTGCCT CGTAATTTAG CATAATAGCA TAATA**ATG**TC TCTTCGACGA

1501 CATTCTGGAG GCGGAGTCCA AATGTCGGCA AAATTGGCGG CACTGAGAGG AGAATCGATA

1561 GAAGGGGCAT CAATGTTAGA TACAACTACT ACACTTAGCA ATGGCGATCA TGAAGTGGTT

1621 CAAATAAAGA GCAAAACCAA ACAACCGGGT AGATTCATGA CAAAGACTGG TCATTGCAAT

1681 ATTCGCCGGG TAAATTGATT TACTCTGTAT AAGTCTTGAG ACTGATTTGA GGACAGGCTT

1741 AATATGGTTG GAATTAAGTT GCCGCAGTCT GGATACTAGT CCTGTAAACT CGTGTTCTAT

1801 GTTTACGCTA TTTTAATATA TCCCGTATTC ATCTAGATCT GGAAGATATC CTTAATTTCG

1861 TTCAACCATT CTGCCACTCG TACACAGCGG ACATTCTGTA GTACATCTTT CTCTTGTGTG

1921 ATTGTGGAGT ATTAACTCAC ACTTTGCGCT TTAGATATGA CAAAGAAACA GAAAAGAAAT

1981 TATAACTTTT CATTACTATT TAAAAATAAC CATATTTCTA GCTGAACATT CAACGTTATA

2041 TAATTCCACG TTTATTAAAT GAGTACGTAT ATATATATAT CGTGTGTATG TTTACCAGTG

2101 CTTTTTCAAC TTCTACCTTG TATAGTCTGC ACTACAAATG GGAACACGAT ACATGACGGA

2161 TATCTTTACC ACTTTGGTCG ATTTACGCTG GAAGTACAAC ATGATAATAT TCGTATTCGT

2221 GTATACTGCC GCCTGGTCTA TGTTTGGATT TCTTTGGTGG TTGGTTGCTT TCGTAAGGTT

2281 GGTGTCCTAT CGTGGTTCTG TGTCGTTGGA AATTTCCAAT CTCCATTTTG CTTTCCGTTC

2341 AAAGCCAATA TATGCCAATG TCCTTAATGG ATTGGCTTCC TACGAGTATT GATCGTTTCT

2401 TTACGATCTA TGTTCTTTCT GCAGAGGAGA CACGGACATA AATGTACACA ATGGCACCGA

2461 TTCTAGGAAA CCATGCGTGC AAAACGTTTA TTCCTACGCA ACCGCATTTC TTTTCTACAT

2521 AGAAACTGAG ACTACTATTG GGTATGGGAA AAGAGCTATG ACTGATCAAT GTCCAGAAGC

2581 TATATTGTTG TTCGTCATCC AGGTTAGTTA GGAGAGTATT TTAGGACCAT CAATTTGTTG

2641 TCCGATTAGC GTGAATATAC ATAAACTGTG ATGGGTTTTA ACCAAATCAT GAAATCAAGT

2701 TCAGTGTGGT CATAATAGAA GTCGTCTGTA AGGTAGAATG GAAGATTAAT TTTGTGTGGT

2761 GTAAAATATA GTTTCTCAAT AAAATCGTTT TGTACCATAG TATAGTGTTC ATTGTGTTAA

2821 TGTATGTAAA TGTATTTTAT TTACTTTCAG TCTCTCCTCG GAAGTATTGT GGATGCTTTT

2881 ATGGTCGGAT GTATATTTAT CAAACTTTCA CAGCCCAAAA ATCGTGCCGA GACCCTAGTT

2941 TTTAGCGAAC ACTGCATATT AACTCAGCGC GATGGAAAAT ATTGTCTCAT GTTCAGGTTT

3001 TTTTTTTCAA ACAGTTTCGA GCTTTTGCTG AAATTACAAG TAAAATCGTT CCAGACATGC

3061 CATTTGCAAT ATTTTTTTAA TTACAGAGTT GCTAATCTCA GAAATTCCCT GTTGATACAA

3121 TGTAAAATAC GGGCAAAGAT AGTCAAATCG CGACAAACGC TGGAAGGAGA ATTTATCGGT

3181 TTGCATCAGG ACGACATTAA TGTTGGTTTC GACACTGGTA AGCAGCATTA TCATACGTTT

3241 AACTGTGCCA TAGTCTGGTA CACATATATG TGCGGGGTTT GTGATCGTTG AAAATCAATA

3301 CATTCCTGAT ATCATATCGT CTTCATGAAC AAGAGGATCC ATGAATAATT TATTGATAGT

3361 CTTTCTCCCT TAATACACAC AAGAGCGTTG CAGATATTGT TTATCGTATA CGCATCGGAA

3421 ACCTTAATGA ATTAAACTAA ACCCTCCGGA AAACTGTTAC AGTACTCTAC TTCATAGCTT

3481 GTTTTTAAGC ACTCACATTT ACTGAACGAC ATTTTGCGAT GCATAAAAAT TTATTTAAAG

3541 ATACAGTAAT TGCATTCTTT AGGAATGCAA GCTACGTATC TTTATGATGT GTTCATAAAT

3601 TCCAAATCAG ACTTGGTCGT TTTAAAAAGT GGTATTGCGC GAAACTACCT TCTATTATAT

3661 GACAACTCCT CATTTTGCAG CCAAACGAAT GCAGTGAAAT CGTATTTTAC GCAAACGTAT

3721 TCATTTTACA GGTGCTGACA ATTTATTTTT GGTAACACCT CTTATCATTT GCCATGAAAT

3781 TGACCATCGA AGTCCCTTTT ACAACACTAA CGCTGAAGAT TTACAGAAAG ACAAGTTCGA

3841 AATAATAGTT ATATTAGAAG GCATGATAGA AAGCACAGGT ATGGTTTATT TCATGAATAA

3901 TTATGTCGTT AGTTTTTATT GTGAGAATGA ATATTTATTG CAGGAATGAT ATGCCAAGCT

3961 CGGACTTCTT ATCTCAATAC TGAGGTTCTG TGGGGCCATC GTTTTATGCC TGTGCTTTTT

4021 CACGCGCGTG ATCATTTCAG CGTCGATCAC TCGGAGTTTC ACACAACCTA CGAGGTTATT

4081 GAATCAAGTT CTTTTCACGA CAATACATCC TTATGCCGCG AAACACTGCC TTCAATATTT

4141 TTTCATTGCT ATTTTAGGTT CCCATGCCGA AACAGAGCAT GAGAAGATTT CATGATGCTC

4201 AGGTCCAAAA TAACACAAAG CAGTGGCATC CTGGTAGTTC GGGTTATGTT GGTAACACTG

4261 CAGCA**TAA**AT GTCTTCCGCT TTCACGCGTC TTAAATTGTC TCATACTGTA TATGCAGAAG

4321 TGTGAGAGTA TTTTTAAGGT TCATGAATTA TGAACCAGAT TGCTATAATG ATTATCTTTG

4381 TAGGATTTAC AGTCGAAGGC AACAAAGGCT AGGGAGTTAA TTGGTCTATT TAAGGACGAT

4441 GCTCATCGAC CTCGCTGCAC CTTGGGTCAT AATTTTTTAC TTAACGATCT GATCTTCTTT

4501 AATAACATTA ATAATGACTT GATGATAAAT GAGGCATCTT TGCAAATGAG CTAACTGAAC

4561 CGAGTGCGGC CGCATTGGTG ATTTCAACCT TACACGGTTA ACAGTGACCT TTCACGAGTG

4621 TTCCGTATTT ATACGCCTAG TTCACAGTTG GTGTTTTAAC GGTACTGCAC AACATGTTCA

4681 TGCTTGCGAT TATTCCAACC TCACATAATT TAATCAAATG CTTTCCAACC TACTCCAATT

4741 CCCCACGTTT CCTCAAATCC TGTCTGCAAT GGTTTCAATA ATTCAAACTT GGCCAACGGA

4801 TTTTTATTAG CAATTTTCTG ACATAGATAC AAAATATAAA ACCAACAGTA AGTATATATA

4861 GTAGGAACCC ACTGCGGCAG GTTTATCTGC GCGTGAGCAA GTGCAGTTAT TAAAATAAAT

4921 CGACAATAAA TAACAGTGTG CAAAATGACG GATAGTAAAA ATATTGACAC TTCTAAATAC

4981 TTTTATAATG ATCACTGTAC AGCAATATAT AGATATAATC GAGAAAATGT ATTTTGTGAT

5941 ACCGGTCGAC

//
