# Supplementary material for: Ancient intron insertion sites and palindromic genomic duplication evolutionally shapes an elementally functioning membrane protein family
Source: BMC Evol Biol. 2007 Aug 20;7:143. doi: 10.1186/1471-2148-7-143 (PMC1999503; doi:10.1186/1471-2148-7-143)
Supplement: Additional file 2 — TuIRKA, TuGIRKAa and TuGIRKB cDNA sequences. The data provided represent the IRK cDNA sequences inserted in the vectors used in the present experiment. [file 1471-2148-7-143-S2.doc]

LOCUS TuIRKA cDNA 3891 bp DNA 22-OCT-2003

BASE COUNT 1341 a 739 c 721 g 1090 t

ORIGIN

1 GAATTCGGCA CGAGCGTAAA AAAAATCACT GTATTATAAA TAGGATTGAT TCCGGACCTT

61 GGCCTGGAAT AAACTGAATT ATTGAGCAAC TGAGAGGCGC CAGAGAACTT TGATATAGCA

121 CTGCTATTAC GTTAAACTGC TCTGCGTAAT TCAACAAGAG GAAACTGTGT TGAGTCAGCG

181 TGCTGCCGTA CAACGCGTAT GCGGTACACC ATTGATACAG TTAGTAAAGT ACTGCATCAC

241 ACTAGCACCA GCAAGGCATT AGTTAACGCT CGAAAAAGCC AGATAGGTGA ACATTGCATC

301 GTGAAAGAAC ACAGGATGGA TTTATCGCAG AAATCTCCAA GATTGGGAAG AAATATAATG

361 GATACAGTCA GTAACAGAAT ACCCATCGAA TCAAGAGATG CATACATTCA AATATCAAAT

421 GACAGCCAAG ATCTAGATCC AACAACAGAT GACACATTGA GAAATATGAG TAACAGATCA

481 TCAAACCCAA TTATCATGAC ATGCTCGGGC CTCAATGACA ACAGCAGTAA CAGCAGTAGC

541 CTACAAAGAA TTGTCTACGG CAGCGAGTAT ATAAACGATA TGCATGCATC CAGCCGATCT

601 CTAGCAAGTC GGAATATGCA CAGGAGGAAG AACTGCAGAT TCATCAAGAA ATCTGGACAT

661 TGCAATGTCG GCCACACGAA CGTAAACAAG AAACCGCAAA GATTCTTGGC AGATATTTTC

721 ACCACATGCG TCGATTTGAA ATGGAGATGG AATCTTTTGT TATTCAGTGC TGCTTTTATA

781 CTCAGCTGGC TTTTTTTCGG ATTTATTTAT TGGATTATAT CCTACATCCA CGGCGATTTC

841 TCGACAAACC AAACAGATAT TCAATGTATC AGTAATCTTG AGTCATCTTC CCCATTCACA

901 TCAACATTCC TATTTTCACT GGAAACTCAG ACGACAATTG GTTATGGATC GCGAGCTGTT

961 ACAGAAGAAT GTCCTATGGC TATTCTCACA GTAGTTATCC AATCAGTATA CGGCTGCATT

1021 ATCGATGCAT TTATGATTGG TCTGATAATG GCCAAGATAT CGAGGCCAAA GAAGAGGGCT

1081 GAAACACTGC TGTTCAGTAA GAAGGCTGTC ATCAGTATGA GAGATGGTCA ATTGTGCTTA

1141 ATGGTGAGAG TGGGCAACTT GAGGAAAAGT CACTTGGTGG AAGCAACAAT CAGAATGCAA

1201 TATATTTATT CGCGGGAAAC GATCGAAGGA GAGTTTATAC CCCTTGAACA AGTCGATCTT

1261 CATCTGGATT TGAAGAATGA TTCTGACAGA TTATTCCTAG TGACACCACA AACCATTTGT

1321 CATCCAATCG ACTCCGATAG TCCACTCTAC CATTTAAATA AAGAATCGTT AAAAGAAGCA

1381 AATTTTGAGG TCATTGTAAT ATTGGAGGGA ATGGTTGAAG CAACAGGCAT GACTACTCAA

1441 GCACGCGCGT CGTATGTTCC AGATGAGATA ATGTGGGGAC ATCGATTTGA AAACGTAATT

1501 ACGTTTTCAA GAACGAGCAG ATATAACGTC GATTTTCGAA AGTTCGATAG GTCATACGAA

1561 GTACCGGAAA CACCTAAATG CAGCTCTAAA TATCTGCAAG ATATGCAAAA TGCAGACACC

1621 GTATCAGAGA GAATAATGTT GCAAGATAAG GAACAAAATC GGAAGAATAC ATCTAAAGAC

1681 GCTACTAGAG AAGACTTCAG TGTATCAGAT GGACGAGACT CAGGATACAC AGCACCAGAA

1741 GATTATCAAG CGACAGACAG CAACAAAACA CTAAATAGTA CATCGACCTG CGGAAGCTCT

1801 TACATCAATC ATGATACCGA AAACCGAAGG CGAAGTTGGA CAAAGAAGGC GATCGAGGAC

1861 ACAATGAACA AAAATTCGAA TCAATACGAA AGTACAAACA TGGATAAATG TCAGACATCT

1921 TCAGATATCT TGCCAAACAT GAAACAAGAT ATGCAGATAA AGACCAATCG TAACAGCGCT

1981 GACATGACAG CCCAAATGAC CCTCACTCAT AACGAGAAAA ACGAAGCTTT GAATAGTACA

2041 GCTCGACCTC GAAGTGGTAA TGTACGCATT TCAACAGCTA TCCCGCTCGA TATATTAAAA

2101 ATATCAACAG TCACAGTCCC TGAGGAGGTC GCCAAGAACA AAAATGGAAC GAGTTTCATA

2161 GGCGATCAAA ATCCTAAATC GAATCTCAAA GAACTGCAGA ACAATAACGA GTCAAAAAAA

2221 TCAACTGAAA TTCTTAGCGG AGGAAGAAGT TCATTGCTTG TTCCAAGACA AAAGAAGACG

2281 CAGACCGAGC ACGTGAATAT CTTAGCGACG ACTAGACAAG ACGATATCGT TCAGCAAATA

2341 AAAGAAGACC CCTTAGAAAA TAAATCTTTA CTAGACAATA CTTCGCCAGT AAAAAAAATT

2401 TGTGAGGTCG AAATGCCTGA AATCAAAATC GAGTGAACAC TGTACATGAG CAATAAGAGT

2461 CTATACCTAC CTACATAACA AGGCATTACG TTTCTACGTG GTGTTAACTG AGAACTGCAG

2521 TGCTACCTGA GTTTGCTTAG TGCTAACCAC GAAACCTGCC ATGCAGATCC TTTATCTCAG

2581 TGATTGGAGA ATTCTCGTCT TTGAAAACTT TAAAACCTAC TGTATTGTAT ATAGTATATA

2641 GACTTTATTA ATTAATTACT GAATTTATCC GAAATTACTT CTTGACTACC CAAAGTCAAT

2701 TGTTTTTACT TCTTCCAATC TAAATAAAAT ATTTATTGAA ATGGTAATTA GGAAATTGAT

2761 CTTTTGCCCC CCGAATAACA GTATGTATCG GACTAAGAAA ATTACTTACG GGCTCACTTA

2821 ACAAGTCATG TAAACATAAT TAATCCACTT CCAGTCTGAA ACGCAAATGA ACTAAATTTT

2881 TAATTTTGTT ATTGAATTGT CCATTAAAAC TATATATTTA TTATTACAAT CGAAATATTA

2941 GGTGCTTCAA AATTAATATA TAATTATGAA GTATCATACT GTTATTTGTA ATAATTGTTT

3001 TTAGTGAATT TTTCTCGCTT TTCAATGACG CTTTTGCTTT TTTCCGTTGT TTCATTTTAT

3061 TTCATTACAG GGGGTAAGTA ATAATCATAC ATATTTCATT GTAGAACTTA TTGTAAACAT

3121 ACTAAGTCAT CGCTGTCCCA ATGAATATTT AAGTAAGGGT CACACCATAG ATCAGACGAT

3181 GCAATTACGA CGAGCAATGT AAACAACAGA AATTCCCCGT TTTCCTGCTC CGAAATTTTC

3241 GTTGGTACTG TGGACGTAGC CCCGCTGTTA CACTTAAGTA ATTTCTAGTG TGAATAAATA

3301 TATGAGTAGG AATATATTTA ATCAAAAACT TGCATCGTCA TCTGATACAA AAGGATTTAA

3361 TGACAGCGCT CCCTACGCAA GCAGAGCCAA CCGTAGATAG TTTGGCTCCT CTGGAATAAC

3421 TACTTTAGGC ATTATTCCTC GTAGCCAAAT AGTGGAATAA ATTTATGGAC AAAATATCTT

3481 GATCCTTCTT GTTTATCATA CGATTTATCA AGCATTATGC AATTTGTGAA TTAGCATAGT

3541 GAAATTCGAA TAACGCGTAT GCTTTTACCA GTATTTGTCG TTTTAGTTCC ATTGCTTAAG

3601 CACGTTTCAA TCAAATATAT CCCATGTAGT ATGTCTATTC ATAACATTTG CAAATTATTT

3661 TTCTTCCTTT GCCGTCATTC ATGCATATGT GTGAATCATT GTTGTAAATA TATTAATTGC

3721 TTTTATTTTA TTACCATGCG TTGTATGACG TTATTTTACA CGAAATGAAT TTTGGCAATA

3781 GCCAATCGCC AATGAAATAC CATGTCGACT AATCCTTTAA CATTTACTCG CTGTTTCGTC

3841 ATTGTGGTGG CGATAGTCAC AAATTTGAAC AAAATAGAAA TCTACAGCCT A

//

LOCUS TuGIRKAa cDNA sequence 2386 bp DNA 08-NOV-2002

BASE COUNT 773 a 479 c 545 g 589 t

ORIGIN

1 AGGAATTCGG CACGAGAGAA CATGGAAATG AAAATAATAG TTGTTTAAAT AGTAACGTAG

61 TGGAAGATAA ACGTTTGAAA CGCACAATAA ACAATAACCA ACGAATCGCT TGGAATATAT

121 TCATTGGTTA TCGAAGGAAT GGGTGTAATC AGCAACGTAA GTCCGGATGT GACCAATAAA

181 TGGAGAACAG ATTCACAAGT TTTGCCGGCT TTAGGAAACA GCAAAGCAAG GGACACGCTC

241 TTTCAAGAGA AGATGGTTCG CACACATTCA AGAAGACCAT CCTGCGTTCC AGCGATCACA

301 CGGGATACCC CAGGTGGCGT GAAACGACCG ACCAAACGAA GACGACAGAC GAGGTTTGTT

361 ACGAAAAATG GTCACTGTAA CGTCAGACAC GGCAATGTTG AAGATCGGTC AAGGTATTTA

421 TCCGATCTCT TCACAACCCT GGTCGACCTA GAGTGGAGGT ACAACGTCAT GATTTTTATA

481 TCGACCTACA CAATAACGTG GCTGGTGTTT GCCTTCGTTT GGTGGTTCAT ATCATTTTGC

541 AGGAACGACC TGAATCTCGA TGTCAAAAAC CAAACGGTTT GTGTGATCGG TATCAAATCA

601 TTTACATCGG CTTTTTTATT CTCAATAGAA ACTCAGGTCA CTATCGGCTA TGGGACAAGA

661 GCAATAACGG AACATTGTCC AGAGGCTATC ATATTACTTC TGATTCAAAG TCTGCTGGGA

721 TCTATTGTCG ACGCATTCAT GGTTGGATGC ATGTTTGTCA AAATTTCACA GCCAAAGAAG

781 CGAGCTGAGA CGATAATGTT TAGCCATAAA GCCGTTATGT CTCTAAGAGA CGGTCAGATG

841 TGCCTAATGT TCAGGGTCGG TGATTTAAGA AATTCGCACA TAGTCGAAGC CCAGATTCGG

901 GCAAAATTAA TAAAGTCGAG ACAGACGCAA GAAGGTGAAT TCATGGCACT CGATCAAACC

961 GATTTTAACG TTGGATTCAC CACAGGAGCG GATCGACTCT TTCTGGTGAC TCCTCTTATA

1021 ATTTGTCACA TTATCGACGA AAAATCACCG TTTTGGGAGA TGTCTGCAGA AGACCTGATA

1081 AACGAAGAGT TTGAAATAGT TGTTATTCTC GAAGGAATGG TGGAAGCTAC AGGAATGACC

1141 TGTCAAGCTA GAAGTTCATA CGTTGAAGAT GAAGTATTAT GGGGTCAAAG GTTCATGCAA

1201 GTGCTCATGT TGGAGAAAGG GTACTTTGAG GTGAACTATA ACAATTTTCA CGATACCTTC

1261 GAAGTATCCT CGCCGACTGC TAGTGCAAAA GAACAAGCAG AAGAGAGAAT AAAGCAACGT

1321 TTGAATGAAG CTAACAATTC CCCCGGTGCC TTAAGTATGC ATAGAACACT TCCACGATCG

1381 CCCAGAGTAC AGGAAAGCGC GAGTTTGAGC TCGTCGTTCC CAACCCCGAC GGTGCGAAGG

1441 AAAAAAACCT CAATTGCAAA CAACTGTTAC CCGGACGAAG TGGAAGAAAA CAATAAAAGC

1501 GATTCCCCTG GTATCGAAAA AACCAACGTC CAATCGAACG AAAATAATAA CAATAGTTTG

1561 CAGCCAGTAC TTGCGAGTTA TCCCGTGCAC AGCACGCTCG GTCAGCAACA GGCATCGAGC

1621 CAGAGCGAGG CCGACTTGCT GCGATTGAAC AAACGCTTGG CAAGCGTAAA CGAAACCGCA

1681 GAAATCGAAA ACGTGAACGA AGCAGACGAC AGAATATATT TTCGAAAATC GATTGCTTCG

1741 CTGCTGGGAG GAGGATATCA GGCCATGAAT CGACGTAATG ATTCTTATTT TGCGGATGAT

1801 TCTTACGATT ATCATCACAG ACGTAATCCC TTGGGCCGTC CCAAGTCTGC TTCCGTTTCG

1861 GTGCCGTCGC TTTGGCGTTC CTCCGAGATC AGCGATCATC CGTTCGCAGA TTACCTAAAG

1921 TCTGCAACGC CTCGCGGCGA CGGGAAAAAT TTCTACGGAA CGCAAAACGC AGTTTAACGG

1981 CGACCAGCAA AGGAATGAAA ATCAAACAGC AGTGTAAATC AAGAGTGGAG AATGTATTGG

2041 CGGTGCATGG CAGTGAGGAA ATAGACTTAT GTACACTGGA AACAAACGTG GGAAGAGAAT

2101 GGAATAGTAT GTTGCATTGC GTGTGATTCC CATCATACCA AATGTCAGTA CCAAAATCAA

2161 TTGGATTAAA TTGATAACGT GTTAAACGTA AATGTATTCC TTTCTGTGGC TCCCAACCTT

2221 TACTTGCTAA TTGCCCCCAT TTTGACCCAT TTCCAGTTGT GATTTATTAA ACGGGAATAT

2281 TTGTTTCGGC TTAGCTAGTC CTACGCAATT ATATGGAAAT TAAATTGATT TATAATATAG

2341 TCAAAATTCT ATGAAAAAAA AAAAAAAAAA AAAAAAAAAA CTCGAG

//

LOCUS TuGIRKB cDNA sequence 2073 bp DNA 22-OCT-2003

BASE COUNT 669 a 394 c 414 g 595 t

ORIGIN

1 AGGAATTCGG CACGAGGCGA TATTTGCCTC GTAATTTAGC ATAATAGCAT AATAATGTCT

61 CTTCGACGAC ATTCTGGAGG CGGAGTCCAA ATGTCGGCAA AATTGGCGGC ACTGAGAGGA

121 GAATCGATAG AAGGGGCATC AATGTTAGAT ACAACTACTA CACTTAGCAA TGGCGATCAT

181 GAAGTGGTTC AAATAAAGAG CAAAACCAAA CAACCGGGTA GATTCATGAC AAAGACTGGT

241 CATTGCAATA TTCGCCGGTC TGCACTACAA ATGGGAACAC GATACATGAC GGATATCTTT

301 ACCACTTTGG TCGATTTACG CTGGAAGTAC AACATGATAA TATTCGTATT CGTGTATACT

361 GCCGCCTGGT CTATGTTTGG ATTTCTTTGG TGGATGGTTG CTTTCGTAAG AGGAGACACG

421 GACATAAATG TACACAATGG CACCGATTCT AGGAAACCAT GCGTGCAAAA CGTTTATTCC

481 TACGCAACCG CATTTCTTTT CTACATAGAA ACTGAGACAA CTATTGGGTA TGGGAAAAGA

541 GCTATGACTG ATCAATGTCC AGAAGCTATA TTGTTGTTCG TCATCCAGTC TCTCCTCGGA

601 AGTATTGTGG ATGCTTTTAT GGTCGGATGT ATATTTATCA AGCTTTCACA GCCCAAAAAT

661 CGTGCCGAGA CCCTAGTTTT TAGCGAACAC TGCATATTAA CTCAGCGCGA TGGAAAATAT

721 TGTCTCATGT TCAGAGTTGC TAATCTCAGA AATTCCCTGT TGATACAATG TAAAATACGG

781 GCAAAGATAG TCAAATCGCG ACAAACGCTG GAAGGAGAAT TTATCGGTTT GCATCAGGAC

841 GACATTAATG TTGGTTTCGA CACTGGTGCT GACAATTTAT TTTTGGTAAC ACCTCTTATC

901 ATTTGCCATG AAATTGACCA TCGAAGTCCC TTTTACAACA CTAACGCTGA AGATTTACAG

961 AAAGACAAGT TCGAAATAAT AGTTATATTA GAAGGCATGA TAGAAAGCAC AGGAATGATA

1021 TGCCAAGCTC GGACTTCTTA TCTCAATACT GAGGTTCTGT GGGGCCATCG TTTTATGCCT

1081 GTGCTTTTTC ACGCGCGTGA TCATTTCAGC GTCGATCACT CGGAGTTTCA CACAACCTAC

1141 GAGGTTCCCA TGCCGAAACA GAGCATGAGA AGATTTCATG ATGCTCAGGT CCAAAATAAC

1201 ACAAAGCAGT GGCATCCTGG TAGTTCGGGT TATGTTGGTA ACACTGCAGC ATAAATGTCT

1261 TCCGTTTTCA CGCGTCTTAA ATTGTCTCAT ACTGTATATG CAGAAGTGTG AGAGTATTTT

1321 TAAGGTTCAT GAATTATGAA CCAGATGCTA AAATGATTAT CTTTGTAGGA TTTACAGTCG

1381 AAGGCAACAA AGGCTAGGGA GTTAATTGGT CTATTTAAGG ACGATGCTCA TCGACCTCGC

1441 TGCACCTTGG GTCATAATTT TCTACTTAAC GATCTGATCT TCTTTAATAA CATTAATAAT

1501 GACTTGATGA TAAATGAGGC ATCTTTGCAA ATGAGCTAAC TGAACCGAGT GCGGCCGCAT

1561 TGGTGATTTC GACCTTACAC GGTTAACAGT GACCTTTCAC GAGTGTTCCG TATTTATACG

1621 CCTAGTTCAC AGTTGGTGTT TTAACGGTAC TGCACAACAT GTTCATGCTT GCGATTTTTC

1681 CAACCTCACA TAATTTAATC AAATGCTTTC CAACCTACTC CAATTCCCCA CGTTTCCTCA

1741 ACTCCTGTCT GTAATGGTTT CAATAATTCA AATTTGGCCA ACGGATTTTT ATTAGCAATT

1801 TTCTGACATA GNATACAAAA TATAAAACCA ACAGTAAGTA TATATAGTAG GAACCCACTG

1861 CGGCAGGTTT ATCTGCGCGT GAGCAAGTGC AGTTATTAAA ATAAATCGAC AATAAATAAC

1921 AGTGTGCAAA ATGACGGATA GTAAAAATAT TAACACTTCT AAATACTTTT ATAATGATCA

1981 CTGTACAGCA ATATATAGAT ATAATCGAGA AAATGTAAAA AAAAAAAAAA AAAAAAAAAA

2041 AAAAAAAAAA AAAAAAAAAA AAAAAAActc gag

//
