# Supplementary material for: Ancient intron insertion sites and palindromic genomic duplication evolutionally shapes an elementally functioning membrane protein family
Source: BMC Evol Biol. 2007 Aug 20;7:143. doi: 10.1186/1471-2148-7-143 (PMC1999503; doi:10.1186/1471-2148-7-143)
Supplement: Additional file 3 — Scematic illustration of TuIRKA, TuGIRKAa and TuGIRKB genomic sequences. The data provided the schematic illustration of the intron-exon structures of Halocynthia IRK gene genomic sequences. And the obtained genomic clones used for sequencing. [file 1471-2148-7-143-S3.doc]

# Illustration of TuIRKA genome sequence

**TGA**

**ATG**

**5’**

TuIRKA11 genome clone

TuIRKA14 genome clone

**TuIRKA genome**

**TuIRKA11 genome clone**  derived from Genome library pool No.1

withTuIRKA Probe No.1 (cDNA#1059~1927)

**TuIRKA14 genome clone** derived from Genome library pool No.6

with TuIRKA Probe No.2 (cDNA#2025~2594)

**ATG** cDNA #199~ / TuIRKA genome #4832~

**TGA** cDNA #2434~ / TuIRKA genome #15992~

**M1** cDNA #761~828 / TuIRKA genome #11766~11791, 12286~12328

**H5** cDNA #907~987 / TuIRKA genome #12407~12440, 12924~12940

**M2** cDNA #1000~1064 / TuIRKA genome #12983~13047, 13756

|  | TuIRKA genome | cDNA |
| --- | --- | --- |
| Exon1 | ＃4649〜5010 | ＃16〜377 |
| **Exon2** | ＃8026〜8124 | ＃378〜476 |
| **Exon3** | ＃9511〜9672 | ＃477〜638 |
| **Exon4** | ＃11645〜11791 | ＃639〜785 |
| **Exon5** | ＃12286〜12440 | ＃786〜940 |
| **Exon6** | ＃12924〜13047 | ＃941〜1064 |
| **Exon7** | ＃13756〜13884 | ＃1065〜1193 |
| **Exon8** | ＃14029〜14224 | ＃1194〜1389 |
| **Exon9** | ＃14552〜14713 | ＃1390〜1551 |
| **Exon10** | ＃14797〜14918 | ＃1552〜1673 |
| **Exon11** | ＃15027〜15255 | ＃1674〜1902 |
| **Exon12**  **Unkown insert 1**  **Unkown insert 2** | ＃15461〜17462  ＃15997~16005  ＃16357~16362 | ＃1903〜3890 |

# Illustration of TuGIRKAa genome sequence

**TAA**

**ATG**

**XhoⅠ**

**5’**

**TuGIRKAa genome**

**TuGIRKAa3 genome clone**

**TuGIRKAa9 genome clone**

**TuGIRKAa3 genome clone** Derived from Genome library pool No.4

With TuGIRKAa Probe No.1 (cDNA#4~938)

**TuGIRKAa9 genome clone** Derived from Genome library pool No.9

With TuGIRKAa Probe No.3 (TuGIRKAa genome #14609~14824)

**ATG** cDNA #139~ / TuGIRKAa genome #8241~

**TAA** cDNA #1975~ / TuGIRKAa genome #20698~

**M1** cDNA #466~534 / TuGIRKAa genome #12349~12417

**H5** cDNA #613~663 / TuGIRKAa genome #14152~14175, 14390~14416

**M2** cDNA #688~756 / TuGIRKAa genome #14441~14494, 14932~14946

|  | TuGIRKAa genome | cDNA |
| --- | --- | --- |
| Exon1 | ＃8119〜8335 | ＃17〜233 |
| **Exon2** | ＃10470〜10589 | ＃234〜353 |
| **Exon3** | ＃12237〜12425 | ＃354〜542 |
| **Exon4** | ＃14082〜14175 | ＃543〜636 |
| **Exon5** | ＃14390〜14494 | ＃637〜741 |
| **Exon6** | ＃14932〜15044 | ＃742〜854 |
| **Exon7** | ＃15741〜15891 | ＃855〜1005 |
| **Exon8** | ＃16637〜16763 | ＃1006〜1132 |
| **Exon9** | ＃18402〜18499 | ＃1133〜1230 |
| **Exon10** | ＃19345〜19428 | ＃1231〜1314 |
| **Exon11** | ＃19514〜19608 | ＃1315〜1409 |
| **Exon12** | ＃20133〜21076 | ＃1410〜2353 |

# Illustration of TuGIRKB genome sequence

**TuGIRKB2 genome clone** Derived from Genome library pool No7

With TuGIRKB Probe No.2 (cDNA#4~295) and No3 (cDNA#1271~2069)

**ATG** cDNA #55~ / TuGIRKB genome #1486~

**TAA** cDNA #1252~ / TuGIRKＢ genome #4266~

**M1** cDNA #339~405 / TuGIRKB genome #2206~2272

**H5** cDNA #489~540 / TuGIRKB genome #2503~2554

**M2** cDNA #564~633 / TuGIRKB genome #2578~2602, 2851~2895

|  | TuGIRKB genome | cDNA |
| --- | --- | --- |
| Exon1 | ＃〜1452〜1689 | ＃〜1〜258 |
| **Exon2** | ＃2126〜2277 | ＃259〜410 |
| **Exon3** | ＃2425〜2602 | ＃411〜588 |
| **Exon4** | ＃2851〜2996 | ＃589〜734 |
| **Exon5** | ＃3087〜3217 | ＃735〜865 |
| **Exon6** | ＃3732〜3878 | ＃866〜1012 |
| **Exon7** | ＃3944〜4074 | ＃1013〜1143 |
| **Exon8** | ＃4158〜5030 | ＃1144〜2026 |
