# Supplementary material for: Ancient intron insertion sites and palindromic genomic duplication evolutionally shapes an elementally functioning membrane protein family
Source: BMC Evol Biol. 2007 Aug 20;7:143. doi: 10.1186/1471-2148-7-143 (PMC1999503; doi:10.1186/1471-2148-7-143)
Supplement: Additional file 4 — Halocynthia IRK genome Intron-Exon Junctions. (A) Elementary IRK, TuIRKA genome. (B) G-protein activated IRK, TuGIRKAa genome. (C) G-protein activated IRK, TuGIRKB genome. For the explanation of colored characters, see the legend of Fig. 2 in the original paper. [file 1471-2148-7-143-S4.pdf]

A. TuIRKA Intron-Exon Junctions

	Donor	*Intron*	Accepter	
#366	GTC AGT AAC AGgtgagaaatc	------	ctttcaacagA ATA CCC ATC	388
	Val Ser Asn Arg		Ile Pro Ile	
465	ATG AGT AAC AGgtgatgagaa	------	ttgttacaagA TCA TCA AAC	487
	Met Ser Asn Arg		Ser Ser Asn	
627	AAG AAC TGC AGgtaagtcaat	------	atcatttcagA TTC ATC AAG	649
	Lys Asn Cys Arg		Phe Ile Lys	
774	TTT ATA CTC AGgtaataaaat	------	gtttaacagC TGG CTT TTT	796
	Phe Ile Leu Ser		Trp Leu Phe	
930	ACG ACA ATT Ggtgagaaatt	------	cgtataacagGT TAT GGA TCG	952
	Thr Thr Ile Gly		Tyr Gly Ser	
1053	AAG ATA TCG AGgtaccgaatg	------	ttacgttcagG CCA AAG AAG	1075
	Lys Ile Ser Arg		Pro Lys Lys	
1182	GCA ACA ATC AGgtaagctcaa	------	attttacagA ATG CAA TAT	1204
	Ala Thr Ile Arg		Met Gln Tyr	
1377	GCA AAT TTT GAGgtgagcttgg	------	gccctttcag GTC ATT GTA	1398
	Ala Asn Phe Glu		Val Ile Val	
1539	AAG TTC GAT AGGgtataaaaac	------	atttcaacag TCA TAC GAA	1560
	Lys Phe Asp Arg		Ser Tyr Glu	
1662	AAG AAT ACA TCgtaagttttg	------	cttgtcacagT AAA GAC GCT	1684
	Lys Asn Thr Ser		Lys Asp Ala	
1890	AGT ACA AAC ATGgtaaatattc	------	ttaattccag GAT AAA TGT	1910
	Ser Thr Asn Met		Asp Lys Cys	

B. TuGIRKAa Intron-Exon Junctions

	Donor	*Intron*	Accepter	
#222	AAA GCA AGG GAgtgagtaaaa	------	tattttccagC ACG CTC TTT	244
	Lys Ala Arg Asp		Thr Leu Phe	
342	CGA CAG ACG AGgtaaagattg	------	cattgaacagG TTT GTT ACG	364
	Arg Gln Thr Arg		Phe Val Thr	
531	TCA TTT TGC AGgtaggaacat	------	gttgttccagG AAC GAC CTG	553
	Ser Phe Cys Arg		Asn Asp Leu	
624	ATA GAA ACT CAGgtgagtaaca	------	ctacttttag GTC ACT ATC G	647
	Ile Glu Thr Gln		Val Thr Ile Gly	
729	GAC GCA TTC ATGgtaatgattc	------	cttattacag GTT GGA TGC ATG	754
	ASp Ala Phe Met		Val Gly Cys Met	
843	CTA ATG TTC AGgtatatatcc	------	ttttgcacagG GTC GGT GAT	865
	Leu Met Phe Arg		Val Gly Asp	
993	CGA CTC TTT CTAgtgagttcat	------	attattcaag GTG ACT CCT C	1106
	Arg Leu Phe Leu		Val Thr Pro Leu	
1122	GAA GCT ACA Ggtaagttgac	------	ttaatcccagGA ATG ACC TG	1143
	Glu Ala Thr Gly		Met Thr Cys	
1220	G TAC TTT GAGgtaagtgatt	------	ttatttgtag GTG AAC TAT A	1241
	Gly Tyr Phe Glu		Val Asn Tyr Asn	
1304	G AGA ATA AAGgtagtatata	------	ttttttacag CAA CGT TTG A	1325
	Glu Arg Ile Lys		Gln Arg Leu Asn	
1399	CG AGT TTG AGgtaagaagtt	------	ataattacagC TCG TCG TTC	1420
	Ala Ser Leu Ser		Ser Ser Phe	

***C.* TuGIRKB Intron-Exon Junctions**

Donor	Intron	Accepter
#248 T ATT CGC CGGgtaaattgat	------	ccttgtatag TCT GCA CTA 268
Asn Ile Arg Arg		Ser Ala Leu
400 CT TTC GTA AGgttggtgtcc	------	ctttctgcagA GGA GAC ACG 420
Ala Phe Val Arg		Gly Asp Thr
578 C GTC ATC CAGgttagttagg	------	ttactttcag TCT CTC CTC 598
Phe Val Ile Gln		Ser Leu Leu
724 TC ATG TTC AGgttttttttt	------	ttaattacagA GTT GCT AAT 745
Leu Met Phe Arg		Val Ala Asn
855 TTC GAC ACT Ggtaagcagca	------	cattttacagGT GCT GAC AA 876
Phe Asp Thr Gly		Ala Asp Asn
1002 GAA AGC ACA Ggtatggttta	------	tttattgcagGA ATG ATA TG 1023
Glu Ser Thr Gly		Met Ile Cys
1133 A ACC TAC GAGgttattgaat	------	gctattttag GTT CCC ATG 1153
Thr Thr Tyr Glu		Val Pro Met

Serial number of nucleotide in respective cDNAs.
