# Supplementary material for: Ancient intron insertion sites and palindromic genomic duplication evolutionally shapes an elementally functioning membrane protein family
Source: BMC Evol Biol. 2007 Aug 20;7:143. doi: 10.1186/1471-2148-7-143 (PMC1999503; doi:10.1186/1471-2148-7-143)
Supplement: Additional file 6 — Alignment of IRK AA sequences. Alignment of 7 tunicate, 6 Caenorhabditis, and 7 bacterial IRK AA sequences. For the explanation of colored characters, see the legend of Fig. 6 in the original paper. [file 1471-2148-7-143-S6.pdf]

Alignment: 20IRK HR-CI-CE-CB-BAC

```
                     ....|....| ....|....| ....|....| ....|....| ....|....| ....|....| ....|....| ....|....| ....|....| ....|....| ....|....| ....|....|
                         5         15         25         35         45         55         65         75         85         95        105        115
HR-TuIRKA            MRYTIDTVSK VLHHTSTSKA LVNARKSQIG EHCIVKEHRM DLSQKSPRLG RNIMDTVSNR IPIESRDAYI QISNDSQDLD PTTDDTLRNM SNRSSNPIIM TCSGLNDNSS NSSSLQRIVY
CI-IRK               ----MDNMG- ---------- ---LRRGRVE NYGLP----- GLRHSSLRNG ----CTLRNI SELPSVSGSV EIHNQS---- --------NM NNLR------ -------KVS NSSSLQGMMT
HR-TuGIRKAa          ---------- ---------- ---------- ---------- ---------M GVISNVSPDV TNKWRTDSQV LPALGN---- ---------- ---------- --SKARDTLF QEKMVRTHSR
CI-GIRKA/G           ---------- ---------- ---------- ---------- ---------- ---------- ---------- ---------- ---------- ---------- ---------- ----------
HR-TuGIRKB           ---------- ---------- ---------- ---------- ---------- ---------M SLRRHSGGGV QMSAKL---- ---------- ---------- --AALRGESI EGASMLDT--
CI-GIRKB/G           ---------- ---------- ---------- ------MEME HLKPEAEAKS LSVPGTPNTM TTRRTS--VV QMSAKL---- ---------- ---------- --AALRGETV REEVRSNSDL
CI-IRK/AR            ---------- ---------- ---------- ---------- ---------- ---------- ---------- ---------- ---------- ---------- ---------- ----------
CB-Irk2/AR           ---------- ---------- ------MDPS SSRLVSFNQK ITVRREIESD DNETFILKMR -NGRHRKTTV AMSPSVT--- ---------- ---------- ----DLLTAQ RMLDIEEPLV
CE-Irk2/AR           ---------- ---------- ------MS-S ASTPPSKSVQ FSFR---ETD N---LITKAS ENGRRRKTTV AMSPSVT--- ---------- ---------- ----DIFTAQ RMLDIEEPLV
CB-Irk1/AR           ---------- ---------- ------MTLS VPDCAEVNRL RMSNHRKMSL GNAPLIANGR PSPPP--RRT SLAESIR--- ---------- ---------- ----TLAFTA RRN--SSPLY
CE-Irk1/AR           ---------- ---------- ------MLLD T-NTITFPFF VNGLFRFQDS ANSLVIQTKM PSCAHSISAL SFLESIR--- ---------- ---------- ----TLAFTA RRN--SSPLY
CB-Irk3/AR           ---------- ---------- ---------- ---------- -------MSL AEELERLSGE NGAKKELNGW RLSSSAP--- ---------- ---------- ----ELGVQ- ----------
CE-Irk3/AR           ---------- ---------- ---------- ---------- -------MSL AEELERLSGE NGAKKELNGW RLSSSAP--- ---------- ---------- ----ELGVQ- ----------
Nos-K-channel-like   ---------- ---------- ---------- ---------- ---------- ----MKFQLK RLAKKRPDR- ---------- ---------- ---------- ---------- ----------
Nos-Kef-type         ---------- ---------- ---------- ---------- ---------- ----MKFRLK RLSRKKQQRL IL-------- ---------- ---------- ---------- ----------
Magn-Kef-type        ---------- ---------- ---------- ---------- ---------- ----MTGGMK PPARK----- ---------- ---------- ---------- ---------- ----------
Rals-Put-ATPsenIRK   ---------- ---------- ---------- ---------- ---------- ----MDIRSF LSRRT----- ---------- ---------- ---------- ---------- ----------
Buh-KirBAC1.1        ---------- ---------- ---------- ---------- ---------- ----MNVDPF SPHSSDSFAQ AASPARKPP- ---------- ---------- ---------- ----------
Buh-Kef-type         ---------- ---------- ---------- ---------- ---------- ----MATKPT DRPSAFGNGE ARAPTRRP-- ---------- ---------- ---------- ----------
Chrom-prob-ATPsenIRK ---------- ---------- ---------- ---------- ---------- ----MAPRSS ---------- ---------- ---------- ---------- ---------- ----------
Clustal Consensus
```

Alignment: 20IRK HR-CI-CE-CB-BAC(continue)

```
                     ....|....| ....|....| ....|....| ....|....| ....|....| ....|....| ....|....| ....|....| ...M1..|...| ....|....| ....|....| ....|....|
                       125        135        145        155        165        175        185        195        205        215        225        235
HR-TuIRKA            GSEYINDMHA SSRSLASRNM HRRKNCRFIK KSGHCNVGHT NVNKKPQRFL ADIFTTCVDL KWRWNLLLFS AAFILSWLFF GFIYWIISYI HGDFST---- ------NQTD IQCISNLESS
CI-IRK               -----YHMEE DDQVKERSNL ISHKGVRFIK KTGHCNVSHS NLTDKPRRFI ADIFTTGVDL KWRWNLFVFS AAFVVSWICF ALVFWLISYL HGDFAVR--- ----AANESF TPCVNNLDIN
HR-TuGIRKAa          RPSCVPAITR DTPGGVKRPT KRRRQTRFVT KNGHCNVRHG NVEDR-SRYL SDLFTTLVDL EWRYNVMIFI STYTITWLVF AFVWWFISFC RNDLNLD--- ------VKNQ TVCVIGIKS-
CI-GIRKA/G           ---------- ---------M KKRRQTRFVT KKGHCNVRHG NVKDR-LRYF ADLFTSIVDL KWRYNVAIFV ATYTFTWLVF AFLWWFVSFL RGDFDK---- ------MNSD EVCVIGIDS-
HR-TuGIRKB           -TTTLSNGDH EVVQ--IKSK TKQPG-RFMT KTGHCNIRRS ALQMG-TRYM TDIFTTLVDL RWKYNMIIFV FVYTAAWSMF GFLWWMVAFV RGDTD----- INVHNGTDSR KPCVQNVYS-
CI-GIRKB/G           WKSDVGNNHS SRRS--KGSE KKKPTTRFMQ KNGRCNIRQD AAEIR-RRYL RDIFTTLVDM SWRSNLLFFI ATYICAWSSI GLIWWIIALL RGDVADHA-L SKTSNVTSNH TACVQNVYS-
CI-IRK/AR            ---------- ---------- ---------- -MGRLNVFS- -------RFS SDIFTTLLDS SWSWILFITM TVYVGHWIIF GILYWVFAVA NNDYEKIFGP TVTVHEASSE TPCVFEVYD-
CB-Irk2/AR           KNKKYPFFKT PRLGGSRRIR NRLVQKQGLC NISLKNVPKQ RRK-----YF SDIFTTVIEM KWRWCLLYFS LSFMISWSFF ATVYYLIAKQ HGDIEQI--- -----ANATW TPCIVNVHN-
CE-Irk2/AR           KNKKYPFFKT PRLGGSRRIR NRLVQKQGLC NISLKNVPKQ RRK-----YF SDIFTTVIEM KWRWCLLYFS LSFMISWSFF ATVYFLIAKQ HGDIEQI--- -----ANATW TPCIVNVHN-
CB-Irk1/AR           RKS------T KKLK-----K SRLVGKNGIC NVYNTNVPKK DRQ-----YL RDIFTTLIDV KWRWMLLLFA SAFVLSWSVF GTTYYLIALF HGDLTDP--- -----LPANH TACVVNLDS-
CE-Irk1/AR           RKS------T KKLK-----K SRLVGKNGIC NVYNTNVPKK DRQ-----YL RDIFTTMIDV KWRWMLMLFA SAFVLSWSIF GTTYYLIALV HGDLSLP--- -----TPVNH TACVMNLDS-
CB-Irk3/AR           ---------- ---------R ARLVSKDGKT LLNNIQIPER FRQAYKWRYC RNWFHLIIEC NWRSICILFL LGFIVSWTIF ALIYYVISKL K--------- -----TVKKD ESCIANVDT-
CE-Irk3/AR           ---------- ---------R ARLVSKDGKT LLNNIQIPER FRQAYKWRYC RNWFHLIIEC NWRTICILFL LGFIVSWTIF AVIYYVISRL K--------- -----TVKKD ESCIANVDT-
Nos-K-channel-like   ---------- ---------- VQIKIQDGQF EIIGMGVWHS YWR------- -DPYHLLLTI PWTGFLLLIC LSYLAINLIF ALAYWLG--- ---------- ---------- GDCIANAKPG
Nos-Kef-type         ---------- ---------P IQIQVRDGKF EIMGMGVWHS YWR------- -DPYHLLLTI PWAGFLLLIC TFYITINALF ALAYLIG--- ---------- ---------- GDCIANARPG
Magn-Kef-type        ---------- ---------- PRILNSDGSS NITRLGLEKR GWLD------ -DHYHDLLTV SWPVFITLIT GLYLVTNALF ALAYLAC--- ---------- ---------- GDVIENARPG
Rals-Put-ATPsenIRK   ---------- ---------R RGRKLWSGTQ HVIAHGMPPL GWR------- -DIYHRALEV NWPTFFALLA GLFFMLNTVF AGLYSLG--- ---------- ---------- TASIANQSPP
Buh-KirBAC1.1        ---------- ---------R GGRRIWSGTR EVIAYGMPAS VWR------- -DLYYWALKV SWPVFFASLA ALFVVNNTLF ALLYQLG--- ---------- ---------- DAPIANQSPP
Buh-Kef-type         ---------- ---------R GSRELRLDDR VIIAHGVPTP LWQ------- -DLYHRALAV RWPTFFVSLA VLFLLLNTAF ATLYMLG--- ---------- ---------- SAPIANQFPA
Chrom-prob-ATPsenIRK ---------- ---------K PARRIEAGGR SLFIHSQPRH SLH------- -DLYHYCMTI SWPQFYLFIA MSFIALNLLF AGLYQLG--- ---------- ---------- PGGIANQFPQ
Clustal Consensus                                                         : :   :    *          :   I    : .  :                                :
```

Alignment: 20IRK HR-CI-CE-CB-BAC(continue)

```
                   ..|....|..H5..|....|..|....| |....|....|..M2.|....|....||....| ....|....| ....|....| ....|....| ....|....| ....|....| ....|....|
                      245        255        265        275        285        295        305        315        325        335        345        355
HR-TuIRKA          SPFTSTFLFS LETQTTIGYG SRAVTEECPM AILTVVIQSV YGCIIDAFMI GLIMAKISRP KKRAETLLFS KKAVISMRDG Q---LCLMVR VGNLRK-SHL VEATIRMQYI YSRETIEGEF
CI-IRK             HPFTSSFLFS LETQTTIGYG FRVVTEECPF TVFMVVVQSV FGCILDAFMI GLIMAKISRP KKRAETLMFS NKAVINMRDG Q---LCLMVR VGNLRK-SHL VEATIRMQYI HSRETLEGEF
HR-TuGIRKAa        --FTSAFLFS IETQVTIGYG TRAITEHCPE AIILLLIQSL LGSIVDAFMV GCMFVKISQP KKRAETIMFS HKAVMSLRDG Q---MCLMFR VGDLRN-SHI VEAQIRAKLI KSRQTQEGEF
CI-GIRKA/G         --FMSAFLFS IETQVTIGYG TRAITDVCPE AIILLIVQSI FGSIVDAFMV GCMFVKISQP NKRAETLMFS EKSVISLRDG K---MCLMFR VGDLRN-SHI VEAQIRAKLI KSRQTQEGEF
HR-TuGIRKB         --YATAFLFY IETETTIGYG KRAMTDQCPE AILLFVIQSL LGSIVDAFMV GCIFIKLSQP KNRAETLVFS EHCILTQRDG K---YCLMFR VANLRN-SLL IQCKIRAKIV KSRQTLEGEF
CI-GIRKB/G         --YSTAFLFF IETETSVGYG KRAITAHCPE AIILFVMQCL LGSILDAFMV GCIFIKISQP KNRAETLVFS EHCVISPRDR K---YCLMFR VGNLRN-SLI VQCRIRAKYV KSRQTQEGEF
CI-IRK/AR          --LLSAFLFS MESETTIGYG LRGMTTTCPI AVACLTIQCI VSAIFDTWVI GMCYARLASP SARSRTTLFS QNAVICKRDG K---RCLLVR VANLRK-SLL LNVSVRAKLI NLTANEKHSG
CB-Irk2/AR         --ELNAFLFS FTTQTTIGYG FRYPTDACPL TIVTMCFQFM WGVMTQTLMA GIIFSKLARP IKRAATLIFS KNAVICLRDG K---LCLLFR VGDMRK-SSL AEAHVRLQMI KRCVTYEGEL
CE-Irk2/AR         --ELNAFLFS FTTQTTIGYG FRYPTDACPL TIVTMCFQFM WGVMTQTLMA GIIFSKLARP IKRAATLIFS KNAVICLRDG K---LCLLFR VGDMRK-SSL AEAHVRLQMI KRCVTYEGEL
CB-Irk1/AR         --VYSSFLFA VETHHTIGYG HRYITTECYL AGVIVCLQAI CALLLQSFMV GIVFAKMARP KKRAETIIFS DKAVICLRDG Q---LCFLCR VGDMRN-THL VEAHVRLQFI TDRETNEGEI
CE-Irk1/AR         --VYSSFLFA VETHHTIGYG HRYITTECYL AGAIVCLQAI CALLLQSFMV GIVFAKMARP KKRAETIIFS DKAVICLRDG Q---LCFLCR VGDMRN-THL VEAHVRLQFI TDRETNEGEI
CB-Irk3/AR         --FISAFLFS MESQHTIGYG LRYMTDLCPP AYLTLCVQCV VGVFLQTILA GIVIAKILRP KKRRQEMRFS RMAVIGPLDE HDKRPTLMIR LADIQEKLFL AESHVRLYMA CSKINSRGDR
CE-Irk3/AR         --LISAFLFS MESQHTIGYG LRYMTDLCPP AYLTLCVQCV VGVFLQTILA GIVIAKILRP KKRRQEMRFS RMAVIGPLDE HDKRPTLMIR LADIQEKLFL AESHVRLYMA CSKINSRGDR
Nos-K-channel-like N-FLDLFFFS VQTLASIGYG --AMYPKTLY ANTVVTIEAM IGLVGIAVMT GLAFARFSRP SAR---VIFS RVAVITPYND V---PTLMFR TANQRR-NLI LEAQMRVYLM RDEITAEGGF
Nos-Kef-type       S-FLDVFFFS VQTLASIGYG --AMYPKTTY ANIIVTIEAM IGLVGIAVMT GLAFARFSRP TAR---VLFS RIAVITPHNA M---PTLIFR TANQRR-NMI LEAQMRVYLM RDEITLEGQF
Magn-Kef-type      S-FTDAFFFS VQTMATIGYG --KLIPIGPL ANTLVTLEAL CGMLGLAVAA SLIYARFTRP TAG---VLFS SRMVISDFEG K---PTLMMR LANLRI-EQI IEADVHLVLV RSEISQEGMV
Rals-Put-ATPsenIRK G-FVGAFFFS VETLATVGYG --DMHPQTLY AHCIATLEIF VGMSGIALAT GLVFARFSRP RAK---IMFV RNPIVRTLNG K---PTLMVR AANARQ-NVI AEATAKLRLL QDERSPEGYT
Buh-KirBAC1.1      G-FVGAFFFS VETLATVGYG --DMHPQTVY AHAIATLEIF VGMSGIALST GLVFARFARP RAK---IMFA RHAIVRPFNG R---MTLMVR AANARQ-NVI AEARAKMRLM RREHSSEGYS
Buh-Kef-type       G-FGGAFFFS VETLATVGYG --DMHPQTVY AHWIATLEIF VGMSSIALAT GLIFARFSRP HAK---IMFA RYAVIRPLDG R---MTLMVR SANARQ-NVI AEAHARLRLL RQETTAEGYT
Chrom-prob-ATPsenIRK G-FLGAFFFS VETLATVGYG --DMHPQTLY THWISMLEIF IGMMSIALIT GVTFARFSRP RAR---IVFT GNPVVRPLNG E---QVLMLR AANARN-NVI IDASARLRLL QHEVTAEGTA
Clustal Consensus       *:*  . :   ::***                :     .: .  .      :      .   :: *         *     ::    :          :: *  .: :     :   :    :             . .
```

Alignment: 20IRK HR-CI-CE-CB-BAC(continue)

```
                   ....|....| ....|....| ....|....| ....|....| ....|....| ....|....| ....|....| ....|....| ....|.|..| ....|....| ....|....| ....|....|
                      365        375        385        395        405        415        425        435        445        455        465        475
HR-TuIRKA          IPLEQVDLHL D-LKNDSDRL FLVTPQTICH PIDSDSPLYH LNKESLKEAN ---FEVIVIL EGMVEATGMT TQARASYVPD EIMWGHRFEN VITFSRTS-R YNVDFRKFDR SYEVPETPKC
CI-IRK             IPLEQIDLHL D-LKNDSDRL FLVTPQTICH PIDENSPLWE LNAEDLPHAN ---FEVILIL EGMVEATGMT TQARASYLPN EILWGHRFQN MISFTRNN-G YKVNFGKFNK TYMTPNSPHQ
HR-TuGIRKAa        MALDQTDFNV G-FTTGADRL FLVTPLIICH IIDEKSPFWE MSAEDLINEE ---FEIVVIL EGMVEATGMT CQARSSYVED EVLWGQRFMQ VLMLEKG--Y FEVNYNNFHD TFEVS-SPTA
CI-GIRKA/G         MALDQTDLDV G-YTTGADRL FLVTPLIICH VIDEKSPFWN MSQSDLKEEE ---FEIVVIL EGMVEATGMT CQARSSYVED EVMWGQRFMP VLMLEKG--F YDVNYSNFHD HFEVD-TPDI
HR-TuGIRKB         IGLHQDDINV G-FDTGADNL FLVTPLIICH EIDHRSPFYN TNAEDLQKDK ---FEIIVIL EGMIESTGMI CQARTSYLNT EVLWGHRFMP VLFHARD--H FSVDHSEFHT TYEVP-MPKQ
CI-GIRKB/G         IGLHQEDINV G-FDTGADNL FLVTPLIICH EIDEKSPFYE MSMEDLKKEE ---FEIIVIL EGMIESTGMI CQARTSYLST EVLWGHRFMP VLFHARG--H FRVDHSEFHS IYEVT-MPRI
CI-IRK/AR          YQLMLEQRDL A-FQNN-ATT LLTAPVEYCH IIEKNSPLFG IPSDMYHRSK QNRFEIVFIL TGTLESTGMT MHAQTSYLSS EIDYGHRFAP ILSKNYAKSQ YEVNFKRFHD TVPEPISLRN
CB-Irk2/AR         LPFHQFDMDV GYDQG-LDRV FVIWPITICH EIDERSPLWE IGAEDLKSAK ---FEIIAIL EGVVESVGST TQARTSYLPS EILWGHRFEK LVHYKKENGQ YNIDFGKFHN VYSINTPTCS
CE-Irk2/AR         LPFHQFDMDV GYDQG-LDRV FVIWPITICH EIDERSPLWE IGADDLKSAK ---FEIIAIL EGVVESVGST TQARTSYLPS EILWGHRFEK LVHYKKENGQ YNIDFGKFHN VYSVNTPTCS
CB-Irk1/AR         EPLHQFEMKV GPSISDDDRL FLVWPTTLCH VIDSRSPLYN YNQQSLMSAQ ---FEIIVLL EGIVESTGMT AQAKTSYLPS EISWGHRFRK LVTYQRSNGS YQIDYDLFNS TYPVRTPAMS
CE-Irk1/AR         EPLHQFEMKV GPSIADDDRL FLVWPTTLCH VIDSRSPLYN YNQQTLMSAQ ---FEIIVLL EGIVESTGMT AQAKTSYLPS EVLWGHRFRK LVTYQRSNGS YQIDYDLFHS TYPVRTPAMS
CB-Irk3/AR         ELIGVKDMNV GYDSG-WDRV LLLWPIIVRH VINEDSPLYG MSRDNIAKAD ---FELIMTV EGIVEATGMT FQARTSFLPD EILWGYKFKP MVLMNEKLSK YEVHYSLFDQ TERVCDFDAQ
CE-Irk3/AR         ELIGVKDMNV GYDSG-WDRV LLLWPIIVRH VINEDSPLYG MSRENIVKAD ---FELIMTV EGIVEATGMT FQARTSFLPD EILWGYKFKP MVLMNEKLSK YEVHYSFFDQ TERVCDFDAQ
Nos-K-channel-like IRRFHDLKLL R-----NQTP SFTLSWLALH PIDEFSPLYG MSAESLIQTN ---TNIIISV SGIDETVAQV VHARYTYTAS NILWNSRFVD IFHHTSDG-H RYIDYKYFHD VLPLDKIG--
Nos-Kef-type       MRRFYDLKLL R-----NQTP SFTLSWSVMH VIDEFSPLYG MTPESLIQTN ---TILIVSL SGIDETVAQV VHARHTYAAN EILWNNQFAD IFYHAPDG-H RYIDYNRFHD VSPLDEML--
Magn-Kef-type      FRRFHDLTLT R-----SRSP IFSLSWTVMH PIDHHSPIYG ETDETLRNSH ---SEFLVLF TGHHEAFAQN VHARHAYSCD EIIWGGHFVD VFTTLPDG-R RALDLGKFHE IAQ-------
Rals-Put-ATPsenIRK LYKIRDLTLV R-----SEHP IFLLGWILMH VIDETSPLFG ATAASLAAGD ---ALLLLTI GGSDETAAQT LLARHSWRHD EIRWGHRYVD LMH-DEGN-V THVDYANFHE TVPVDEPAAG
Buh-KirBAC1.1      LMKIHDLKLV R-----NEHP IFLLGWNMMH VIDESSPLFG ETPESLAEGR ---AMLLVMI EGSDETTAQV MQARHAWEHD DIRWHHRYVD LMS-DVDG-M THIDYTRFND TEPVEPPGAA
Buh-Kef-type       LRKLYDLTLV R-----DQHP VFKLGWTVMH IVDESSPLFG ETAKSSSKRT ---SVI---- ---------- ---------- ---------- ---------- ---------- ----------
Chrom-prob-ATPsenIRK FRKLYDLALV R-----DQHP MFVLGWTLVH KIDEGSPLYQ RDAASLAVAR ---AQLILSI NGIDETTIQP MVARHTFSHQ DIRWNHAFAD LIYTDEDE-N DHVDYQRLQE VSPLPPKEE-
Clustal Consensus                          . I     *  ::   **::                   .                              I
```

Alignment: 20IRK HR-CI-CE-CB-BAC(continue)

```
                        ....|....| ....|....| ....|....| ....|....| ....|....| ....|....| ....|....| ....|....| ....|....| ....|....| ....|....| ....|....|
                             485        495        505        515        525        535        545        555        565        575        585        595
HR-TuIRKA               SSKYLQDMQN ADTVSERIML QDKEQNR--- ---------- --KNTSKDAT REDFSVSDGR DSGYTAPEDY QATDSNKTLN STSTCGSSYI NHDTENRRRS WTKKAIEDTM NKNSNQYEST
CI-IRK                  SAKYLSCASN SE--MNTLVE SSMNNNK--- ---------- --KNRQADSE SSSISASDGK DSGYTAVEDL TRAYSSQTLN ---------- ---TEN---- --NKVVS--- ----------
HR-TuGIRKAa             SAKEQAEERI KQRLNEANNS PGALSMHRTL PRSPRVQESA SLSSSFPTPT VRRKKTSIAN NCYPDEVEEN NKSDSPGIEK TNVQSNENNN NSLQPVLASY PVHSTLGQQQ ASSQSEADLL
CI-GIRKA/G              SAKEQ--QLM KQLPQEGGKE V--------- ---------- ---------- ---------- ---------- ---------- ---------- ---------- ---------- ----------
HR-TuGIRKB              SMRRFHDAQV QNN------- ---------- ---------- ---------- ---------- -------TKQ WHPGSSG--- ---------- ---------- ---------- ----------
CI-GIRKB/G              SMLEYQKSDP QHVNGSAKNT SNKLNAQ--- ---------P PQSVFTVRKS IHQKSLCTSN SILTSCDEQQ QRASSSGNFK INSSTRRAVS D--------- ---------- ----------
CI-IRK/AR               YDVTNDSTLN GRSVVTSLIE ET-------- ---------- ---------- ---------- -------EED IISVSDEVFS AV-------- ---------- ---------- ----------
CB-Irk2/AR              AAEIERLRTE GTFNESDYQM YPPSDNK--- ---------- ---LNLLDND EMSPVRDSMD LSNQVPQIQI LRRDSDEDPD AGEES----- MELVDLG--- ---------- ----------
CE-Irk2/AR              AAEIERLRSE GIFNESEYQM YPPSDNK--- ---------- ---LNLLDND EMSPVRDSID LN-QVPQIQI LRRDSDEDPD AGEES----- MELVDLG--- ---------- ----------
CB-Irk1/AR              PAEFYSSKP- ---NLKDYYC HDSQEHK--- ---------- ---LEDNRSS DSTPLPSPSP YSYPSTPMNH FQSTSNSPVF SNAHSKFNTE AVTCEAG--- ---------- ----------
CE-Irk1/AR              PAEFYSSKP- ---NLKDYYC HDSHEHK--- ---------- ---LEDNRSS DSTPLPSPSP YSYPSTPLNH FQSSSNSPVF SNNHSKFNTE AVTCEAG--- ---------- ----------
CB-Irk3/AR              TIETEETEDD HHHNASGFL- ---------- ---------- ---------- ---------- ---------- ---------- ---------- ---------- ---------- ----------
CE-Irk3/AR              TIETEETEDE HHHNASGFL- ---------- ---------- ---------- ---------- ---------- ---------- ---------- ---------- ---------- ----------
Nos-K-channel-like      ---------- ---------- ---------- ---------- ---------- ---------- ---------- ---------- ---------- ---------- ---------- ----------
Nos-Kef-type            ---------- ---------- ---------- ---------- ---------- ---------- ---------- ---------- ---------- ---------- ---------- ----------
Magn-Kef-type           ---------- ---------- ---------- ---------- ---------- ---------- ---------- ---------- ---------- ---------- ---------- ----------
Rals-Put-ATPsenIRK      ASSDQA---- ---------- ---------- ---------- ---------- ---------- ---------- ---------- ---------- ---------- ---------- ----------
Buh-KirBAC1.1           PDAQAFAAKP GEGDARPV-- ---------- ---------- ---------- ---------- ---------- ---------- ---------- ---------- ---------- ----------
Buh-Kef-type            ---------- ---------- ---------- ---------- ---------- ---------- ---------- ---------- ---------- ---------- ---------- ----------
Chrom-prob-ATPsenIRK    ---------- ---------- ---------- ---------- ---------- ---------- ---------- ---------- ---------- ---------- ---------- ----------
Clustal Consensus
```

Alignment: 20IRK HR-CI-CE-CB-BAC(continue)

```
                        ....|....| ....|....| ....|....| ....|....| ....|....| ....|....| ....|....| ....|....| ....|....| ....|....| ....|....| ....|....|
                             605        615        625        635        645        655        665        675        685        695        705        715
HR-TuIRKA               NMDKCQTSSD ILPNMKQDMQ IKTNRNSADM TAQMTLTHNE KNEALNSTAR PRSGNVRIST AIPLDILKIS TVTVPEEVAK NKNGTSFIGD QNPKSNLKEL QNNNESKKST EILSGGRSSL
CI-IRK                  ---------- ---------- ---------- ---------- ---------- ---------- ---------- ---------- ---------- ---------- ---------- ----------
HR-TuGIRKAa             RLNKRLASVN ETAEIENVNE ADDRIYFRKS IASLLGGGYQ AMNRRNDSYF ADDSYDYHHR RNPLGRPKSA SVSVPSLWRS SEISDHPFAD YLKSATPRGD GKNFYGTQNA V---------
CI-GIRKA/G              ---------- ---------- ---------- ---------- ---------- ---------- ---------- ---------- ---------- ---------- ---------- ----------
HR-TuGIRKB              -------YVG NTAA------ ---------- ---------- ---------- ---------- ---------- ---------- ---------- ---------- ---------- ----------
CI-GIRKB/G              ------ANVS ATVAVENENV PESGSDVIPR RMNLLGSRQD LMLERRWS-- ---------- ----GGLEMK EPNINVLI-- ---------- ---------- ---------- ----------
CI-IRK/AR               ---------- ---------- ---------- ---------- ---------- ---------- ---------- ---------- ---------- ---------- ---------- ----------
CB-Irk2/AR              ------IHHK CSIRSQSPII NSTAHLLQPM HKNSCSVRRG MLAPPASHS- ---------- ---------- ---------- ---------- ---------- ---------- ----------
CE-Irk2/AR              ------IHHK CSIRSQSPII NSTSHLLQPM HKNSCSVRRG MLAPPASHS- ---------- ---------- ---------- ---------- ---------- ---------- ----------
CB-Irk1/AR              ------MLCP PTIVVQCPST CASPNHMRRT RNQMDKSRTS SSCDLTRPST ALSDLEEECS DSGSPTKCQS PPVAPIHIEI VSET------ ---------- ---------- ----------
CE-Irk1/AR              ------MLCP PTIVVQCPST CASPNHMRRT RNQMDKSR-- -----TRYSI A--------- ---------- ---------- ---------- ---------- ---------- ----------
CB-Irk3/AR              ---------- ---------- ---------- ---------- ---------- ---------- ---------- ---------- ---------- ---------- ---------- ----------
CE-Irk3/AR              ---------- ---------- ---------- ---------- ---------- ---------- ---------- ---------- ---------- ---------- ---------- ----------
Nos-K-channel-like      ---------- ---------- ---------- ---------- ---------- ---------- ---------- ---------- ---------- ---------- ---------- ----------
Nos-Kef-type            ---------- ---------- ---------- ---------- ---------- ---------- ---------- ---------- ---------- ---------- ---------- ----------
Magn-Kef-type           ---------- ---------- ---------- ---------- ---------- ---------- ---------- ---------- ---------- ---------- ---------- ----------
Rals-Put-ATPsenIRK      ---------- ---------- ---------- ---------- ---------- ---------- ---------- ---------- ---------- ---------- ---------- ----------
Buh-KirBAC1.1           ---------- ---------- ---------- ---------- ---------- ---------- ---------- ---------- ---------- ---------- ---------- ----------
Buh-Kef-type            ---------- ---------- ---------- ---------- ---------- ---------- ---------- ---------- ---------- ---------- ---------- ----------
Chrom-prob-ATPsenIRK    ---------- ---------- ---------- ---------- ---------- ---------- ---------- ---------- ---------- ---------- ---------- ----------
Clustal Consensus
```

Alignment: 20IRK HR-CI-CE-CB-BAC(continue)

```
                        ....|....| ....|....| ....|....| ....|....| ....|....| ....|....
                            725        735        745        755        765        775
HR-TuIRKA               LVPRQKKTQT EHVNILATTR QDDIVQQIKE DPLENKSLLD NTSPVKKICE VEMPEIKIE
CI-IRK                  ---------- ---------- ---------- ---------- ---------- ---------
HR-TuGIRKAa             ---------- ---------- ---------- ---------- ---------- ---------
CI-GIRKA/G              ---------- ---------- ---------- ---------- ---------- ---------
HR-TuGIRKB              ---------- ---------- ---------- ---------- ---------- ---------
CI-GIRKB/G              ---------- ---------- ---------- ---------- ---------- ---------
CI-IRK/AR               ---------- ---------- ---------- ---------- ---------- ---------
CB-Irk2/AR              ---------- ---------- ---------- ---------- ---------- ---------
CE-Irk2/AR              ---------- ---------- ---------- ---------- ---------- ---------
CB-Irk1/AR              ---------- ---------- ---------- ---------- ---------- ---------
CE-Irk1/AR              ---------- ---------- ---------- ---------- ---------- ---------
CB-Irk3/AR              ---------- ---------- ---------- ---------- ---------- ---------
CE-Irk3/AR              ---------- ---------- ---------- ---------- ---------- ---------
Nos-K-channel-like      ---------- ---------- ---------- ---------- ---------- ---------
Nos-Kef-type            ---------- ---------- ---------- ---------- ---------- ---------
Magn-Kef-type           ---------- ---------- ---------- ---------- ---------- ---------
Rals-Put-ATPsenIRK      ---------- ---------- ---------- ---------- ---------- ---------
Buh-KirBAC1.1           ---------- ---------- ---------- ---------- ---------- ---------
Buh-Kef-type            ---------- ---------- ---------- ---------- ---------- ---------
Chrom-prob-ATPsenIRK    ---------- ---------- ---------- ---------- ---------- ---------
Clustal Consensus
```
