# Supplementary material for: Ancient intron insertion sites and palindromic genomic duplication evolutionally shapes an elementally functioning membrane protein family
Source: BMC Evol Biol. 2007 Aug 20;7:143. doi: 10.1186/1471-2148-7-143 (PMC1999503; doi:10.1186/1471-2148-7-143)
Supplement: Additional file 7 — Consrved sequences in alignment of IRK AA sequences. The selected AA sequences from the alignment of 115 AA sequences to illustrate an exclusively conserved intron intervening site among the tunicate and vertebrate GIRK group. For the explanation of colored characters, see the legend of Fig. 9 in the original paper. [file 1471-2148-7-143-S7.pdf]

Alignment: GIRK

```
               ....|....| ....|....| ....|....| ....|....| ....|....| ....|....| ....|....| ....|....| ....|....| ....|....| ....|....| ....|....|
                   5         15         25         35         45         55         65         75         85         95        105        115
CI-GIRKB/G     ---------- ---------- ---------- ---------- ---------- ---------- ---------- ---------- ---------- ---------- ---------- -MEMEHLKPE
HR-TuGIRKB     ---------- ---------- ---------- ---------- ---------- ---------- ---------- ---------- ---------- ---------- ---------- ----------
CI-GIRKA/G     ---------- ---------- ---------- ---------- ---------- ---------- ---------- ---------- ---------- ---------- ---------- ----------
HR-TuGIRKAa    ---------- ---------- ---------- ---------- ---------- ---------- ---------- ---------- ---------- ---------- ---------- ----------
HS-Kcnj5/G     ---------- ---------- ---------- ---------- ---------- ---------- ---------- ---------- ---------- ---------- ---------- ----------
MM-Kcnj5/G     ---------- ---------- ---------- ---------- ---------- ---------- ---------- ---------- ---------- ---------- ---------- ----------
GG-Kcnj5/G     ---------- ---------- ---------- ---------- ---------- ---------- ---------- ---------- ---------- ---------- ---------- ----------
HS-Kcnj6/G     ---------- ---------- ---------- ---------- ---------- ---------- ---------- ---------- ---------- ---------- ---------- ----------
MM-Kcnj6/G     ---------- ---------- ---------- ---------- ---------- ---------- ---------- ---------- ---------- ---------- ---------- ----------
RN-Kcnj6/G     ---------- ---------- ---------- ---------- ---------- ---------- ---------- ---------- ---------- ---------- ---------- ----------
GG-Kcnj6/G     ---------- ---------- ---------- ---------- ---------- ---------- ---------- ---------- ---------- ---------- ---------- ----------
MM-Kcvnj9/G    ---------- ---------- ---------- ---------- ---------- ---------- ---------- ---------- ---------- ---------- ---------- ----------
RN-Kcnj9/G     ---------- ---------- ---------- ---------- ---------- ---------- ---------- ---------- ---------- ---------- ---------- ----------
HS-Kcnj9/G     ---------- ---------- ---------- ---------- ---------- ---------- ---------- ---------- ---------- ---------- ---------- ----------
MM-Kcnj3/G     ---------- ---------- ---------- ---------- ---------- ---------- ---------- ---------- ---------- ---------- ---------- ----------
RN-Kcnj3/G     ---------- ---------- ---------- ---------- ---------- ---------- ---------- ---------- ---------- ---------- ---------- ----------
HS-Kcnj3/G     ---------- ---------- ---------- ---------- ---------- ---------- ---------- ---------- ---------- ---------- ---------- ----------
GG-Kcnj3/G     ---------- ---------- ---------- ---------- ---------- ---------- ---------- ---------- ---------- ---------- ---------- ----------
```

Alignment: HS-IRK

```
               ....|....| ....|....| ....|....| ....|....| ....|....| ....|....| ....|....| ....|....| ....|....| ....|....| ....|....| ....|....|
                   5         15         25         35         45         55         65         75         85         95        105        115
CI-IRK         ---------- ---------- ---------- --MDNMG--- ---------- -LRRGRVENY GLP-----GL RHSSLRNG-- --CTLRNISE LPSVSGSVEI HNQS------ ------NMNN
HR-TuIRKA      ---------- ---------- --------MR YTIDTVSKVL HHTSTSKALV NARKSQIGEH CIVKEHRMDL SQKSPRLGRN IMDTVSNRIP IESRDAYIQI SNDSQDLDPT TDDTLRNMSN
HS-Kcnj2       ---------- ---------- ---------- ---------- ---------- ---------- ---------- ---------- ---------M GSVRTNRYSI VSS------- ----------
HS-Kcnj12      ---------- ---------- ---------- ---------- ---------- ---------- ---------- ---------- --------MT AASRANPYSI VSS------- ----------
HS-Kcnj4       ---------- ---------- ---------- ---------- ---------- ---------- ---------- ---------- ---------- ---------- ---------- ----------
HS-Kcnj14      ---------- ---------- ---------- ---------- ---------- ---------- ---------- ---------- --------MG LARALRRLSG ALDS------ ----------
HS-Kcnj16      ---------- ---------- ---------- ---------- ---------- ---------- ---------- ---------- ---------- ---------- ---M------ ----------
```

Alignment: HS-IRK/AR

```
               ....|....| ....|....| ....|....| ....|....| ....|....| ....|....| ....|....| ....|....| ....|....| ....|....| ....|....| ....|....|
                   5         15         25         35         45         55         65         75         85         95        105        115
CI-IRK/AR      ---------- ---------- ---------- ---------- ---------- ---------- ---------- ---------- ---------- ---------- ---------- ----------
HS-Kcnj13/AR   ---------- ---------- ---------- ---------- ---------- ---------- ---------- ---------- ---------- ---------- ---------- ----------
HS-Kcnj1a/AR   ---------- ---------- ---------- ---------- ---------- ---------- ---------- ---------- ---------- ---------- ---------- ----------
HS-Kcnj10/AR   ---------- ---------- ---------- ---------- ---------- ---------- ---------- ---------- ---------- ---------- ---------- ----------
MM-Kcnj10/AR   ---------- ---------- ---------- ---------- ---------- ---------- ---------- ---------- ---------- ---------- ---------- ----------
HS-Kcnj15/AR   ---------- ---------- ---------- ---------- ---------- ---------- ---------- ---------- ---------- ---------- ---------- ----------
```

First page

Alignment: GIRK(continue)

```
                 ....|....| ....|....| ....|....| ....|....| ....|....| ....|....| ....|....| ....|....| ....|....| ....|....| ....|....| ....|....|
                    125        135        145        155        165        175        185        195        205        215        225        235
CI-GIRKB/G       AEAKSLSVPG TPNTMTTRRT S--VVQMSAK LAALRGETVR EEVRSNSDLW KSDVGNNHSS RRSKGSEKKK PTTRFMQKNG RC----NIRQ DAAE------ IRRRYLRDIF TTLVDMSWRS
HR-TuGIRKB       ---------- ----MSLRRH SGGGVQMSAK LAALRGESIE GASMLDT--- TTTLSNGDHE VVQIKSKTKQ PG-RFMTKTG HC----NIRR SALQ------ MGTRYMTDIF TTLVDLRWKY
CI-GIRKA/G       ---------- ---------- ---------- ---------- ---------- ---------- ------MKKR RQTRFVTKKG HC----NVRH GNVK------ DRLRYFADLF TSIVDLKWRY
HR-TuGIRKAa      --MGVISNVS PDVTNKWRTD SQVLPALGNS KARDTLFQEK MVRTHSRRPS CVPAITRDTP GGVKRPTKRR RQTRFVTKNG HC----NVRH GNVE------ DRSRYLSDLF TTLVDLEWRY
HS-Kcnj5/G       ---------- --------MA GDSRNAMNQD MEIGVTPWDP KKIPKQARDY VPIATDRTRL LAE----GKK PRQRYMEKSG KC----NVHH GNVQ------ ETYRYLSDLF TTLVDLKWRF
MM-Kcnj5/G       ---------- --------MA GDSRNAMNQD MEIGVTSQDH KKIPKQARDY IPIATDRTRL LTE----GKK PRQRYMEKTG KC----NVHH GNVQ------ ETYRYLSDLF TTLVDLKWRF
GG-Kcnj5/G       ---------- --------MA RDSRIFMNQD MDIGVASREP KKIPKQARDD VPIATDRTRL ITAE---GKK PRQRYMEKSG KC----NVHH GNVQ------ ETYRYLSDLF TTLVDLKWRF
HS-Kcnj6/G       ---------- MAKLTESMTN VLEGDSMDQD VESPVAIHQP K-LPKQARDD LPRHISRDR- -------TKR KIQRYVRKDG KC----NVHH GNVR------ ETYRYLTDIF TTLVDLKWRF
MM-Kcnj6/G       --------MT MAKLTESMTN VLEGDSMDQD VESPVAIHQP K-LPKQARDD LPRHISRDR- -------TKR KIQRYVRKDG KC----NVHH GNVR------ ETYRYLTDIF TTLVDLKWRF
RN-Kcnj6/G       ---------- ---------- ------MDQD VESPVAIHQP K-LPKQARDD LPRHISRDR- -------TKR KIQRYVRKDG KC----NVHH GNVR------ ETYRYLTDIF TTLVDLKWRF
GG-Kcnj6/G       ---------- ---------N VLEEDSMEQD IESPVTIHQP K-LPKQARED LPKNLNKEC- -------AKR KIQRYVRKDG KC----NVHH GNVR------ ETYRYLTDIF ITLVDLKWRF
MM-Kcnj9/G       ---------- ---------- -----MAQEN AAFSPGSEEP P--------- ---------- -------RRR GRQRYVEKDG RC----NVQQ GNVR------ ETYRYLTDLF TTLVDLQWRL
RN-Kcnj9/G       ---------- ---------- -----MAQEN AAFSPGSEEP P--------- ---------- -------RRR GRQRYVEKDG RC----NVQQ GNVR------ ETYRYLTDLF TTLVDLQWRL
HS-Kcnj9/G       ---------- ---------- -----MAQEN AAFSPGQEEP P--------- ---------- -------RRR GRQRYVEKDG RC----NVQQ GNVR------ ETYRYLTDLF TTLVDLQWRL
MM-Kcnj3/G       ---------- ---------M SALRRKFGDD YQVVTTSSSG SGLQPQGPGQ GPQ------- ---QQLVPKK KRQRFVDKNG RC----NVQH GNLGS----- ETSRYLSDLF TTLVDLKWRW
RN-Kcnj3/G       ---------- ---------M SALRRKFGDD YQVVTTSSSG SGLQPQGPGQ GPQ------- ---QQLVPKK KRQRFVDKNG RC----NVQH GNLGS----- ETSRYLSDLF TTLVDLKWRW
HS-Kcnj3/G       ---------- ---------M SALRRKFGDD YQVVTTSSSG SGLQPQGPGQ DPQ------- ---QQLVPKK KRQRFVDKNG RC----NVQH GNLGS----- ETSRYLSDLF TTLVDLKWRW
GG-Kcnj3/G       ---------- ---------M SALRRKLGDE YQVVSTSASG GGLPP----- ---------- ---PRAAPRG KRQRFVDKNG RC----NVQH GNLGG----- ETSRYLSDLF TTLVDLKWRW
```

Alignment: HS-IRK(continue)

```
                 ....|....| ....|....| ....|....| ....|....| ....|....| ....|....| ....|....| ....|....| ....|....| ....|....| ....|....| ....|....|
                    125        135        145        155        165        175        185        195        205        215        225        235
CI-IRK           LR-------- -----KVSNS SSLQGMMT-- ---YHMEEDD QVKERSN--- ---------- ------LISH KGVRFIKKTG HC----NVSH SNLTD----- KPRRFIADIF TTGVDLKWRW
HR-TuIRKA        RSSNPIIMTC SGLNDNSSNS SSLQRIVYGS EYINDMHASS RSLASRN--- ---------- ------MHRR KNCRFIKKSG HC----NVGH TNVNK----- KPQRFLADIF TTCVDLKWRW
HS-Kcnj2         ---------- -------EED GMKLATMAVA NGFGNG--KS KVHTR----- ---------- --------QQ CRSRFVKKDG HC----NVQF INVGE----- KGQRYLADIF TTCVDIRWRW
HS-Kcnj12        ---------- -------EED GLHLVTMSG- ---ANGFGNG KVHTR----- ---------- --------RR CRNRFVKKNG QC----NIEF ANMDE----- KSQRYLADMF TTCVDIRWRY
HS-Kcnj14        ---------- -------GDS RAGDEEEAGP GLCRNGWAPA PVQSP----- ---------- -------VGR RRGRFVKKDG HC----NVRF VNLGG----- QGARYLSDLF TTCVDVRWRW
HS-Kcnj16        ---------- -------SYY GSSYHIINAD AKYPGYPPEH IIAEK----- ---------- --------RR ARRRLLHKDG SC----NVYF KHIFG----- EWGSYVVDIF TTLVDTKWRH
```

Alignment: HS-IRK/AR(continue)

```
                 ....|....| ....|....| ....|....| ....|....| ....|....| ....|....| ....|....| ....|....| ....|....| ....|....| ....|....| ....|....|
                    125        135        145        155        165        175        185        195        205        215        225        235
CI-IRK/AR        ---------- ---------- ---------- ---------- ---------- ---------- ---------- --------MG RL----NVFS ---------- ---RFSSDIF TTLLDSSWSW
HS-Kcnj13/AR     ---------- ---------- ---------- MDSSNCKVIA PLLS------ ---------- ---------Q RYRRMVTKDG HS----TLQM DGAQR----- -GLAYLRDAW GILMDMRWRW
HS-Kcnj1a/AR     ---------- MNASSRNVFD TLIRVLTESM FKHLRKWVVT RFFGHS---- ---------- ---------R QRARLVSKDG RC----NIEF GNVEA----Q SRFIFFVDIW TTVLDLKWRY
HS-Kcnj10/AR     ---------- --MTSVA--- -------KVY YSQTTQTESR PLMGPG---- ---------- ---------I RRRRVLTKDG RS----NVRM EHIA-----D KRFLYLKDLW TTFIDMQWRY
HS-Kcnj15/AR     ---------- ---------- -----MDAIH IGMSSTPLVK HTAGAG---- ---------- -------LKA NRPRVMSKSG HS----NVRI DKVD-----G IYLLYLQDLW TTVIDMKWRY
```

Second page

Alignment: GIRK(continue)

```
              ....|....|....|M1..|....|....|....|....|....|....|....|....|....|....|....|....|..H5|....|....|....|....|....|....|..M2|
                 245       255       265       275       285       295       305       315       325       335       345       355
CI-GIRKB/G    NLLFFIATYI CAWSSIGLIW WIIALLRGDV ADHAL----- ---------S KT-SNVTSNH TACVQNVYS- --YSTAFLFF IETETSVGYG KRAITAHCPE AIILFVMQCL LGSILDAFMV
HR-TuGIRKB    NMIIFVFVYT AAWSMFGFLW WMVAFVRGDT D--------- ---------I NV-HNGTDSR KPCVQNVYS- --YATAFLFY IETETTIGYG KRAMTDQCPE AILLFVIQSL LGSIVDAFMV
CI-GIRKA/G    NVAIFVATYT FTWLVFAFLW WFVSFLRGDF DK-------- ---------- -------MNSD EVCVIGIDS- --FMSAFLFS IETQVTIGYG TRAITDVCPE AIILLIVQSI FGSIVDAFMV
HR-TuGIRKAa   NVMIFISTYT ITWLVFAFVW WFISFCRNDL NLD------- ---------- ------VKNQ TVCVIGIKS- --FTSAFLFS IETQVTIGYG TRAITEHCPE AIILLLIQSL LGSIVDAFMV
HS-Kcnj5/G    NLLVFTMVYT VTWLFFGFIW WLIAYIRGDL DHV------- ---------- -----GDQEW IPCVENLSG- --FVSAFLFS IETETTIGYG FRVITEKCPE GIILLLVQAI LGSIVNAFMV
MM-Kcnj5/G    NLLVFTMVYT ITWLFFGFIW WLIAYVRGDL DHV------- ---------- -----GDQEW IPCVENLSG- --FVSAFLFS IETETTIGYG FRVITEKCPE GIILLLVQAI LGSIVNAFMV
GG-Kcnj5/G    NLLVFTMVYT ITWLFFGFIW WLIAYIRGDL DHL------- ---------- -----EDENW IPCVENLSG- --FVSAFLFS IETETTIGYG YRVITEKCPE GIVLLLIQAI LGSIVNAFMV
HS-Kcnj6/G    NLLIFVMVYT VTWLFFGMIW WLIAYIRGDM DHI------- ---------- -----EDPSW TPCVTNLNG- --FVSAFLFS IETETTIGYG YRVITDKCPE GIILLLIQSV LGSIVNAFMV
MM-Kcnj6/G    NLLIFVMVYT VTWLFFGMIW WLIAYIRGDM DHI------- ---------- -----EDPSW TPCVTNLNG- --FVSAFLFS IETETTIGYG YRVITDKCPE GIILLLIQSV LGSIVNAFMV
RN-Kcnj6/G    NLLIFVMVYT VTWLFFGMIW WLIAYIRGDM DHI------- ---------- -----EDPSW TPCVTNLNG- --FVSAFLFS IETETTIGYG YRVITDKCPE GIILLLIQSV LGSIVNAFMV
GG-Kcnj6/G    NLLIFVMVYT VTWLFFGMIW WLIAYMRGDM DHI------- ---------- -----GDSTW TPCVSNLNG- --FVSAFLFS IETETTIGYG YRVITDKCPE GIVLLLIQSV LGSIVNAFMV
MM-Kcnj9/G    SLLFFVLAYA LTWLFFGAIW WLIAYGRGDL EHL------- ---------- -----EDTAW TPCVNNLNG- --FVAAFLFS IETETTIGYG HRVITDQCPE GIVLLLLQAI LGSMVNAFMV
RN-Kcnj9/G    SLLFFVLAYA LTWLFFGAIW WLIAYGRGDL EHL------- ---------- -----EDTAW TPCVNNLNG- --FVAAFLFS IETETTIGYG HRVITDQCPE GIVLLLLQAI LGSMVNAFMV
HS-Kcnj9/G    SLLFFVLAYA LTWLFFGAIW WLIAYGRGDL EHL------- ---------- -----EDTAW TPCVNNLNG- --FVAAFLFS IETETTIGYG HRVITDQCPE GIVLLLLQAI LGSMVNAFMV
MM-Kcnj3/G    NLFIFILTYT VAWLFMASMW WVIAYTRGDL NKA------- ---------- -----HVGNY TPCVANVYN- --FPSAFLFF IETEATIGYG YRYITDKCPE GIILFLFQSI LGSIVDAFLI
RN-Kcnj3/G    NLFIFILTYT VAWLFMASMW WVIAYTRGDL NKA------- ---------- -----HVGNY TPCVANVYN- --FPSAFLFF IETEATIGYG YRYITDKCPE GIILFLFQSI LGSIVDAFLI
HS-Kcnj3/G    NLFIFILTYT VAWLFMASMW WVIAYTRGDL NKA------- ---------- -----HVGNY TPCVANVYN- --FPSAFLFF IETEATIGYG YRYITDKCPE GIILFLFQSI LGSIVDAFLI
GG-Kcnj3/G    NLFIFVLTYT VAWLFMASMW WVIAYMRGDL NKA------- ---------- -----HDDSY TPCVANVYN- --FPSAFLFF IETEATIGYG YRYITDKCPE GIILFLFQSI LGSIVDAFLI
```

Alignment: HS-IRK(continue)

```
              ....|....|....|....|....|....|....|....|....|....|....|....|....|....|....|....|....|....|....|....|....|....|....|....|
                 245       255       265       275       285       295       305       315       325       335       345       355
CI-IRK        NLFVFSAAFV VSWICFALVF WLISYLHGDF AVRA------ ---------- -----ANESF TPCVNNLDIN HPFTSSFLFS LETQTTIGYG FRVVTEECPF TVFMVVVQSV FGCILDAFMI
HR-TuIRKA     NLLLFSAAFI LSWLFFGFIY WIISYIHGDF ST-------- ---------- ------NQTD IQCISNLESS SPFTSTFLFS LETQTTIGYG SRAVTEECPM AILTVVIQSV YGCIIDAFMI
HS-Kcnj2      MLVIFCLAFV LSWLFFGCVF WLIALLHGDL DAS------- ---------- -------KEG KACVSEVNS- --FTAAFLFS IETQTTIGYG FRCVTDECPI AVFMVVFQSI VGCIIDAFII
HS-Kcnj12     MLLIFSLAFL ASWLLFGIIF WVIAVAHGDL E-------PA EG-------- -------RGR TPCVMQVHG- --FMAAFLFS IETQTTIGYG LRCVTEECPV AVFMVVAQSI VGCIIDSFMI
HS-Kcnj4      MLMIFSAAFL VSWLFFGLLF WCIAFFHGDL EASP--GVPA AGGPAAGGGG ----AAPVAP KPCIMHVNG- --FLGAFLFS VETQTTIGYG FRCVTEECPL AVIAVVVQSI VGCVIDSFMI
HS-Kcnj14     MCLLFSCSFL ASWLLFGLAF WLIASLHGDL AA------PP PP-------- ---------- APCFSHVAS- --FLAAFLFA LETQTSIGYG VRSVTEECPA AVAAVVLQCI AGCVLDAFVV
HS-Kcnj16     MFVIFSLSYI LSWLIFGSVF WLIAFHHGDL LND------- ---------- -------PDI TPCVDNVHS- --FTGAFLFS LETQTTIGYG YRCVTEECSV AVLMVILQSI LSCIINTFII
```

Alignment: HS-IRK/AR(continue)

```
              ....|....|....|....|....|....|....|....|....|....|....|....|....|....|....|....|....|....|....|....|....|....|....|....|
                 245       255       265       275       285       295       305       315       325       335       345       355
CI-IRK/AR     ILFITMTVYV GHWIIFGILY WVFAVANNDY EKIFG----- ---------P TVTVHEASSE TPCVFEVYD- --LLSAFLFS MESETTIGYG LRGMTTTCPI AVACLTIQCI VSAIFDTWVI
HS-Kcnj13/AR  MMLVFSASFV VHWLVFAVLW YVLAEMNGDL EL-------- ---------- -DHDAPPENH TICVKYITS- --FTAAFSFS LETQLTIGYG TMFPSGDCPS AIALLAIQML LGLMLEAFIT
HS-Kcnj1a/AR  KMTIFITAFL GSWFFFGLLW YAVAYIHKDL PE-------- ---------- ---FHPSANH TPCVENING- --LTSAFLFS LETQVTIGYG FRCVTEQCAT AIFLLIFQSI LGVIINSFMC
HS-Kcnj10/AR  KLLLFSATFA GTWFLFGVVW YLVAVAHGDL LE-------- ---------- ---LDPPANH TPCVVQVHT- --LTGAFLFS LESQTTIGYG FRYISEECPL AIVLLIAQLV LTTILEIFIT
HS-Kcnj15/AR  KLTLFAATFV MTWFLFGVIY YAIAFIHGDL EP-------- ---------- ---GEPISNH TPCIMKVDS- --LTGAFLFS LESQTTIGYG VRSITEECPH AIFLLVAQLV ITTLIEIFIT
```

Third page

Alignment: GIRK(continue)

```
              ....|....| ....|....| ....|....| ....|....| ....|....| ....|....| ....|....| ....|....| ....|....| ....|....| ....|....| ....|....|
                 365        375        385        395        405        415        425        435        445        455        465        475
CI-GIRKB/G    GCIFIKISQP KNRAETLVFS EHCVISPRDR KYCLMFRVGN LRNSLIVQCR IRAKYVKSRQ TQEGEFIGLH QEDINVGFDT GAD---NLFL VTPLIICHEI DEKSPFYEMS MEDLKK----
HR-TuGIRKB    GCIFIKLSQP KNRAETLVFS EHCILTQRDG KYCLMFRVAN LRNSLLIQCK IRAKIVKSRQ TLEGEFIGLH QDDINVGFDT GAD---NLFL VTPLIICHEI DHRSPFYNTN AEDLQK----
CI-GIRKA/G    GCMFVKISQP NKRAETLMFS EKSVISLRDG KMCLMFRVGD LRNSHIVEAQ IRAKLIKSRQ TQEGEFMALD QTDLDVGYTT GAD---RLFL VTPLIICHVI DEKSPFWNMS QSDLKE----
HR-TuGIRKAa   GCMFVKISQP KKRAETIMFS HKAVMSLRDG QMCLMFRVGD LRNSHIVEAQ IRAKLIKSRQ TQEGEFMALD QTDFNVGFTT GAD---RLFL VTPLIICHII DEKSPFWEMS AEDLIN----
HS-Kcnj5/G    GCMFVKISQP KKRAETLMFS NNAVISMRDE KLCLMFRVGD LRNSHIVEAS IRAKLIKSRQ TKEGEFIPLN QTDINVGFDT GDD---RLFL VSPLIISHEI NQKSPFWEMS QAQLHQ----
MM-Kcnj5/G    GCMFVKISQP KKRAETLMFS NNAVISMRDE KLCLMFRVGD LRNSHIVEAS IRAKLIKSRQ TKEGEFIPLN QTDINVGFDT GDD---RLFL VSPLIISHEI NEKSPFWEMS RAQLEQ----
GG-Kcnj5/G    GCMFVKISQP KKRAETLMFS NNAVISMRDE KLCLMFRVGD LRNSHIVEAS IRAKLIKSKQ TKEGEFIPLN QTDINVGFDT GDD---RLFL VSPLIISHEI NEKSPFWEMS RTQLEK----
HS-Kcnj6/G    GCMFVKISQP KKRAETLVFS THAVISMRDG KLCLMFRVGD LRNSHIVEAS IRAKLIKSKQ TSEGEFIPLN QTDINVGYYT GDD---RLFL VSPLIISHEI NQQSPFWEIS KAQLPK----
MM-Kcnj6/G    GCMFVKISQP KKRAETLVFS THAVISMRDG KLCLMFRVGD LRNSHIVEAS IRAKLIKSKQ TSEGEFIPLN QTDINVGYYT GDD---RLFL VSPLIISHEI NQQSPFWEIS KAQLPK----
RN-Kcnj6/G    GCMFVKISQP KKRAETLVFS THAVISMRDG KLCLMFRVGD LRNSHIVEAS IRAKLIKSKQ TSEGEFIPLN QTDINVGYYT GDD---RLFL VSPLIISHEI NQQSPFWEIS KAQLPK----
GG-Kcnj6/G    GCMFVKISQP KKRAETLVFS TNAVISMRDG KLCLMFRVGD LRNSHIVEAS IRAKLIKSKQ TKEGEFIPLN QTDINVGYYT GDD---RLFL VSPLIISHEI NQQSPFWEIS KAQLPK----
MM-Kcnj9/G    GCMFVKISQP NKRAATLVFS SHAVVSLRDG RLCLMFRVGD LRSSHIVEAS IRAKLIRSRQ TLEGEFIPLH QTDLSVGFDT GDD---RLFL VSPLVISHEI DAASPFWEAS RRALER----
RN-Kcnj9/G    GCMFVKISQP NKRAATLVFS SHAVVSLRDG RLCLMFRVGD LRSSHIVEAS IRAKLIRSRQ TLEGEFIPLH QTDLSVGFDT GDD---RLFL VSPLVISHEI DAASPFWEAS RRALER----
HS-Kcnj9/G    GCMFVKISQP NKRAATLVFS SHAVVSLRDG RLCLMFRVGD LRSSHIVEAS IRAKLIRSRQ TLEGEFIPLH QTDLSVGFDT GDD---RLFL VSPLVISHEI DAASPFWEAS RRALER----
MM-Kcnj3/G    GCMFIKMSQP KKRAETLMFS EHAVISMRDG KLTLMFRVGN LRNSHMVSAQ IRCKLLKSRQ TPEGEFLPLD QLELDVGFST GAD---QLFL VSPLTICHVI DAKSPFYDLS QRSMQT----
RN-Kcnj3/G    GCMFIKMSQP KKRAETLMFS EHAVISMRDG KLTLMFRVGN LRNSHMVSAQ IRCKLLKSRQ TPEGEFLPLD QLELDVGFST GAD---QLFL VSPLTICHVI DAKSPFYDLS QRSMQT----
HS-Kcnj3/G    GCMFIKMSQP KKRAETLMFS EHAVISMRDG KLTLMFRVGN LRNSHMVSAQ IRCKLLKSRQ TPEGEFLPLD QLELDVGFST GAD---QLFL VSPLTICHVI DAKSPFYDLS QRSMQT----
GG-Kcnj3/G    GCMFIKMSQP KKRAETLMFS EHAAISMRDG KLTLMFRVGN LRNSHMVSAQ IRCKLLKSRQ TPEGEFLPLD QLELDVGFST GAD---QLFL VSPLTICHVI DAKSPFYDLS QRSMQT----
```

Alignment: HS-IRK(continue)

```
              ....|....| ....|....| ....|....| ....|....| ....|....| ....|....| ....|....| ....|....| ....|....| ....|....| ....|....| ....|....|
                 365        375        385        395        405        415        425        435        445        455        465        475
CI-IRK        GLIMAKISRP KKRAETLMFS NKAVINMRDG QLCLMVRVGN LRKSHLVEAT IRMQYIHSRE TLEGEFIPLE QIDLHLDLKN DSD---RLFL VTPQTICHPI DENSPLWELN AEDLPH----
HR-TuIRKA     GLIMAKISRP KKRAETLLFS KKAVISMRDG QLCLMVRVGN LRKSHLVEAT IRMQYIYSRE TIEGEFIPLE QVDLHLDLKN DSD---RLFL VTPQTICHPI DSDSPLYHLN KESLKE----
HS-Kcnj2      GAVMAKMAKP KKRNETLVFS HNAVIAMRDG KLCLMWRVGN LRKSHLVEAH VRAQLLKSRI TSEGEYIPLD QIDINVGFDS GID---RIFL VSPITIVHEI DEDSPLYDLS KQDIDN----
HS-Kcnj12     GAIMAKMARP KKRAQTLLFS HNAVVALRDG KLCLMWRVGN LRKSHIVEAH VRAQLIKPRV TEEGEYIPLD QIDIDVGFDK GLD---RIFL VSPITILHEI DEASPLFGIS RQDLET----
HS-Kcnj4      GTIMAKMARP KKRAQTLLFS HHAVISVRDG KLCLMWRVGN LRKSHIVEAH VRAQLIKPYM TQEGEYLPLD QRDLNVGYDI GLD---RIFL VSPIIIVHEI DEDSPLYGMG KEELES----
HS-Kcnj14     GAVMAKMAKP KKRNETLVFS ENAVVALRDH RLCLMWRVGN LRRSHLVEAH VRAQLLQPRV TPEGEYIPLD HQDVDVGFDG GTD---RIFL VSPITIVHEI DSASPLYELG RAELAR----
HS-Kcnj16     GAALAKMATA RKRAQTIRFS YFALIGMRDG KLCLMWRIGD FRPNHVVEGT VRAQLLRYTE DSEG-RMTMA FKDLKLVN-- --D---QIIL VTPVTIVHEI DHESPLYALD RKAVAK----
```

Alignment: HS-IRK/AR(continue)

```
              ....|....| ....|....| ....|....| ....|....| ....|....| ....|....| ....|....| ....|....| ....|....| ....|....| ....|....| ....|....|
                 365        375        385        395        405        415        425        435        445        455        465        475
CI-IRK/AR     GMCYARLASP SARSRTTLFS QNAVICKRDG KRCLLVRVAN LRKSLLLNVS VRAKLINLTA NEKHSGYQLM LEQRDLAFQN N-A---TTLL TAPVEYCHII EKNSPLFGIP SDMYHRSKQ-
HS-Kcnj13/AR  GAFVAKIARP KNRAFSIRFT DTAVVAHMDG KPNLIFQVAN TRPSPLTSVR VSAVLYQ--- --ERENGKLY QTSVDFHLDG ISS-DECPFF IFPLTYYHSI TPSSPLATLL QHENPS----
HS-Kcnj1a/AR  GAILAKISRP KKRAKTITFS KNAVISKRGG KLCLLIRVAN LRKSLLIGSH IYGKLLKTTV TPEGETIILD QININFVVDA GNE---NLFF ISPLTIYHVI DHNSPFFHMA AETLL-----
HS-Kcnj10/AR  GTFLAKIARP KKRAETIRFS QHAVVASHNG KPCLMIRVAN MRKSLLIGCQ VTGKLLQTHQ TKEGENIRLN QVNVTFQVDT ASD---SPFL ILPLTFYHVV DETSPLKDLP LRSGE-----
HS-Kcnj15/AR  GTFLAKIARP KKRAETIKFS HCAVITKQNG KLCLVIQVAN MRKSLLIQCQ LSGKLLQTHV TKEGERILLN QATVKFHVDS SSE---SPFL ILPMTFYHVL DETSPLRDLT PQNLK-----
```

Forth page

Alignment: GIRK(continue)

```
                ....|....| ....|....| ....|....| ....|....| ....|....| ....|....| ....|....| ....|....| ....|....| ....|....| ....|....| ....|....|
                      485        495        505        515        525        535        545        555        565        575        585        595
CI-GIRKB/G      -EEFEIIVIL EGMIESTGMI CQARTSYLST EVLWGHRFMP VL---FHARG HFRVDHSEFH SIYEVT-MPR ISMLEYQKSD PQHVNGS--- ---------- ---------- ----------
HR-TuGIRKB      -DKFEIIVIL EGMIESTGMI CQARTSYLNT EVLWGHRFMP VL---FHARD HFSVDHSEFH TTYEVP-MPK QSMRRFHDAQ VQNN------ ---------- ---------- ----------
CI-GIRKA/G      -EEFEIVVIL EGMVEATGMT CQARSSYVED EVMWGQRFMP VL---MLEKG FYDVNYSNFH DHFEVD-TPD ISAKEQ--QL MKQLPQE--- ---------- ---------- ----------
HR-TuGIRKAa     -EEFEIVVIL EGMVEATGMT CQARSSYVED EVLWGQRFMQ VL---MLEKG YFEVNYNNFH DTFEVS-SPT ASAKEQAEER IKQRLNE--- ---------- ---------- ----------
HS-Kcnj5/G      -EEFEVVVIL EGMVEATGMT CQARSSYMDT EVLWGHRFTP VL---TLEKG FYEVDYNTFH DTYETN-TPS CCAKELAEMK REGRLLQ--- ---------- ---------- ----------
MM-Kcnj5/G      -EEFEVVVIL EGMVEATGMT CQARSSYMDT EVLWGHRFTP VL---TLEKG FYEVDYNTFH DTYETN-TPS CCAKELAEMK RSGRLLQ--- ---------- ---------- ----------
GG-Kcnj5/G      -EEFEIVVIL EGMVEATGMT CQARSSYMDT EVLWGHRFTP VL---TLEKD FYEVDYNSFH STYETN-TPV CCAKELAESR REGHLLS--- ---------- ---------- ----------
HS-Kcnj6/G      -EELEIVVIL EGMVEATGMT CQARSSYITS EILWGYRFTP VL---TLEDG FYEVDYNSFH ETYETS-TPS LSAKELAELA SRAELPL--- ---------- ---------- ----------
MM-Kcnj6/G      -EELEIVVIL EGMVEATGMT CQARSSYITS EILWGYRFTP VL---TLEDG FYEVDYNSFH ETYETS-TPS LSAKELAELA NRAELPL--- ---------- ---------- ----------
RN-Kcnj6/G      -EELEIVVIL EGMVEATGMT CQARSSYITS EILWGYRFTP VL---TLEDG FYEVDYNSFH ETYETS-TPS LSAKELAELA NRAELPL--- ---------- ---------- ----------
GG-Kcnj6/G      -EELEIVVIL EGMVEATGMT CQARSSYVTS EILWGYRFTP VL---TLEDG FYEVDYNSFH ETYETN-TPV YSAKELAEMA SRAELPL--- ---------- ---------- ----------
MM-Kcnj9/G      -DDFEIVVIL EGMVEATGMT CQARSSYLVD EVLWGHRFTS VL---TLEDG FYEVDYASFH ETFEVP-TPS CSARELAEAA ARLDAHL--- ---------- ---------- ----------
RN-Kcnj9/G      -DDFEIVVIL EGMVEATGMT CQARSSYLVD EVLWGHRFTS VL---TLEDG FYEVDYASFH ETFEVP-TPS CSARELAEAA ARLDAHL--- ---------- ---------- ----------
HS-Kcnj9/G      -DDFEIVVIL EGMVEATGMT CQARSSYLVD EVLWGHRFTS VL---TLEDG FYEVDYASFH ETFEVP-TPS CSARELAEAA ARLDAHL--- ---------- ---------- ----------
FR-Kcnj9/G      -EDFEIVVIL EGMVEATGMT CQARSSYLAE EVLWGHRFSP MM---SLAEG FFDVDYGAFH HTFEVD-TPS CSARELSLAA ARLDAHL--- ---------- ---------- ----------
MM-Kcnj3/G      -EQFEVVVIL EGIVETTGMT CQARTSYTED EVLWGHRFFP VI---SLEEG FFKVDYSQFH ATFEVP-TPP YSVKEQEEML LMSSP----- ---LIAP--- --AITNSKER HNSVECLDGL
RN-Kcnj3/G      -EQFEVVVIL EGIVETTGMT CQARTSYTED EVLWGHRFFP VI---SLEEG FFKVDYSQFH ATFEVP-TPP YSVKEQEEML LMSSP----- ---LIAP--- --AITNSKER HNSVECLDGL
HS-Kcnj3/G      -EQFEIVVIL EGIVETTGMT CQARTSYTED EVLWGHRFFP VI---SLEEG FFKVDYSQFH ATFEVP-TPP YSVKEQEEML LMSSP----- ---LIAP--- --AITNSKER HNSVECLDGL
GG-Kcnj3/G      -EQFEIVVIL EGIVETTGMT CQARTSYTED EVLWGHRFFP VI---SLEEG FFKVDYSQFH ATFEVP-TPP YSVKEQEEML LMSSP----- ---LIAP--- --AVSNSKER NNSVECLDGL
```

Alignment: HS-IRK(continue)

```
                ....|....| ....|....| ....|....| ....|....| ....|....| ....|....| ....|....| ....|....| ....|....| ....|....| ....|....| ....|....|
                      485        495        505        515        525        535        545        555        565        575        585        595
CI-IRK          -ANFEVILIL EGMVEATGMT TQARASYLPN EILWGHRFQN MIS--FTRNN GYKVNFGKFN KTYMTPNSPH QSAKYLSCAS NSE--MNTLV ---------- ---------- -ESSMNNNKK
HR-TuIRKA       -ANFEVIVIL EGMVEATGMT TQARASYVPD EIMWGHRFEN VIT--FSRTS RYNVDFRKFD RSYEVPETPK CSSKYLQDMQ NADTVSERIM ---------- ---------- -LQDKEQNRK
HS-Kcnj2        -ADFEIVVIL EGMVEATAMT TQCRSSYLAN EILWGHRYEP VL---FEEKH YYKVDYSRFH KTYEVPNTPL CSARDLA-EK KYIL------ ---------- ---------- ---SNA-NSF
HS-Kcnj12       -DDFEIVVIL EGMVEATAMT TQARSSYLAN EILWGHRFEP VL---FEEKN QYKIDYSHFH KTYEVPSTPR CSAKDLV-EN KFLL------ ---------- ---------- ----PSANSF
HS-Kcnj4        -EDFEIVVIL EGMVEATAMT TQARSSYLAS EILWGHRFEP VV---FEEKS HYKVDYSRFH KTYEVAGTPC CSARELQ-ES KITVLPAP-- ---------- ---------- ---PPPPSAF
HS-Kcnj14       -ADFELVVIL EGMVEATAMT TQCRSSYLPG ELLWGHRFEP VL---FQRGS QYEVDYRHFH RTYEVPGTPV CSAKELD-ER AEQASHSLKS ---------- ---------- -SFPGSLTAF
HS-Kcnj16       -DNFEILVTF IYTGDSTGTS HQSRSSYVPR EILWGHRFND VL---EVKRK YYKVNCLQFE GSVEVY-APF CSAKQLDWKD QQLH-IEKAP ---------- ---------- -PVRESCTSD
```

Alignment: HS-IRK/AR(continue)

```
                ....|....| ....|....| ....|....| ....|....| ....|....| ....|....| ....|....| ....|....| ....|....| ....|....| ....|....| ....|....|
                      485        495        505        515        525        535        545        555        565        575        585        595
CI-IRK/AR       -NRFEIVFIL TGTLESTGMT MHAQTSYLSS EIDYGHRFAP ILS-KNYAKS QYEVNFKRFH DTVPEPISLR NYDVTNDSTL NGRS------ ---------- ---------- ----------
HS-Kcnj13/AR    --HFELVVFL SAMQEGTGEI CQRRTSYLPS EIMLHHCFAS LLT--RGSKG EYQIKMENFD KTVPEFPTP- -LVSK----- ---------- ---------- ---------- ----------
HS-Kcnj1a/AR    QQDFELVVFL DGTVESTSAT CQVRTSYVPE EVLWGYRFAP IVS--KTKEG KYRVDFHNFS KTVEVETPHC AMCLY----- ---------- ---------- ---------- ----------
HS-Kcnj10/AR    -GDFELVLIL SGTVESTSAT CQVRTSYLPE EILWGYEFTP AIS--LSASG KYIADFSLFD QVVKVASPSG LRDSTVRYG- ---------- ---------- ---------- ----------
HS-Kcnj15/AR    EKEFELVVLL NATVESTSAV CQSRTSYIPE EIYWGFEFVP VVS--LSKNG KYVADFSQFE QIRKS-PDCT FYCA------ ---------- ---------- ---------- ----------
```

Alignment: GIRK(continue)

```
              ....|....| ....|....| ....|....| ....|....| ....|....| ....|....| ....|....| ....|....| ....|....| ....|....| ....|....| ....|....|
                     605        615        625        635        645        655        665        675        685        695        705        715
CI-GIRKB/G    ---------A KNTSNKLNAQ PPQSVFTVRK SIHQKSLC-- ---------- -------TSN SILTSCDEQQ QRASSSGNFK INSSTRRAVS DANVSATVAV ENENVPESGS DVIPRRMNLL
HR-TuGIRKB    ---------- ---------- ---------- ---------- ---------- ---------- -------TKQ WHPGSSG--- ---------- --YVGNTAA- ---------- ----------
CI-GIRKA/G    ---------G GKEV------ ---------- ---------- ---------- ---------- ---------- ---------- ---------- ---------- ---------- ----------
HR-TuGIRKAa   ---------A NNSPGALSMH RTLPRSPRVQ ESASLSSSFP TPTVRRKKTS IANNCYPDEV EENNKSDSPG IEKTNVQSNE NNNNSLQPVL ASYPVHSTLG QQQASSQSEA DLLRLNKRLA
HS-Kcnj5/G    ---------Y LPSPPLLGGC AEAG--LDAE AE-------- ---------- ------QNEE DEPKGLGGSR EARGSV---- ---------- ---------- ---------- ----------
MM-Kcnj5/G    ---------Y LPSPPLLGGC AEAG--NEAE AE-------- ---------- ------KDEE GEPNGLSVSQ ATRGSM---- ---------- ---------- ---------- ----------
GG-Kcnj5/G    ---------S ISSATVLGGG REAETVRGEE EE-------- ---------- ------EEED REPAAFSGAN GTAGEVKEDL PV-------- ---------- ---------- ----------
HS-Kcnj6/G    ---------S WSVSSKLNQH AELE--TEEE EK-------- ---------- -----NLEEQ TERNGDVANL ENESKV---- ---------- ---------- ---------- ----------
MM-Kcnj6/G    ---------S WSVSSKLNQH AELE--TEEE EK-------- ---------- -----NPEEL TERNGDVANL ENESKV---- ---------- ---------- ---------- ----------
RN-Kcnj6/G    ---------S WSVSSKLNQH AELE--TEEE EK-------- ---------- -----NPEEL TERNGDVANL ENESKV---- ---------- ---------- ---------- ----------
GG-Kcnj6/G    ---------T WSVSSKLDQH AELE--TEEE EK-------- ---------- -----NQEDQ NERNGDVANL ENESKV---- ---------- ---------- ---------- ----------
MM-Kcnj9/G    ---------Y WSIPSRLDEK VEEEGAGEGA GA-------- ---------- -----GDGAD KEHNGCLPPP ESESKV---- ---------- ---------- ---------- ----------
RN-Kcnj9/G    ---------Y WSIPSRLDEK VEEEGAGEGA GA-------- ---------- -----GDGAD KEQNGCLPPP ESESKV---- ---------- ---------- ---------- ----------
HS-Kcnj9/G    ---------Y WSIPSRLDEK VEEEGAGEGA GG-------- ---------- -----EAGAD KEQNGCLPPP ESESKV---- ---------- ---------- ---------- ----------
MM-Kcnj3/G    DD------IS TKLPSKLQKI TGR--EDFPK KLLRMSST-- ---------- -----TSEKA YSLGDLPMKL QRISSVPG-- NSEEKLVSKT TKMLSDPMSQ SVADLPP-KL QKMAGGPT--
RN-Kcnj3/G    DD------IS TKLPSKLQKI TGR--EDFPK KLLRMSST-- ---------- -----TSEKA YSLGDLPMKL QRISSVPG-- NSEEKLVSKT TKMLSDPMSQ SVADLPP-KL QKMAGGPT--
HS-Kcnj3/G    DD------IT TKLPSKLQKI TGR--EDFPK KLLRMSST-- ---------- -----TSEKA YSLGDLPMKL QRISSVPG-- NSEEKLVSKT TKMLSDPMSQ SVADLPP-KL QKMAGGAA--
GG-Kcnj3/G    DE------VG IKLPSKLQKI TGR--DDFPK KLLRMSST-- ---------- -----TSEKA YSMGDLPMKL QRISSVPG-- NSEEKLVSKA TKMMSDPMSQ SVADLPP-KL QKLSGG-G--
```

Alignment: HS-IRK(continue)

```
              ....|....| ....|....| ....|....| ....|....| ....|....| ....|....| ....|....| ....|....| ....|....| ....|....| ....|....| ....|....|
                     605        615        625        635        645        655        665        675        685        695        705        715
CI-IRK        NRQADSESSS ISASDGKDSG YTAVEDLTRA Y------SSQ TLN------- ------TEN- -----NKVVS ---------- ---------- ---------- ---------- ----------
HR-TuIRKA     NTSKDATRED FSVSDGRDSG YTAPEDYQAT D------SNK TLNSTSTCGS SYINHDTENR RRSWTKKAIE DTMNKNSNQY ESTNMDKCQT SSDILPNMKQ DMQIKTNRNS ADMTAQMTLT
HS-Kcnj2      CYEN-----E VALTSKEED- --DSENGVPE S--------- -TSTDT---- -----PPDID LHNQASVPLE PRPLRRESEI ---------- ---------- ---------- ----------
HS-Kcnj12     CYEN-----E LAFLSRDEED EADGDQDGRS R--------- ------DGLS PQ--ARHDFD RLQAGGGVLE QRPYRRESEI ---------- ---------- ---------- ----------
HS-Kcnj4      CYEN-----E LALMSQEEEE MEEEAAAAAA VAAGLGLEAG SKEEAGIIRM LEFGSHLDLE RMQAS-LPLD NISYRRESAI ---------- ---------- ---------- ----------
HS-Kcnj14     CYEN-----E LALSCCQEED EDDETEEGNG V--------- ---------- ------ETED GAASPRVLTP TLALTLPP-- ---------- ---------- ---------- ----------
HS-Kcnj16     TKAR-----R RSFSAVAIVS SCENPEETTT S--------- ---------- ----ATHEYR ETPYQKALLT LNRISVESQM ---------- ---------- ---------- ----------
```

Alignment: HS-IRK/AR(continue)

```
              ....|....| ....|....| ....|....| ....|....| ....|....| ....|....| ....|....| ....|....| ....|....| ....|....| ....|....| ....|....|
                     605        615        625        635        645        655        665        675        685        695        705        715
CI-IRK/AR     --------VV TSLIEETEED IISVSDEVFS AV-------- ---------- ---------- ---------- ---------- ---------- ---------- ---------- ----------
HS-Kcnj13/AR  ---------S PNRTDLDIHI NG---QSIDN FQI------- ---------- ------SETG LTE------- ---------- ---------- ---------- ---------- ----------
HS-Kcnj1a/AR  ---------N EKD-VRARMK RG---YDNPN FIL------- ---------- ------SEVN ETDDTKM--- ---------- ---------- ---------- ---------- ----------
HS-Kcnj10/AR  ---------D PEKLKLEESL R--EQAEKE- ---------- ---------- ------GSAL SVRISNV--- ---------- ---------- ---------- ---------- ----------
HS-Kcnj15/AR  ---------D SEKQQLEEKY RQEDQRERE- ---------- ---------- ------LRTL LLQQSNV--- ---------- ---------- ---------- ---------- ----------
```
