# Supplementary material for: Ancient intron insertion sites and palindromic genomic duplication evolutionally shapes an elementally functioning membrane protein family
Source: BMC Evol Biol. 2007 Aug 20;7:143. doi: 10.1186/1471-2148-7-143 (PMC1999503; doi:10.1186/1471-2148-7-143)
Supplement: Additional file 9 — Alignment of G-protein beta AA sequences. Alignment of a tunicate, two Caenorhabditis and Anopheles, a Drosophila, and six vertebrate GNB1 AA sequences. Another alignment of a tunicate, two Caenorhabditis, two Anopheles, a Drosophila, and seven vertebrate GNB5 AA sequences is also included. For the explanation of colored characters, see the legend of Fig. 16 in the original paper. [file 1471-2148-7-143-S9.pdf]

Alignment: GNB1-Intron-insertion

```
                    ....|....| ....|....| ....|....| ....|....| ....|....| ....|....| ....|....| ....|....| ....|....| ....|....| ....|....| ....|....|
                        5         15         25         35         45         55         65         75         85         95         105        115
MM-GNB1             MS-------E LDQLRQEAEQ LKNQIRDARK ACADATLSQI TNNIDPVGRI QMRTRRTLRG HLAKIYAMHW GTDSRLLVSA SQDGKLIIWD SYTTNKVHAI PLRSSWVMTC AYAPSGNYVA
HS-GNB1             MS-------E LDQLRQEAEQ LKNQIRDARK ACADATLSQI TNNIDPVGRI QMRTRRTLRG HLAKIYAMHW GTDSRLLVSA SQDGKLIIWD SYTTNKVHAI PLRSSWVMTC AYAPSGNYVA
RN-GBB1-RAT         MS-------E LDQLRQEAEQ LKNQIRDARK ACADATLSQI TNNIDPVGRI QMRTRRTLRG HLAKIYAMHW GTDSRLLVSA SQDGKLIIWD SYTTNKVHAI PLRSSWVMTC AYAPSGNYVA
GG-GNB/A3           MS-------E LDQLRQEAEQ LKNQIRDARK ACADATLAQI TANIDPVGRI QMRTRRTLRG HLAKIYAMHW GTDSRLLVSA SQDGKLIIWD SYTTNKVHAI PLRSSWVMTC AYAPSGNYVA
FR-GNB1             MS-------E LDQLRQEAEQ LKNQIRDARK ACADATLSQI TANIDPVGRI QMRTRRTLRG HLAKIYAMHW GTDSRLLVSA SQDCKLIIWD SYTTNKVHAI PLRSSWVMTC AYAPSGNYVA
DR-gnb1             ---------- ---------- ------DARK ACADATLSQI TANIDPVGRI QMRTRRTLRG HLAKIYAMHW GTDSRLLVSA SQDGKLIIWD SYTTNKVHAI PLRSSWVMTC AYAPSGNYVA
CI-GNB1/ci0100145646 MSNQTNSQQH VEELRNEAEN IKKEIRDKRQ ALKDSSLSEV ASNVDAIGRV QMRTRRTLRG HLAKIYAMHW GTDSRHVVSA SQDGKLILWD SYTTNKVHAI PLRSSWVMTC AYAPSGSFVA
CE-gpb-1            MS-------E LDQLRQEAEQ LKSQIREARK SANDTTLATV ASNLEPIGRI QMRTRRTLRG HLAKIYAMHW ASDSRNLVSA SQDGKLIVWD SYTTNKVHAI PLRSSWVMTC AYAPSGSFVA
CB-CBG03131         MS-------E LDQLRQEAEQ LKSQIREARK SANDTTLATV ASNLEPIGRI QMRTRRTLRG HLAKIYAMHW ASDSRNLVSA SQDGKLIVWD SYTTNKVHAI PLRSSWVMTC AYAPSGSFVA
AN-DMGbeta13F       MN-------E LEALRQEAET LKNAIRDARK AACDTSLVQA TNNLEPIGRI QMRTRRTLRG HLAKIYAMHW GSDSRNLVSA SQDGKLIVWD SHTTNKVHAI PLRSSWVMTC AYAPSGNFVA
DM-Gbeta13F         MN-------E LDSLRQEAES LKNAIRDARK AACDTSLLQA ATSLEPIGRI QMRTRRTLRG HLAKIYAMHW GNDSRNLVSA SQDGKLIVWD SHTTNKVHAI PLRSSWVMTC AYAPSGSYVA
Clustal Consensus                             : *: :  *::*    : .::.:**: ********** ********** ..*** :*** *** ***:** *:******** ********** ******.:**
```

Alignment: GNB1-Intron-insertion (continue)

```
                    ....|....| ....|....| ....|....| ....|....| ....|....| ....|....| ....|....| ....|....| ....|....| ....|....| ....|....| ....|....|
                       125        135        145        155        165        175        185        195        205        215        225        235
MM-GNB1             CGGLDNICSI YNLKTREGNV RVSRELAGHT GYLSCCRFLD DNQIVTSSGD TTCALWDIET GQQTTTFTGH TGDVMSLSLA PDTRLFVSGA CDASAKLWDV REGMCRQTFT GHESDINAIC
HS-GNB1             CGGLDNICSI YNLKTREGNV RVSRELAGHT GYLSCCRFLD DNQIVTSSGD TTCALWDIET GQQTTTFTGH TGDVMSLSLA PDTRLFVSGA CDASAKLWDV REGMCRQTFT GHESDINAIC
RN-GBB1-RAT         CGGLDNICSI YNLKTREGNV RVSRELAGHT GYLSCCRFLD DNQIVTSSGD TTCALWDIET GQQTTTFTGH TGDVMSLSLA PDTRLFVSGA CDASAKLWDV REGMCRQTFT GHESDINAIC
GG-GNB/A3           CGGLDNICSI YNLKTREGNV RVSRELAGHT GYLSCCRFLD DNQIVTSSGD TTCALWDIET GQQTTTFTGH TGDVMSLSLA PDARCFVSGA CDASAKLWDV REGMCRQTFT GHESDINAIC
FR-GNB1             CGGLDNICSI YNLKTREGNV RVSRELAGHT GYLSCCRFLD DNQIVTSSGD TTCALWDIET GQQTTFAGH  TGDVMSLSLA PDTRMFVSGA CDASAKLWDI REGMCRQTFT GHESDINAIC
DR-gnb1             CGGLDNICSI YSLKTREGNV RVSRELAGHT GYLSCCRFLD DNQIVTSSGD TTCALWDIET GQQTTFAGH  TGDVMSLSLA PDTRLFVSGA CDASAKLWDI REGMCRQTFT GHESDINAIC
CI-GNB1/ci0100145646 CGGLDNICSV YNLKTREGNV RVSRELNGHT GYLSCCRFLD DTRIVTSSGD MTCALWDIET GMQTTAFTGH TGDVMSLSVT DDKNTFISGA CDATAKLWDL RDGMCRQTFS GHESDINAVS
CE-gpb-1            CGGLDNICSI YSLKTREGNV RVSRELPGHT GYLSCCRFLD DNQIVTSSGD MTCALWDIET GQQCTAFTGH TGDVMSLSLS PDFRTFISGA CDASAKLWDI RDGMCKQTFP GHESDINAVA
CB-CBG03131         CGGLDNICSI YSLKTREGNV RVSRELPGHT GYLSCCRFLD DNQIVTSSGD MTCALWDIET GQQCTAFTGH TGDVMSLSLS PDFRTFISGA CDASAKLWDI RDGMCKQTFP GHESDINAVA
AN-DMGbeta13F       CGGLDNICSI YNLKTREGNV RVSRELPGHT GYLSCCRFLD DNQIVTSSGD MSCGLWDIET GQQCTSFLGH TGDVMALSLS PQCRVFVSGA CDASAKLWDI REGQCKQTFP GHESDINAVT
DM-Gbeta13F         CGGLDNMCSI YNLKTREGNV RVSRELPGHG GYLSCCRFLD DNQIVTSSGD MSCGLWDIET GLQVTSFLGH TGDVMALSLA PQCKTFVSGA CDASAKLWDI REGVCKQTFP GHESDINAVT
Clustal Consensus   ******:**: *.******** ****** **  ********** *.:******* :*.****** * * *:* ** *****:**:: : . *:*** ***:*****: *:* *:***. ********:
```

First page

Alignment: GNB1-Intron-insertion (continue)

```
                    ....|....| ....|....| ....|....| ....|....| ....|....| ....|....| ....|....| ....|....| ....|....| ....|....| ....|..
                        245        255        265        275        285        295        305        315        325        335        345
MM-GNB1             FFPNGNAFAT GSDDATCRLF DLRADQELMT YSHDNIICGI TSVSFSKSGR LLLAGYDDFN CNVWDALKAD RAGVLAGHDN RVSCLGVTDD GMAVATGSWD SFLKIWN
HS-GNB1             FFPNGNAFAT GSDDATCRLF DLRADQELMT YSHDNIICGI TSVSFSKSGR LLLAGYDDFN CNVWDALKAD RAGVLAGHDN RVSCLGVTDD GMAVATGSWD SFLKIWN
RN-GBB1-RAT         FFPNGNAFAT GSDDATCRLF DLRADQELMT YSHDNIICGI TSVSFSKSGR LLLAGYDDFN CNVWDALKAD RAGVLAGHDN RVSCLGVTDD GMAVATGSWD SFLKIWN
GG-GNB/A3           FFPNGNAFAT GSDDATCRLF DLRADQELMV YSHDNIICGI TSVAFSKSGR LLLAGYDDFN CNVWDTLKAD RAGVLAGHDN RVSCLGVTDD GMAVATGSWD SFLKIWN
FR-GNB1             FFPNGNAFAT GSDDATCRLF DLRADQELMV YSHDNIICGI TSVAFSKSGR LLLAGYDDFN CNVWDTLKAD RAGVLAGHDN RVSCLGVTDD GMAVATGSWD SFLKIWN
DR-gnb1             FFPNGNAFAT GSDDATCRLF DLRADQELMV YSHDNIICGI TSVAFSKSGR LLLAGYDDFN CNVWDTLKAD RAGVLAGHDN RVSCLGVTDD GMAVATGSWD SFLKIWN
CI-GNB1/ci0100145646 MFPNNMAFGT GSDDATCRLF DIRSDQELMI YSNDNIACGI TSVAFSRSGR LFFAGYDDFN CNIWDAMKGD RAGVLAGHDN RVSCLGITVD GMAVATGSWD SFLKVWN
CE-gpb-1            FFPSGNAFAT GSDDATCRLF DIRADQELAM YSHDNIICGI TSVAFSKSGR LLFAGYDDFN CNVWDSMRQE RAGVLAGHDN RVSCLGVTED GMAVCTGSWD SFLKIWN
CB-CBG03131         FFPSGNAFAT GSDDATCRLF DIRADQELAM YSHDNIICGI TSVAFSKSGR LLFAGYDDFN CNVWDSMRQE RAGVLAGHDN RVSCLGVTED GMAVCTGSWD SFLKIWN
AN-DMGbeta13F       FFPNGHAFAT GSDDATCRLF DIRADQELAM YSHDNIICGI TSVAFSKSGR LLLAGYDDFN CNVWDTLKAE RAGILAGHDN RVSCLGVTEN GMAVATGSWD SFLRVWN
DM-Gbeta13F         FFPNGQAFAT GSDDATCRLF DIRADQELAM YSHDNIICGI TSVAFSKSGR LLLAGYDDFN CNVWDTMKAE RSGILAGHDN RVSCLGVTEN GMAVATGSWD SFLRVWN
Clustal Consensus   :**.. **.* ********** *:*:****   **:*** *** ***:**:*** *::******* **:**:::  : *:*:****** ******:* : ****.***** ***::**
```

Second page

Alignment: GNB5-Intron-insertion

```
                    ....|....| ....|....| ....|....| ....|....| ....|....| ....|....| ....|....| ....|....| ....|....| ....|....| ....|....| ....|....|
                        5         15         25         35         45         55         65         75         85         95        105        115
RN-GBB5/A1          MCDQTFLVNV FGSCDKCFKQ RALRPVFKKS QQLNYCSPCA EIMATDGLHE NETLASLKSE AESLKGKLEE ERAKLHDVE- ---------- ---------- --LHQVAERV EALGQFVMKT
MM-GNB5/A1          MCDQTFLVNV FGSCDKCFKQ RALRPVFKKS QQLNYCSTCA EIMATDGLHE NETLASLKSE AESLKGKLEE ERAKLHDVE- ---------- ---------- --LHQVAERV EALGQFVMKT
HS-GNB5Lb           MCDQTFLVNV FGSCDKCFKQ RALRPVFKKS QQLSYCSTCA EIMATEGLHE NETLASLKSE AESLKGKLEE ERAKLHDVE- ---------- ---------- --LHQVAERV EALGQFVMKT
GG-GNB5/A1          MCDQTFLAIV FGPCDKCSNE KPLRAVYIKS EQLSYCSLCV EMMATEGLHE NETLASLKNE AESLKGKLEE ERAKLHDVE- ---------- ---------- --LHQVAERV EALGQFVMKT
FR-GNB5/A           ---------- ---------- ---------- ---------- --MATPEVQL NETLAHLKTE SEALKSKLEE ERAKLHDVE- ---------- ---------- --LHQVAEKV EGLGQFVMKT
DR-zgc73196/GNB5    MCDQTFLAIT FGPCDKCSEN KPLMNIYLKN EPINYCSLCV EMMACQGLAK GETVQSLKAE SESLKAKLEE ERAKLHDVE- ---------- ---------- --LHQVAEKM EALGQFVMKT
FR-GNB5/B           MCDQTFVAAT FGPCDSCANP SPLMNIYIKN EPINYCSFCV EMMACRELQR GETLASLKRE SDTLKKKLEE ERGKLNDVE- ---------- ---------- --LHQVAEKV DTLGALSLKT
CI-GNB5             ---------- ---------- ---------- ---------- -MASDKNSTR YDTLENLRKE AEQLRKKLQA DRRNLNDAE- ---------- ---------- --LFVMGKKA GGSPSIQVRC
CE-EAT-11           ---------- ---------- ---------- -------MPE NSQPTTTEKG SEYLEQLANE AEELRKKLDQ ERHKLNDIP- ---------- ---------- --IQQAAERL DVMGALGVKQ
CB-CBG11904         ---------- ---------- ---------- ---------- ---------- SEYLEQLANE AEELRKKLDA ERHKLNDIP- ---------- ---------- --IQQAAERL DVMGALGVKQ
AN-DMGbeta5/A1      ---------- ---------- ---------- ---------- ---------- SDKVSALIKE AECLKSKLEE ERQKLNDVT- ---------- ---------- --LSSLAERL EMISYLNIKP
AN-DMGbeta5/A2      ---------- ---------- ---------- ------MMAE VLTS---LQH SDKVSALIKE AECLKSKLEE ERQKLNDVTC IMIIVIDNRT NISINFCFTF ISVSSLAERL EMISYLNIKP
DM-Gbeta5           ---------- ---------- ---------- -----MSEAA VPASNANANA TEKMASLVRE AENLKTKLEE ERQKLNDVN- ---------- ---------- --LSNIAERL EQIAYVNIKP
Clustal Consensus                                                          : :  *  * :: *: **:  :* :*:*                           :   .::       . ::
```

Alignment: GNB5-Intron-insertion (continue)

```
                    ....|....| ....|....| ....|....| ....|....| ....|....| ....|....| ....|....| ....|....| ....|....| ....|....| ....|....| ....|....|
                      125        135        145        155        165        175        185        195        205        215        225        235
RN-GBB5/A1          RRTLKGHGNK VLCMDWCKDK RRIVSSSQDG KVIVWDSFTT NKEHAVT-MP CTWVMACAYA PSGCAIACGG LDNKCSVYPL TFDKN----- ---------- -------ENM AAKKKSVAMH
MM-GNB5/A1          RRTLKGHGNK VLCMDWCKDK RRIVSSSQDG KVIVWDSFTT NKEHAVT-MP CTWVMACAYA PSGCAIACGG LDNKCSVYPL TFDKN----- ---------- -------ENM AAKKKSVAMH
HS-GNB5Lb           RRTLKGHGNK VLCMDWCKDK RRIVSSSQDG KVIVWDSFTT NKEHAVT-MP CTWVMACAYA PSGCAIACGG LDNKCSVYPL TFDKN----- ---------- -------ENM AAKKKSVAMH
GG-GNB5/A1          RRTLKGHGNK VLCMDWCKDK RRIVSSSQDG KVIVWDSFTT NKEHAVT-MP CTWVMACAYA PSGCAIACGG LDNKCSVYPL TFDKN----- ---------- -------ENM AAKKKSVAMH
FR-GNB5/A           RRTLKGHGNK VLCMDWCKDK RRIVSSSQDG KVIVWDAFTT NKEHAVT-MP CTWVMACAYA PSGCAVACGG LDNKCSVYPL SLDKN----- ---------- -------ENL AAKKKSVAMH
DR-zgc73196/GNB5    RRTLKGHGNK VLCMDWCRDK RRIVSSSQDG KVIVWDAYTT NKEHAVT-MP CTWVMACAYA PSGCAVACGG LDNKCSVYPL SLDKN----- ---------- -------ENL ASKKKSVAMH
FR-GNB5/B           KRVLKGHGNK VLCMDWCKDK RRLVSSSQDG KVILWDAFTL NKEHAVS-LP CTWVLACAYA PSGCAVACGG LDNKCCVIPL SLDKN----- ---------- -------ENL AAKKKSVAMH
CI-GNB5             RRILKGHTGK VLDMDWSLDK RRIVSSSQDG KILVWDGFTT NKASFLINLP TTWVNACAYA PSGGSIACGG LDNKCSVFPL NSSSNSSTLS VSNKQNPVSS GPADTGAGTI MQQRKVVAMH
CE-EAT-11           RRILKGHVGK VLCMDWSLDK RHIVSSSQDG KVIVWDGFTT NKEHALT-MP TTWVMACAFS PSSQMIACGG LDNKCSVVPL SFE------- ---------- -------DDI IQKKRQVATH
CB-CBG11904         RRILKGHVGK VLCMDWSLDK RHIVSSSQDG KVIVWDGFTT NKEHALT-MP TTWVMACAFS PSSQMIACGG LDNKCSVVPL SFE------- ---------- -------DDI IQKKRQVATH
AN-DMGbeta5/A1      RRVLKGHQAK VLCSNWSPDK RHIVSSSQDG KLIIWDAFTT NKEHAVT-MP TTWIMGCSYA PSGNLVACGG LDNKVTVYPI TLE------- ---------- -------EDI SSRKKTVGTH
AN-DMGbeta5/A2      RRVLKGHQAK VLCSNWSPDK RHIVSSSQDG KLIIWDAFTT NKEHAVT-MP TTWIMGCSYA PSGNLVACGG LDNKVTVYPI TLE------- ---------- -------EDI SSRKKTVGTH
DM- Gbeta5          RKVLKGHQAK VLCTDWSPDK RHIISSSQDG RLIIWDAFTT NKEHAVT-MP TTWIMACAYA PSGNFVACGG LDNKVTVYPI TSD------- ---------- -------EEM AAKKRTVGTH
Clustal Consensus   :: ****  * **  :*. ** *:::****** ::::**.:*  **   :  :*  **: .*::: **.  :**** ****  * *: . .                           :   ::: *. *
```

Third page

Alignment: GNB5-Intron-insertion (continue)

```
                    ....|....| ....|....| ....|....| ....|....| ....|....| ....|....| ....|....| ....|....| ....|....| ....|....| ....|....| ....|....|
                         245        255        265        275        285        295        305        315        325        335        345        355
RN-GBB5/A1          TNYLSACSFT NSDMQVSAPI PLG-HSPCRA HLGNTGNSQK KGGASCALLL FSPGKVSFLR LTGWRRGCDK KAMVWDMRSG QCVQAFETHE SDVNSVRYYP SGDAFASGSD DATCRLYDLR
MM-GNB5/A1          TNYLSACSFT NSDMQILTAS GDGTCALWDV ESGQLLQSFH GHGADVLCLD LAPSETGNTF VSG---GCDK KAMVWDMRSG QCVQAFETHE SDVNSVRYYP SGDAFASGSD DATCRLYDLR
HS-GNB5Lb           TNYLSACSFT NSDMQILTAS GDGTCALWDV ESGQLLQSFH GHGADVLCLD LAPSETGNTF VSG---GCDK KAMVWDMRSG QCVQAFETHE SDINSVRYYP SGDAFASGSD DATCRLYDLR
GG-GNB5/A1          TNYLSACSFT NSDMQILTAS GDGTCALWDV ESGQLLQSFH GHGADVLCLD LAPSETGNTF VSG---GCDK KAMVWDMRSG QCIQSFETHD SDINSVRYYP SGDAFASGSD DATCRLYDLR
FR-GNB5/A           TNYLSACSFT NSDMQILTSS GDGTCALWDV ESGQLLQSFH GHAADVLCLD LAPSETGNTF VSG---GCDK KANVWDMRSG QCIQSFETHE SDINSVRYYP SGDAFASGSD DATCRLYDLR
DR-zgc73196/GNB5    TNYLSSCSFT KSDMQILTSS GDGTCALWDV ESGQLLQSFH GHSADVLSLD LAPSETGSTF VSG---GCDK KANVWDMRSG QNVQSFETHD SDINSVKYYP SGDAFASGSD DATCRLYDLR
FR-GNB5/B           TNYVSGCSFV NTDMQILTSS GDGTCALWDV ESGQLLQSFH GHTADVLSLD FIPSETGNIF ISG---GCDK KANVWDMRSG QNIQSFENHV SDVNCVKFHP SGDAFASASD DATCRFYDLR
CI-GNB5             TSYISACTFT HSDYQILT-S GDSTCALWDV ESGQLLQSFH GHQSDVMDAA LSPCETGNLF ISG--AGCDK NACVWDMRTA KCIQSFQTHN SDINTVKWFP TGEAFATGSD DGTIKMYDLR
CE-EAT-11           TSYMSCCTFL RSDNLILTGS GDSTCAIWDV ESGQLIQNFH GHTGDVFAID VPKCDTGNTF ISA---GADK HSLVWDIRSG QCVQSFEGHE ADINTVRFHP NGDAFATGSD DATCRLFDLR
CB-CBG11904         TSYMSCCTFL RSDNLILTGS GDSTCAIWDV ESGQLIQNFH GHTGDVFAID VPKCDTGNTF ISA---GADK HSLVWDIRSG QCVQSFEGHE ADINTVRFHP NGDAFATGSD DATCRLFDLR
AN-DMGbeta5/A1      TSYMSCCIFP NSDQQILTGS GDSTCALWDV ESGQLLQSFH GHTGDVMSID LAPNETGNTF VSG---SCDK MAFIWDMRSG HVVQSFEGHQ SDINSVKFHP SGDAISTGSD DSTCRLFDMR
AN-DMGbeta5/A2      TSYMSCCIFP NSDQQILTGS GDSTCALWDV ESGQLLQSFH GHTGDVMSID LAPNETGNTF VSG---SCDK MAFIWDMRSG HVVQSFEGHQ SDINSVKFHP SGDAISTGSD DSTCRLFDMR
DM- Gbeta5          TSYMSCCIYP NSDQQILTGS GDSTCALWDV ESGQLLQSFH GHSGDVMAID LAPNETGNTF VSG---SCDR MAFIWDMRSG HVVQSFEGHQ SDVNSVKFHP CGDAIATGSD DSSCRLYDMR
Clustal Consensus   *.*:* * :  .:*  : :     .   :   . . *:  :. :     ..        .    ...    ::.    ..*:  :  :**:*:.  : :*:*: *  :*:* *::.*  *:*::.** *.:  :::*:*
```

Alignment: GNB5-Intron-insertion (continue)

```
                    ....|....| ....|....| ....|....| ....|....| ....|....| ....|....| ....|....| ....|....| ....
                         365        375        385        395        405        415        425        435
RN-GBB5/A1          ADREVAIYSK ESIIFGASSV DFSLSGRLLF AGYNDYTINV WDVLKGSRVS ILFGHENRVS TLRVSPDGTA FCSGSWDHTL RV--
MM-GNB5/A1          ADREVAIYSK ESIIFGASSV DFSLSGRLLF AGYNDYTINV WDVLKGSRVS ILFGHENRVS TLRVSPDGTA FCSGSWDHTL RVWA
HS-GNB5Lb           ADREVAIYSK ESIIFGASSV DFSLSGRLLF AGYNDYTINV WDVLKGSRVS ILFGHENRVS TLRVSPDGTA FCSGSWDHTL RVWA
GG-GNB5/A1          ADREVAIYSK ESIIFGASSV DFSLSGRLLF AGYNDYTINV WDVLKGSRVS ILFGHENRVS TLRVSPDGTA FCSGSWDHTL RV--
FR-GNB5/A           ADREVAIYSK ESIIFGVSSV DFSLSGRLLF GGYNDYTINV WDVLKGTRVS ILFGHENRVS TLRVSPDGTA FCTGSWDHTL RV--
DR-zgc73196/GNB5    ADREVAIYSK DSIIFGASSV DFSLSGRLLF AGYNDYNINV WDVLKGTRVA ILFGHENRVS TVRVSPDGTA FCSGSWDNTL RIWA
FR-GNB5/B           ADREVAVYQK DSIIFGASSL DFSLSGRLLF TGYNDYTINV WDVLKGSRVS VLFGHENRIS RVRVSPDGTA LCSASWDSTL RV--
CI-GNB5             ADREIACYER PNVLFGVNSV DFSVSGRIVL GGYNDYLVHV WDTITGEKLT ALFGHENRIS CLKMSPDGTS FCTGSWDTTL RIWA
CE-EAT-11           ADRQVCVYEK ESILFPVNGV DFSLSGRILF AGYGDYRVGV WDSLKCARHS VLYGHENRIS CLRTSPDGTA VCSASWDCTI RIWA
CB-CBG11904         ADRQVCVYEK ESILFPVNGV DFSLSGRILF AGYGDYRVGV WDSLKCVRHS VLYGHENRIS CLRTSPDGTA VCSASWDCTI RIWA
AN-DMGbeta5/A1      ADKEVAVFCK DSIIFGVNCV DFSVSGRLLF AGYNDYTVNV WDTLKAQRVC LLYGHENKVS CLQVSPDGTA LSTGSWDYTL RVKY
AN-DMGbeta5/A2      ADKEVAVFCK DSIIFGVNCV DFSVSGRLLF AGYNDYTVNV WDTLKAQRVC LLYGHENKVS CLQVSPDGTA LSTGSWDYTL ----
DM-Gbeta5           ADREVAVFAK ESIIFGVNSV DFSVSGRLLF AGYNDYTVNL WDTLKSERVC LLYGHENKVS CVQVSPDGTA LSTGSWDYTI RVWA
Clustal Consensus   **:::.  : :  .::* ..  : ***:***:::  **.** : : ** :.   :    *:****::*  :: *****:  ..:.*** *:
```

Forth page
